# I Brazilian guideline on hypertension in dialysis of the Brazilian Society of Nephrology

**DOI:** 10.1590/2175-8239-JBN-2024-0033en

**Published:** 2025-02-24

**Authors:** Cibele Isaac Saad Rodrigues, Sebastião Rodrigues Ferreira-Filho, Ana Flávia de Souza Moura, Carlos Eduardo Poli-de-Figueiredo, Dirceu Reis da Silva, Fernanda Salomão Gorayeb Polacchini, Fernando Antônio de Almeida, Maria Eliete Pinheiro, Rodrigo Bezerra, Rogério Baumgratz de Paula, Aldo José Peixoto, Ana Elizabeth Prado Lima Figueiredo, Audes Diógenes Magalhães Feitosa, Carlos Alberto Machado, Celso Amodeo, Décio Mion, Elizabeth Silaid Muxfeldt, Giovanio Vieira da Silva, José Andrade Moura-Neto, José Muniz Pazeli, Leda Daud Lotaif, Luciano F. Drager, Luis Cuadrado Martín, Luiz Aparecido Bortolotto, Marcus Gomes Bastos, Marcus Vinícius Bolívar Malachias, Marcos Vinícius Paiva Cavalcanti Moreira, Maria Eugenia Fernandes Canziani, Roberto Dischinger Miranda, Roberto Jorge da Silva Franco, Roberto Pecoits, Rogerio Andrade Mulinari, Rosilene Motta Elias, Weimar Kunz Sebba Barroso, Wilson Nadruz

**Affiliations:** 1 Brazilian Society of Nephrology, São Paulo, SP, Brazil.; 2 Brazilian Society of Hypertension, São Paulo, SP, Brazil.; 3 Pontifícia Universidade Católica de São Paulo, Sorocaba, SP, Brazil.; 4 Universidade Federal de Uberlândia, Uberlândia, MG, Brazil.; 5 Escola Bahiana de Medicina e Saúde Pública, Salvador, BA, Brazil.; 6 Pontifícia Universidade Católica do Rio Grande do Sul, Porto Alegre, RS, Brazil.; 7 Hospital de Clínicas de Porto Alegre, Porto Alegre, RS, Brazil.; 8 Hospital de Base de São José do Rio Preto, São José do Rio Preto, SP, Brazil.; 9 Universidade Federal de Alagoas, Alagoas, AL, Brazil.; 10 Universidade Federal de Pernambuco, Recife, PE, Brazil.; 11 Universidade Federal de Juiz de Fora, Juiz de Fora, MG, Brazil.; 12 Yale University, Yale School of Medicine, New Haven, United States.; 13 Brazilian Society of Cardiology, São Paulo, SP, Brazil.; 14 Secretaria Municipal de Saúde de Campos do Jordão, Campos do Jordão, SP, Brazil.; 15 Hospital do Coração da Associação Beneficente Síria de São Paulo, São Paulo, SP, Brazil.; 16 Universidade de São Paulo, São Paulo, SP, Brazil.; 17 Universidade Federal do Rio de Janeiro, Rio de Janeiro, RJ, Brazil.; 18 Faculdade de Medicina de Barbacena, Barbacena, MG, Brazil.; 19 Universidade Estadual Paulista, Botucatu, SP, Brazil.; 20 Faculdade de Ciências Médicas de Minas Gerais, Belo Horizonte, MG, Brazil.; 21 Universidade Federal de São Paulo, São Paulo, SP, Brazil.; 22 Pontifícia Universidade Católica do Paraná, Curitiba, PR, Brazil.; 23 Arbor Research Collaborative for Health, Ann Arbor, United States.; 24 Universidade Federal do Paraná, Curitiba, PR, Brazil.; 25 Universidade Nove de Julho, São Paulo, SP, Brazil.; 26 Universidade Federal de Goiás, Goiania, GO, Brazil.; 27 Universidade Estadual de Campinas, Campinas, SP, Brazil.

**Keywords:** Hypertension, Kidney Dialysis, Peritoneal Dialysis, Risk Factors; Pharmacological Treatment, Clinical Practice Guidelines as Topic, Chronic Kidney Failure, Renal Hypertension, Blood Pressure Determination, Chronic Kidney Insufficiency

## Abstract

Hypertension in dialysis patients (HTND) has a high prevalence, affecting at least 80% or more of patients, and its management in the nephrology practice is heterogeneous and often empirical. Knowing how to define, understand the pathophysiology, diagnose, monitor and treat with lifestyle changes, and adjust antihypertensive drugs to achieve the recommended blood pressure (BP) target - to reduce morbidity and mortality - requires specific knowl­edge and approaches within the contexts of hemodialysis (HD) and peritoneal dialysis (PD). This document is the first guideline of the Brazilian Society of Nephrology, developed by the departments of Hypertension and Dialysis. It aims to guide physicians who provide care in dialysis centers on how to manage patients with HTND, in a comprehensive and individualized manner, based on the critical appraisal of the best available scientific evidence. When such evidence is scarce or unavailable, the opinion of specialists should be recommended. The different topics covered include HTND definition (pre-HD BP ≥ 140/90 mmHg and post-HD BP ≥ 130/80 mmHg), epidemiology, and pathophysiology; diagnosis of HTND preferably with BP measurements outside the dialysis setting (BP ≥ 130/80 mmHg); complementary assessment; blood pressure targets; non-pharmacological treatment; use of the most appropriate antihypertensive medications; special situations; and complications of HTND, predominantly cardiovascular ones.

## Grade of Recommendations and Levels of Evidence^1^

The recommendations were stratified into Classes or Grades I, IIa, IIb, or III and levels of evidence, described as follows:

### Classes (Degrees) of Recommendation

Class I – Conditions for which there is conclusive evidence, or if not, general agreement that a given treatment or procedure is safe/beneficial and useful/effective;

Class II – Conditions for which there is conflicting evidence and/or differing opinions about the safety and benefit/efficacy of the given treatment or procedure;

Class IIA – The majority of evidence/opinion is in favor of the given treatment or procedure. The majority approves;

Class IIB – Safety and benefit/efficacy is less well established, with no predominance of opinion;

Class III – Conditions for which there is evidence and/or consensus that the procedure or treatment is not useful/effective and in some cases may even be harmful.

### Levels of Evidence

Level A – Data from multiple welldesigned, concordant randomized controlled trials and/or robust metaanalysis of randomized clinical studies;

Level B – Data from a less robust meta-analysis, from a single randomized study or from large non-randomized (observational) studies;

Level C – Data from the consensus of expert opinions and/or small studies, retrospective trials, and registries.

## Introduction

The primary objective of this publication is to provide nephrologists caring for chronic kidney disease patients on dialysis with the best available scientific evidence on the different aspects of HTN, from its pathophysiology to treatment. This is an unprecedented document in Portuguese language, designed to be useful to nephrologists in their daily clinical practice.

The SBN departments involved in this initiative aim for the recommendations and suggestions expressed here to have national repercussions, contributing to a better approach and treatment for individuals requiring dialysis therapies, with consequent benefits in reducing CV and renal morbidity and mortality.

It is a fact that even the prestigious KDIGO (Kidney Diseases Improving Global Outcomes), in its guidelines on HTN in CKD, did not postulate recommendations for stage 5D. This is likely due to the lack of randomized controlled trials, and systematic reviews and meta-analyses in this population that could support recommendations with high levels of confidence and quality.

Currently in Brazil, according to the 2022 Census of the Brazilian Society of Nephrology, there are 872 dialysis centers, with an estimated prevalence of 153,831 patients in stage 5D, with 91% on HD. HTN is identified as the direct cause responsible for CKD 5D in approximately 34% of cases, and it is present as a comorbidity in over 80% of patients in dialysis programs. This means that there is a major problem to be tackled, with a potential therapeutic inertia (TI) that could be responsible for worse morbidity and mortality outcomes^2^.

## Definition, Epidemiology, and Pathophysiology of Hypertension in Dialysis Patients

### Definition/Epidemiology

HTND is defined based on observations previously established for the general population, with thresholds for determining normotension or hypertension varying across different guidelines^3^. Measuring BP in dialysis patients thus poses a challenge to standardization. Ideally, BP should be based on home interdialytic assessments with HBPM or ABPM, following the recommended standardization^4,5^.

In epidemiological terms, HTN occurs in more than 80% of dialysis patients, and is often poorly controlled^6^.

Key messages:

- IDH has an estimated prevalence of 5% to 15% and is correlated with hospitalizations and mortality^7^ (Class I/Level B);
- Many studies show the presence of reverse epidemiology, with J or U-shaped curves^8^ (Class I/Level B).

For the diagnosis of HTN, it is recommended:

- HBPM: mean ≥ 130/80 mmHg, considering the day of installation and additional 6 consecutive days (see protocol in Chapter 3);
- ABPM in HD: mean ≥ 130/80 mmHg in 44h (see protocol in Chapter 3);
- ABPM in PD: mean ≥ 130/80 mmHg in 24h (see protocol in Chapter 3);
- HBPM/ABPM unavailable: measurements taken on a non-dialysis day, midweek for HD, or at the office for PD;
- IDH is defined as an increase ≥ 10 mmHg in SBP during or immediately after HD in 4 out of 6 sessions. Motivates further evaluation^7^.

Key messages:

- Pre-, intra- and post-HD BP measurements are inaccurate for diagnosis, but useful for diagnosing IDH and for hemodynamic control^9^ (Class I/Level B).
- The diagnostic threshold for pre-HD is > 140/90 mmHg, and post-HD > 130/80 mmHg^10^ (Class I/Level B).

### Pathophysiology of Htnd

**Figure 1:**
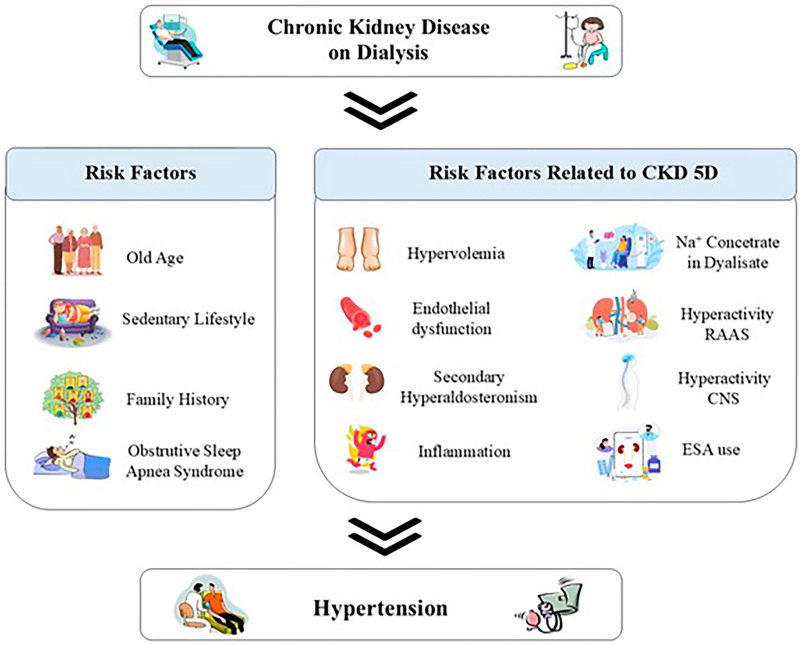
Pathophysiology of hypertension in patients undergoing dialysis treatment.

The pathophysiology of HTND is complex and multifactorial, encompassing both general and CKD-specific factors^5^ (Figure 1). However, most patients with CKD 5D have HTN and DM as their main causes. This means they represent a population with HTN that may precede the onset of dialysis therapy by decades. Additionally, patients with CKD of other etiologies also present with HTN, often secondary to the baseline kidney disease.

Abbreviations – CKD Grade 5D: chronic kidney disease stage 5 on dialysis; OSA: obstructive sleep apnea; Na+: sodium; RRT: renal replacement therapy; RAAS: renin-angiotensin-aldosterone system; SNS: sympathetic nervous system; EPO: recombinant human erythropoietin.

#### Relationship between volume overload and hypertension in CKD

The role of hypervolemia in HTND could be evidenced by the reduction in BP associated with intensified UF. In a study, daily, long-term HD resulted in BP control in about 90% of patients^11^, with a dissociation observed between achieving the estimated DW and BP control, verified weeks or months after reaching euvolemia. This phenomenon, known as the “lag phenomenon”, appears to be related to the additional removal of salt as UF is intensified^12^. Similar results have been observed in Turkey^13^. Common strategies in these studies included restricting salt in the diet and extended dialysis sessions. A randomized study, in which UF intensification was achieved without extending the HD session length, confirmed a significant improvement in BP control in patients who reached the estimated DW, compared to those in the control group^14^.

In PD patients, few studies have assessed the relationship between hypervolemia and HTN^15^. One of them evaluated PD patients undergoing intensified UF, with a 5% reduction in body weight^16^. After 5 weeks, a reduction in body weight, extracellular water, and inspiratory diameter of the inferior vena cava were observed, along with improved BP control. Hypervolemia appears to play a predominant role in HTN in PD patients.

In recent decades, a new element has been associated with excess sodium in CKD. In 2003, Titze et al.^17^ demonstrated the sodium retention complexed to glycosaminoglycans in the skin and muscles of animal models, therefore without water retention. Non-osmotic sodium accumulation creates a “buffer” system for sodium storage^18^, with macrophage recruitment, induction of the tonicity-responsive enhancer-binding protein (TonEPB) gene expression, and vascular endothelial growth factor C (VEGF-C), which induce hypertrophy of lymphatic vessels, NO release, and vasodilation. However, in situations of excess sodium or impaired sodium excretion, the functioning of this “buffer” system is compromised, leading to increased sodium in the skin and muscles, with immune system activation, inflammation, and fibrosis^19^. Increased subcutaneous sodium content, detectable by magnetic resonance imaging, contributes to HTN and increased CV events in chronic kidney patients^20,21^.

Key message:

- The role of hypervolemia in HTN in CKD stage 5D could be evidenced by the BP reduction associated with intensified UF (Class I/Level B). In PD patients, it also appears to play a leading role in HTN (Class I/Level C).

#### Parameters interfering with BP: sodium/composition of dialysis solution

Dialysis patients are typically hypervolemic and hypertensive, especially due to exacerbated salt sensitivity, inappropriate activation of the RAAS in response to sodium intake, and decreased natriuresis^22^. The body’s sodium pool includes skin and muscle deposits, particularly in diabetics^23^, with partial removal by HD^21^, and impacts on BP control and LVH^24^. When it persists at the end of HD, HTN may translate into residual hypervolemia^25^, as well as in the interdialytic interval^26^.

Different sodium concentrations in the dialysis solution may be related to volume and BP control^27,28,29,30,31^ and, if higher than serum concentrations, result in increased IDWG, without correlating with BP variations^27^. Patients subjected to lower sodium concentrations in the dialysis solution experienced a higher occurrence of intradialytic hypotension^27,28,29,31^, reduced IDWG^28,29,30,31^, lower BP^29,28,29,30,31^, and minimized use of antihypertensives^29^, but with no positive effects on the reduction of LVH^28^, hospitalization rates^27^, and mortality^27^. An ongoing study aims to evaluate the effect of different dialysate sodium concentrations on CV events and mortality in HD patients (RESOLVE, NCT02823821).

In addition, pre-HD natremia usually approaches the physiological level of individual adjustment (set point), and it is possible to adopt a dialysis solution sodium concentration similar to this level (isonatremic dialysis), based on sodium removal by convection and avoiding positive balance^32^.

Key messages:

- Caution is advised when increasing or decreasing the sodium of the dialysis solution, since high sodium levels increase the IDWG and, conversely, low sodium levels reduce the IDWG and BP, but increase the risk of intradialytic hypotension (Class I/Level B).
- There is no indication to adjust the sodium concentration in the dialysis solution with the aim of reducing hospitalization or mortality (Class I/Level B).

#### Ultrafiltration (UF) Rate

High UF rates are associated with hypotension, but if they are too low, they could perpetuate hypervolemia, the main determinant of HTN^6^. The relationship between UF and BP reduction is consistent, but in hypertensive patients it may be influenced by other factors involved in the pathophysiology of HTN^33^.

Essential to avoid hypotension, the maintenance of plasma volume during HD depends on refilling (absorption of interstitial and lymphatic fluids in the microcirculation), which *per se* is independent of whether or not higher UF rates are adopted^34^.

Counterregulatory compensatory mechanisms to UF are reflex tachycardia and vasoconstriction. Therefore, patients with autonomic and/or ventricular dysfunction may develop intradialytic hypotension even with low UF rates^35^. High UF rates are associated with intradialytic hypotension, reduced RRF, and increased mortality^35,36,37,38,39^. The adoption of UF rates > 13 mL/kg/h^35,36,37,38,39^ seemed to be deleterious overall, and UF rates > 10 mL/kg/h^36,37,39^ are harmful, especially in the presence of ventricular dysfunction^35^. In a HD regimen of 1 to 2 times a week, even lower UF rates (less than 6 mL/Kg/h) appear to be necessary^36^. If RRF remains preserved, the association between high UF rates and mortality is attenuated^36^.

Hypotension is associated with an accelerated reduction in RRF, mitigating its positive effect on mortality, both in HD and PD^40^. It is recommended to increase either the HD session length or its frequency to avoid UF rates > 13 mL/kg/h while still managing hypervolemia^9,41^. Essentially, these UF rate limits may be adopted, but a set of factors should also be considered, including intradialytic hemodynamics, comorbidities, symptoms, and medication use^41^.

Key messages:

- It is recommended to avoid high UF rates, i.e. > 13 mL/kg/h, as they are associated with increased mortality, intradialytic hypotension, and an accelerated reduction in RRF.
- It is recommended that in patients with ventricular dysfunction, the suggested threshold is even lower (up to 10 mL/kg/h) (Class I/Level B).

#### Renin-Angiotensin-Aldosterone System (RAAS) activation

The kidneys can synthesize all components of the RAAS, even in dialysis patients^42^, in whom PRA is inappropriately elevated in relation to hypervolemia. These data are reinforced by the increase in PRA following dialysis sessions, indicating that remaining nephrons may perceive sodium variations and increase RAAS activity. Angiotensin II exerts deleterious effects by stimulating aldosterone production, and sodium retention by causing endothelial damage and stimulating the SNS^42,43,44^. The administration of lisinopril to HD patients resulted in improved BP control, as assessed by 44-hour ABPM, when compared to the control group^45^.

In addition to Angiotensin II, the vasculotoxic action of aldosterone has been shown, especially in the presence of salt. This finding suggests that aldosterone may play a permissive role in sodium toxicity in the endothelium, thereby becoming a risk factor for CV complications^46^.

Accordingly, blockade of mineralocorticoid receptors with spironolactone resulted in improved BP control and reduced CV mortality in HD patients^47^. Thus, aldosterone contributes to the worsening of HTN by exerting genomic and non-genomic effects, inducing inflammation and vascular toxicity in the presence of excess salt.

Key message:

- It is recommended to block RAAS due to its inadequate activation, despite hypervolemia, which significantly contributes to the genesis of HTN and to increased CV risk in CKD patients (Class I/Level B).

#### Sympathetic Nervous System (SNS) activation

Activation of the SNS in CKD is one of the main mechanisms related to the pathophysiology of HTN. Factors responsible for SNS hyperactivity in CKD include reduced bioavailability of NO, ED, uremic toxicity, and inflammation, as well as a high prevalence of OSA^48,49,50^.

Increased plasma norepinephrine concentrations and sympathetic activity, assessed through muscle sympathetic nerve activity (MSNA), highlight the importance of the SNS in the pathophysiology of HTN in CKD. Renal sympathetic afferent nerves innervating the renal arteries, and CNS efferents stimulated by B1 receptors, cause renal arteriolar vasoconstriction and RAAS activation, with a consequent increase in renal vascular resistance and sodium retention^51^. CKD patients exhibit renalase deficiency, an enzyme produced by the kidneys and responsible for metabolizing catecholamines^52^.

SNS hyperactivity also contributes to CV mortality, being associated with atherosclerotic disease, LVH, and the presence of cardiac arrhythmias, which account for approximately 25% of deaths in dialysis patients^53^. Sympathetic hyperactivity is confirmed by improved BP control following native kidney nephrectomy in patients with CKD stage 5D^54^, and by renal denervation in patients at different CKD stages^55^ and on HD^56^. Finally, the administration of BB in dialysis patients reduces CKD progression and mortality^57,58^.

Key message:

- SNS blockade is recommended for the treatment of HTN, as sympathetic hyperactivity is an important pathophysiological mechanism in HTN and mortality in CKD (Class I/Level A).

#### Endothelial dysfunction

It is reasonable to state that endothelial disfunction associated with HTN in patients on HD or PD precedes the diagnosis of CKD by decades, considering that most CKD patients have DM and/or HTN, or are elderly and present several CV risk factors. A study in rats subjected to 5/6 nephrectomy documented the reduction in endothelial NO synthase activity, resulting in lower NO availability and elevated BP^59^. The reduction in NO supply has been attributed to changes in the metabolism of pteridines, which are aromatic compounds that act as cofactors in various inflammatory and immunological processes^60^. In patients with stage 5D CKD, the reduction in the BH4/BH2 ratio - compounds belonging to the pteridine group - was associated with a reduction in endothelial NO availability, inflammation/ malnutrition processes, and CVD^61^. Also, the oxidative stress present in CKD^62^, and particularly the increased plasma levels of asymmetric dimethylarginine (ADMA), interfere with NO production and are associated with LVH and CV mortality in HD patients^63,64,65^. ADMA is an endogenous inhibitor of NO synthesis, which accumulates due to reductions in GFR and intracellular metabolization^65^.

Patients with stage 5D CKD have elevated ET1 levels, which contribute to the worsening of HTN^66^ and play a significant role in the occurrence of IDH^67^.

Key message:

- Main message: The pre-existing endothelial dysfunction experienced by most CKD patients becomes even more severe as GFR decreases, thus contributing to the pathophysiology of HTN and the occurrence of CV events and death (Class II/Level B).

#### Increased Arterial Stiffness (AS)

AS causes an increase in peripheral and central BP, such as SBP, PP, and LV mass, as well as reductions in DBP and coronary perfusion. AS may also be considered an independent factor for CV mortality and CKD progression.

The mechanisms involved in AS in patients with CKD are not yet fully defined, but they include arterial calcification, chronic volume overload, mechanical stress due to HTN, chronic microinflammation, sympathetic and RAAS hyperactivity, accumulation of glucose degradation products, lipid peroxidation, and abnormalities in the NO system. Even a mild reduction in GFR is a risk factor for the development of AS. UF in HD is not capable of significantly reversing/decreasing AS^68,69,70,71^.

AS is multifactorial and some of these are exclusive to CKD: a) high phosphorus levels, which activate genes related to the osteoblast phenotype in smooth muscle cells, leading to arterial calcification^72^; b) protein-energy malnutrition, which is common in CKD and causes an increase in AS^73^; c) elevated ADMA; d) increased FGF 23; e) reduced magnesium^74,75,76^. Low serum concentrations of fetuin-A are associated with vascular calcification in CKD^77^.

#### Obstructive Sleep Apnea (OSA) in HTND

OSA is defined as a partial or complete collapse of the upper airways during sleep, causing sleep fragmentation and intermittent hypoxemia. The diagnosis and severity of OSA are based on the apnea-hypopnea index (AHI) obtained through polysomnography or polygraphy: up to 4.9 = no OSA; 5.0 to 14.9 = mild OSA; 15 to 29.9 = moderate OSA; and ≥ 30 events per hour = severe OSA^78^.

OSA is highly prevalent in the dialysis population, affecting 50% to 70% of patients. When associated with HTN, it exhibits pathophysiological mechanisms that are amplified, most notably hypervolemia^79,80^.

The clinical picture is poorly specific, with a lower prevalence of snoring and daytime sleepiness reported in CKD 5D^81^. Indication for an objective sleep examination should be considered more liberally in these patients. Severe OSA may be related to resistant and refractory HTN^82^.

Once moderate or severe OSA has been identified, regardless of BP behavior, treatment should be individualized. The indication for continuous positive airway pressure (CPAP) during sleep may not be the first choice in dialysis patients. To reduce hypervolemia in this subgroup, UF optimization techniques, such as extended nocturnal HD, or PD with cycler machines, have proven effective in small clinical trials^78^.

Key message:

- The clinical picture of OSA in patients with CKD 5D is poorly specific. The indication for an objective sleep examination (polysomnography or polygraphy) should be considered in a more liberal and individualized manner, as should the use of CPAP (Class IIB/Level C).

#### Drugs that interfere with blood pressure control

Increased BP is a well-recognized complication of EPO therapy in HD patients^83^. Approximately 30% of patients develop HTN or require adjustment of antihypertensive medication after a few weeks or months. The pathophysiology of HTN due to EPO use appears to be independent of its effect on red blood cell mass and viscosity^84^. The most likely mechanisms involve increased production or enhanced response to ET-1, a pronounced increase in BP response to angiotensin II infusion, and hypersensitivity to norepinephrine^83^. There are reports of an association between abnormalities in the circadian rhythm of BP and the use of EPO in ABPM^3^. Preventing EPO-induced hypertension is a clinical challenge with several possible management strategies listed below as recommendations^83,84^.

Finally, the use of drugs that notoriously raise BP should be avoided, which are already extensively mentioned in the Brazilian Hypertension Guidelines^4^.

Key messages:

In EPO-induced HTN refractory to antihypertensive management, the following are recommended:

- Attention to dry weight (Class IIA/Level B);
- Preference for subcutaneous route of EPO administration (Class IIA/Level B);
- Reduce hemoglobin target (Class IIA/Level C);
- Start with a low dose of EPO, increase slowly and, in extreme cases, discontinue its use (Class IIA/Level B);
- Avoid the use of drugs that notoriously raise BP (Class IIA/Level B).

#### Inertia of the care team

Clinical or therapeutic inertia (TI) refers to the failure of healthcare professionals to initiate, intensify, or discontinue treatments when indicated, including both pharmacological and non-pharmacological measures^85^. TI is observed in around 2/3 of visits with hypertensive patients^86^. Also, in the dialysis literature, there is evidence associated with failure in introducing or intensifying antihypertensive therapy or adjusting volume status. In a retrospective analysis of patients with CKD and uncontrolled HTN, TI appears to have occurred on approximately 44% of occasions, judging by the non-identification of a reason for the failure in medical decision-making^87^.

Regarding the psychological profile of the physician most prone to TI, the preference for apparent short-term safety through inaction seems to predominate^88^. Coping with this behavior involves the continued encouragement of proactive conduct aimed at achieving therapeutic goals, within a policy of improving quality of care^89^.

TI is described in various CV prevention scenarios^89^, such as the management of dialysis patients with DM^90^ or elderly patients on polypharmacy^91^. Implicating factors include clinical uncertainty regarding BP measurement, poor medication adherence, and diastolic and/or orthostatic hypotension^87^.

Examining the use of potentially inappropriate medications (centrally-acting alpha-agonists or alpha-blockers), the risk of TI in elderly dialysis patients was higher among black individuals, with polypharmacy, and without functional limitations. However, there was no increase in hospitalizations or mortality in those who maintained these medications^87^.

Among hypertensive patients, the use of structured medical education and a regular feedback system was successful, with greater control of HTN, but no improvement in CV outcomes^92^.

Recommendations in the existing literature indicate that patient education and involvement in the management of their CV risks reduce TI^88,93^.

Key message:

- To reduce therapeutic inertia, it is recommended that protocols should involve patients, caregivers, physicians, and a multidisciplinary team for achieving clinical goals in the control of HTN (Class I/Level B).

#### Poor adherence to antihypertensive treatment

Adherence can be defined as the extent to which an individual complies with the recommendations of a healthcare provider^94^. Low adherence to treatment is associated with unfavorable clinical outcomes and is common among patients with CKD 5D^95^.

Regarding BP control in HD or PD, poor adherence could pose a series of difficulties for the management of HTN. The lack of implementation of behavioral measures, such as reducing salt intake and limiting weight gain in the interdialytic period, coupled with a failure to use prescribed antihypertensive drugs - whether due to complex dosing regimens, associated side effects, or, in the specific case of HD patients, the fear of intradialytic hypotension episodes - could hinder the achievement of therapeutic goals.

As for strategies to improve adherence, studies in this population are scarce. In general, the following strategies are recommended:

1. Reduction in salt intake/limitation of interdialytic weight gain (IDWG): structured interventions based on continuous patient education, conducted by a multidisciplinary team, may improve these variables^96^.
2. Measurement of BP in the interdialytic period, through HBPM, promotes greater adherence to antihypertensive drugs, directly impacting on BP control^97^.
3. Pharmacological treatment: selection of medications with a lower adverse event profile and better dosing convenience^4^.
4. In HD patients, the medication schedule should be adjusted according to the session, without discontinuing antihypertensive drugs, particularly in patients with a tendency to intradialytic hypertension^98^.

Key messages:

Strategies to improve adherence to antihypertensive treatment in HD patients include:

- Continuous education aimed at limiting salt intake and IDWG (Class IIA/Level B).
- Home or outpatient BP measurement during the interdialytic period (Class IIA/Level B).
- Use of antihypertensive drugs, preferably in a single daily dose (Class I/Level A).

## Diagnosis of Hypertension in Patients Undergoing Peritoneal Dialysis (PD) and Hemodialysis (HD)

BP measurements related to the HD session are not sufficient for diagnosing HTN and have poor prognostic value. Observational studies report a U-shaped association between peridialytic BP and mortality. In contrast, BP outside the dialysis unit shows a linear and direct association with mortality^99,100^. The BP behavior in dialysis patients is directly related to their volume status, so that measurements taken prior to HD overestimate BP, while those taken after HD underestimate it^101^. As opposed to this expected drop in BP during HD, 7–30% of these patients experience an increase during this period^102^. This contributes to the variability and inconsistency in BP measurements during the peridialysis period. Conversely, there is growing evidence of the superiority of measurements taken outside the HD unit.

### Blood Pressure Measurement Related to the HD Session

BP measurement related to the HD session can be assessed using three measures: predialysis BP, intradialytic BP, and postdialysis BP. Determining the number of readings to be taken during the intradialytic period will depend on the nephrologist’s clinical judgment. This decision will consider pre-dialysis BP values, a history of intradialytic hypotension or hypertension episodes, the need for elevated ultrafiltration rates, patients undergoing dry weight adjustment, and the patient’s general clinical condition.

Key messages:

- BP during the HD session has low prognostic value (Class I/Level B).
- It is recommended that during the HD session, BP be measured at least every hour (Class IIA/Level C).
- According to the Kidney Disease Outcomes Quality Initiative (KDOQI), in HD patients, HTN should be diagnosed as pre-dialysis BP > 140/90 mmHg, or post-dialysis BP > 130/80 mmHg10 (Class IIA/Level B).

However, they are taken under circumstances that deviate from those recommended for adequate BP measurement, including: anxiety about starting the treatment, anticipation of pain from the arteriovenous fistula puncture^103^, discontinuation of antihypertensive drugs on the day of HD (in some patients), clearance of antihypertensive drugs, use of erythropoiesis-stimulating agents, white coat and masking effects, pre- or intradialytic diet, validation of oscillometric devices used in HD machines, and a high number of patients being assessed by the same health professional in a short period of time.

Several studies have been conducted to assess the diagnostic accuracy of peridialytic BP measures, as well as their representativeness of BP in the interdialytic period^101,104,105^. The pattern observed in most patients is a tendency towards a reduction in post-dialysis BP in relation to pre-dialysis BP. This can be explained by the hemodynamic response to UF during dialysis, and generally, the magnitude of this reduction is associated with the ultrafiltrate volume. Similarly, studies suggest that IDWG has a direct influence on the elevation of pre-dialysis BP^105^. Performing the correct BP measurement technique in the HD unit is challenging, and for this reason, inadequate technique has already been implicated in the poor performance of pre- and post-HD BP to diagnose and/or assess the prognosis of HTN in these patients. However, a study has demonstrated that, even when properly performed, this type of measure has no prognostic significance^104^. Certain conditions are inherent to the dialysis setting, and performing pre- and post-dialysis BP measurements in a designated location, that preserves the necessary criteria for correct BP measurement, may not be feasible within the logistics of a HD session. Thus, BP measurements during the HD session should not be used for diagnosing HTN or defining adjustments to the patient’s antihypertensive regimen^101^.

Peridialytic BP measurements are imprecise estimates of BP, limiting their ability to provide clear and direct prognostic associations, even when such an association does exist.

### Intradialytic Hypertension (IDH)

Despite fluid removal through UF, there is a subset of patients who exhibit a rise in BP during and/or after the HD session, compared to pre-HD BP levels. These patients are classified as having IDH^5^. An increase in SBP ≥ 10 mmHg during or after the HD session compared to pre-dialysis levels in 4 out of 6 sessions is commonly used to characterize a patient with IDH^106^. However, Singh et al. reported that any observed increases in SBP are related to a higher risk of fatal CV events^107^.

It is important to note that IDH is not detected in all HD sessions for the same patient. Conversely, there is a greater correlation between BP values detected within the first 90 minutes of HD and those measured immediately after the session. In Brazil, a single-center analysis showed that 11% of patients experienced an increase in SBP ≥ 10 mmHg in more than 50% of HD sessions^108^. The pathophysiological mechanisms that justify this condition include hypervolemia, positive sodium balance, RAAS and SNS hyperactivity, endothelial disfunction, and higher or lower clearance of antihypertensive medications^109^.

The main mechanisms involved in intradialytic HTN are highlighted in Chart 1.

**Chart 1:** Pathophysiological mechanisms of intradialytic hypertension

Hypervolemia	Excess fluid is one of the primary causes of elevated blood pressure in patients with intradialytic hypertension (IDH). The paradoxical rise in blood pressure observed in some patients during ultrafiltration may result from increased cardiac output due to hypervolemia and dilation of the cardiac chambers. This indicates that intensifying ultrafiltration and reducing dry weight could serve as effective treatment strategies for IDH.
Activation of the renin-angiotensin-aldosterone system (RAAS)	RAAS stimulation is triggered by hypovolemia resulting from ultrafiltration.
Sympathetic System Hyperactivity	Individuals with chronic kidney disease (CKD) typically exhibit sympathetic hyperactivity, which can be alleviated by increasing the frequency of hemodialysis sessions^110^.
Sodium Control	A positive sodium balance is one of the primary mechanisms contributing to extracellular fluid overload and hypertension in dialysis patients. Effective sodium removal can be achieved by adjusting the ultrafiltration rate and the sodium concentration in the dialysate.
Endothelial Dysfunction	In response to ultrafiltration and both mechanical and hormonal stimuli, endothelial cells synthesize and release humoral factors that play a role in blood pressure homeostasis. Patients with intradialytic hypertension (IDH) exhibit a significant increase in plasma endothelin-1 levels and a decrease in nitric oxide levels compared to controls^109^.
Dialysis clearance of antihypertensive medications	Antihypertensive medications vary in their susceptibility to filtration through the dialyzer membrane.

Abbreviations – IDH: intradialytic hypertension; CO: cardiac output; UF: ultrafiltration; DW: dry weight; RAAS: renin angiotensin aldosterone system; CKD: chronic kidney disease; NO: nitric oxide.

Several strategies have been proposed for the treatment of IDH, such as^111^:

- Optimization of antihypertensive treatment: ensuring the use of appropriate medication, aiming for the ideal dry weight, checking adherence to both pharmacological and non-pharmacological treatment, in addition to extending the duration and/or frequency of HD sessions;
- Consider the use of BB with alpha-blocker activity (labetalol);
- Use short-acting antihypertensives before starting the HD session;
- Selection of less dialyzable antihypertensive agents;
- Consider reducing sodium concentration of dialysis solution;
- Avoid a very high calcium concentration in the dialysis solution;
- Consider elevating the temperature of the dialysate. Very cold baths are favorable to the emergence of IDH;
- Consider administering erythropoiesis-stimu­lating agents subcutaneously.

### Blood Pressure Measurements Outside the Hemodialysis Unit

These measurements have proven to be a more accurate parameter for assessing BP changes related to reduced DW, in addition to being more associated with target organ damage and CV events. In these patients, the prevalence of nocturnal HTN (risers) and the non-dipper pattern are usually higher than those in the overall hypertensive population^112,113^.

#### Self-measurement of blood pressure in dialysis patients

There is no conclusive evidence validating specific protocols (number of measurements and timing) nor normality values for this method^114^. However, SMBP should follow the same recommendations and precautions as office BP measurements.

Key message:

- SMBP is not recommended on a routine basis for the diagnosis and management of HTN in HD or PD patients and should only be used as a screening method (Class I, Level C).

#### Ambulatory Blood Pressure Monitoring (ABPM) and Home Blood Pressure Monitoring (HBPM) 

In HD patients, ABPM and HBPM have shown a better correlation with target organ damage, CV events, and death from any cause^100,101,104^. Both display a good correlation with each other, as reported in the DRIP study, in which BP changes assessed by HBPM after 4 and 8 weeks of DW adjustment were associated with BP changes assessed by 44-hour ABPM^105^. Another benefit demonstrated in a study with HD patients was the finding that an antihypertensive medication adjustment strategy based on HBPM proved to be more effective in BP control than a strategy based on pre-HD SBP^97^.

Performing a 44-hour ABPM, fitting the device after a midweek dialysis session (between the second and third sessions of the week) and removing it immediately before the next session, provides prognostic information in HD patients and is the only method capable of assessing BP behavior during sleep. As in the general population, the sleep-wake ratio is a significant predictor of clinical outcomes in dialysis patients. Therefore, the absence of BP dipping during sleep (< 10% of the average nighttime BP in relation to wakefulness) or reverse dipping (elevated nighttime BP in relation to wakefulness), is associated with the risk of overall and CV mortality^115,116^. However, ABPM is not a practical test to perform and has low acceptance in this population, partly due to the prevalent frequency of sleep disorders and pruritus associated with CKD^78,117^. Conversely, HBPM, in addition to showing a good correlation with prognosis, is low-cost and better tolerated^118^. The advantages of the methods are listed in Chart 2, and the recommended protocols for their implementation are shown in Charts 3 and 4 (Class IIA/Level C).

**Chart 2:** Advantages of ambulatory blood pressure monitoring (ABPM) and home blood pressure monitoring (HBPM)

Advantages common to ABPM and HBPM	
• Characterization of white-coat hypertension	
• Characterization of masked hypertension	
• Identification of true resistant hypertension	
• Greater adherence to the diagnosis and treatment of hypertension	
• Better efficiency in blood pressure control	
• Good reproducibility	
• Better correlation with target organ damage and cardiovascular events than blood pressure measured at dialysis unit	
**Advantages of ABPM**	**Advantages of HBPM**
• Regarded as the gold standard for blood pressure assessment.	• Assessment of the highest number of blood pressure measurements over the greatest number of days.
• Assessment of blood pressure during sleep and daily activities.	• Involves the patient in their self-care through blood pressure measurement.
• Assessment of rapid morning blood pressure elevation.	• Low-cost.
• Evaluates 24-hour blood pressure management.	• Well accepted by patients.

Abbreviations – ABPM: ambulatory blood pressure monitoring; HBPM: home blood pressure monitoring.

**Chart 3:** Protocol for conducting ambulatory blood pressure monitoring (ABPM) in dialysis patients

ABPM
In patients undergoing hemodialysis three times a week, 24-hour ambulatory ABPM does not capture blood pressure measurements throughout the interdialytic cycle. For these patients, a 44-hour ABPM is recommended^5^. It is important to note that the cuff should not be placed on the arm with the arteriovenous fistula (AVF).
**Verification period:**
• 44 hours (if the software does not support the 44-hour protocol, two consecutive 22-hour assessments are recommended). For patients undergoing daily hemodialysis (HD) or peritoneal dialysis (PD), a 24-hour ABPM is recommended.
**Number of measurements:**
• At least 48 valid measurements are required during the 44-hour ABPM (32 while awake and 16 during sleep) and 24 valid measurements during the 24-hour ABPM (16 while awake and 8 during sleep).
**Installation day:**
• The device should be installed after the mid-week dialysis session and removed immediately after the following session. For a 44-hour ABPM, it should be removed just before the dialysis session.
**ABPM report:** The report must include the date and time of the start and end of the assessment (indicating the interdialytic period during which it was conducted), the number and percentage of measurements taken versus valid measurements, the average systolic blood pressure (SBP) and diastolic blood pressure (DBP) over 24 hours during wakefulness and during sleep. It should also detail blood pressure behavior during wakefulness and sleep, episodes of hypotension and/or blood pressure peaks, and any correlations between activities, symptoms, and medications. The conclusion should specify whether the blood pressure behavior was considered normal or abnormal (abnormal if the mean SBP ≥ 130 mmHg and/or DBP ≥ 80 mmHg, as shown in Table 1).

Abbreviations – ABPM: ambulatory blood pressure monitoring; HD: hemodialysis; BP: blood pressure; h: hours; AVF: arteriovenous fistula; HBPM: home blood pressure monitoring; SBP: systolic blood pressure; DBP: diastolic blood pressure.

**Table 1:** Blood pressure values considered abnormal for ambulatory blood pressure monitoring (ABPM) and for home blood pressure monitoring (HBPM)

	SBP (mmHg)			DBP (mmHg)	
ABPM 44h	≥130		and/or	≥80	
ABPM awake	≥135		and/or	≥85	
ABPM sleep	≥120		and/or	≥70	
HBPM	≥130		and/or	≥80	

Abbreviations – ABPM: arterial blood pressure monitoring; HBPM: home blood pressure monitoring; SBP: systolic blood pressure; DBP: diastolic blood pressure.

**Chart 4:** Protocol for home blood pressure monitoring in dialysis patients according to the Brazilian guidelines for blood pressure measurements in- and out-office 2023^119^

Home Blood Pressure Monitoring - HBPM
**Verification period:** Day of installation + six consecutive days.
**Number of measurements:** Ideally, 36 measurements (at least 18 valid measurements), taken every day, covering both morning and evening/night periods.
**Day 0 or installation day:** For HD and PD, measurements are taken in the office or in the HD clinic, never on the arm with the AVF. Ideally, three measurements are taken (using the average of the last two for the calculation of white-coat and masked blood pressure effects) and measurements are taken at home at night. Measurements taken on the installation day, either in the office or at home, should be excluded from the calculation of the HBPM average.
**HBPM days:** At home, BP measurements are taken for six more days. The patients should take three measurements in the morning and three in the evening or at night. For patients undergoing conventional HD (two or three times weekly), measurements taken on dialysis days should be excluded from the calculation of the HBPM average. In cases of daily HD and PD, all measurements are included in the average calculation.
**HBPM report:** It should include the reason for the examination request, the number of days of effective measurements, the time and number of measurements on each day, the quality of the procedure, mean BP (total, daily, and for morning and evening/night periods), whether white-coat or masked blood pressure effects were present, and whether the results were considered normal or abnormal (abnormal if averages are ≥ 130 mmHg and/or ≥ 80 mmHg).

Abbreviations – HBPM: home blood pressure monitoring; HD: hemodialysis; PD: peritoneal dialysis; BP: blood pressure; AVF: arteriovenous fistula.

Key messages:

- Message: Both ABPM and HBPM are preferred over office BP in terms of predicting clinical outcomes with a better correlation with target organ damage, CV events, and death from any cause when compared to peridialytic measures (Class I/Level B).
- Both ABPM and HBPM show good correlation with each other in HD and PD (Class I/Level B).
- 44-hour ABPM is recommended as the gold standard method for diagnosing HTN in HD and PD patients^113^ (Class I, Level B).
- HBPM (installation day and 6 additional days) is recommended as an alternative when ABPM is unavailable (Class I, Level C).
- For PD and daily HD, it is recommended to follow the guidelines for the hypertensive population in general, regarding HBPM and 24-hour ABPM (Class I/Level C).

Regarding peritoneal dialysis (PD), as it is a home dialysis modality, it is suggested that HBPM should be widely applied. In its latest update, the International Society for Peritoneal Dialysis recommends that, in addition to BP measurement during clinical visits, home BP should also be measured on a weekly basis^120^. However, due to the scarcity of evidence, prospective studies are needed to elucidate the actual prognostic significance of out-of-office BP measurements in PD.

#### Phenotypes

Considering both in-office and out-of-office BP, there are 9 possible types of BP behavior: True Normotension (TN), Controlled Hypertension (CH), Sustained Hypertension (SH), Sustained Uncontrolled Hypertension (SUH), White Coat Hypertension (WCH), White Coat Uncontrolled Hypertension (WCHNC), Masked Hypertension (MH), Masked Uncontrolled Hypertension (MUH), and Nocturnal Hypertension (NH).

**Figure 2:**
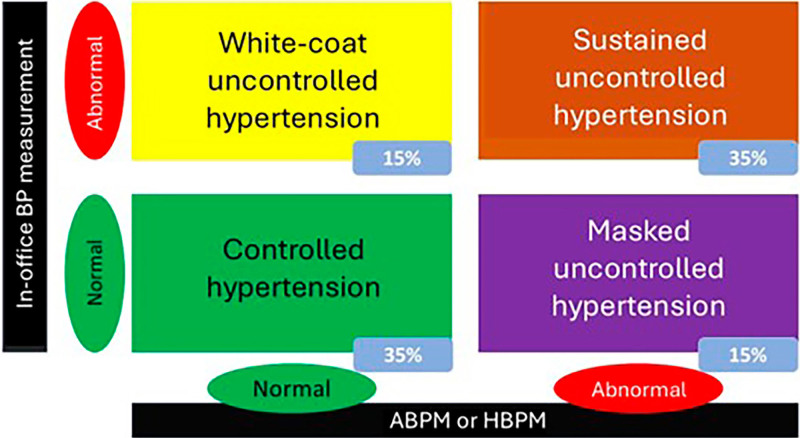
Prevalence of hypertension phenotypes in patients with chronic kidney disease on hemodialysis.

WCH refers to a condition in which BP is abnormal in the office but normal when measured by ABPM or HBPM. MH refers to patients whose BP is normal in the office but abnormal when measured by HBPM or ABPM. In patients taking antihypertensive medication, the term “uncontrolled” should be added. TN is when both in- and out-of-office BP measurements are normal and the patient is not on antihypertensive medication; if they are, it is defined as CH. SH is used when both are abnormal in the absence of antihypertensive drugs. If the patient is taking antihypertensive medication, it is defined as SUH. NH is defined when the average BP measured by ABPM is altered during sleep at 24h or 44h, but normal during wakefulness. The prevalence of phenotypes varies considerably according to the BP measurement method and the definition of HTN. In one of the few studies assessing the prevalence of phenotypes among HD patients, 44-hour ABPM abnormal values ≥ 135/85 mmHg and ≥ 140/80 mmHg were used for the median pre-HD and post-HD BP of six consecutive midweek sessions. The prevalence rates found can be seen in Figure 2 ^121^.

Abbreviations – BP: blood pressure; HBPM: home blood pressure monitoring; ABPM: ambulatory blood pressure monitoring.

There is a lack of studies assessing the prognosis of phenotypes in dialysis patients. However, among patients with non-dialysis CKD, the risk of CV morbidity and mortality was not increased in those with WCUH, but it was increased in those with MUH and SUH^122^.

Key message:

- Message: Further studies are needed to assess HTN phenotypes in the population across all dialysis modalities.

## Complementary Assessments of Hypertension and Cardiovascular Risk in Peritoneal Dialysis (PD) and Hemodialysis (HD)

### Calculating Cardiovascular Risk in Chronic Kidney Disease on Dialysis

While CKD is a strong independent predictor of CV disease^123,124,125^, CV disease itself is the leading cause of death in chronic nephropathy patients, highlighting the importance of identifying patients at high risk of CV events and death^126,127^. In the general population, there are methods capable of predicting the 10-year probability of a CV event, notably the Framingham score and the Atherosclerotic Cardiovascular Disease (ASCVD) risk algorithm, which only consider traditional CV risk factors^128,129^. In CKD patients, these scores are less accurate, likely due to the existence of non-traditional risk factors related to loss of kidney function and dialysis treatment^130–133^. Consistent with this view, traditional risk factors are predominant in the early stages of CKD, while the non-traditional ones become more relevant as CKD progresses^130^.

Risk calculation models valuing specific factors for dialysis patients have been proposed^131,132,133^. From the analysis of HD patients, assessing the 5-year risk of CV events and death, a predictive score was developed including seven characteristics (age, sex, DM, previous CV disease, type of vascular access at dialysis initiation, monocyte/lymphocyte ratio, and uric acid), resulting in better performance than the Framingham score^10^. Evaluating Japanese patients on HD (J-DOPPS) over the course of a year, a model was developed including six variables (age, DM, previous CV disease, length of dialysis session, phosphorus, and albumin), also showing better risk discrimination than the Framingham score^132^. A European study of HD patients, followed for up to 2 years and including clinical and laboratory data (age, BMI, smoking, previous CV disease, CKD etiology, pre-dialysis BP, UF, hemoglobin, CRP, albumin, creatinine, and calcium), demonstrated good prediction of overall mortality risk^133^. Altogether, these studies show that adding non-traditional risk factors to predictive models has improved their accuracy in dialysis patients, although the observed death rate remains higher than the predicted one, suggesting that additional risk factors could be included in the construction of a more definitive model^131,132,133^. In any case, there is no widely accepted tool available for predicting CV risk in dialysis patients that is both simple and accurate.

Key messages:

- CKD patients should be considered at high CV risk (Class I/Level A).
- CV risk assessment of CKD patients may be conducted using risk calculation models for the general population (Class IIA/Level B).
- The accuracy of CV risk discrimination in CKD patients is enhanced using specific models that include both traditional and non-traditional risk factors related to CKD and dialysis (Class IIA/Level B).

### Natriuretic Peptides in Chronic Kidney Disease Patients

Serum natriuretic peptide levels are commonly used in clinical practice for the diagnosis and follow-up of patients with HF^134^. The diagnosis of HF is corroborated by at least one of the following factors: elevations in serum BNP or NT-proBNP concentrations, or objective evidence of pulmonary or systemic congestion of cardiogenic origin^134^.

These peptides, especially NT-proBNP, appear to predict the risk of CV outcomes and death in individuals at high CV risk^135,136,137,138,139,140,141^, including the potential for using NT-proBNP as an isolated variable that resembles or even surpasses the predictions of multivariate risk models^136,137,138,139,140,142^.

Chart 5 illustrates some studies demonstrating the association of NT-proBNP with increased CV risk in the CKD population^139,141,143,144,145,147^.

**Chart 5:** Studies in patients with kidney disease evaluating natriuretic peptides as biomarkers of risk of diseases, events, and cardiovascular death

Study	Study design	Kidney disease/other comorbidities	Results
TREAT^144^	Evaluation of the insertion of NT-proBNP and troponin T to a multivariable prediction model	CKD, with T2DM and anemia	Increased the predictive ability of CV outcomes by 17.8%
ALTITUDE^137^	Post-hoc analysis of NT-proBNP prediction (single variable) compared to a model with 20 variables	CKD and/or CV disease	Predictor of risk of CV events and death, similar to the multivariate model
CREATE^145^	NT-proBNP (single variable)	CKD (GFR 15-35ml/min) and anemia	Elevated plasma level as a predictor of risk of CV events and progression of CKD
Harrison TG et al., 2020^141^	Meta-analysis with 49 studies, evaluated BNP and NT-proBNP as a marker of risk of CV events and death	CKD 5D	Elevated levels of NT-proBNP and BNP were predictors of CV mortality and all-cause death
Satoh A et al., 2021^147^	NT-proBNP with measurement before the first HD of the week	Patients on HD	High serum levels were predictors of CV and overall risk of death

Abbreviations – DM2: Type 2 diabetes mellitus, CV: cardiovascular, CKD: chronic kidney disease, GFR: Glomerular filtration rate by creatinine clearance, HD: hemodialysis.

In dialysis patients, NT-proBNP appears to independently predict CV and all-cause mortality^141,147^, in addition to being a predictive factor for volume overload in HD patients (with or without a decline in LV ejection fraction)^147,148,149^ and for an increased risk of stroke-related hospitalizations^150^.

Since renal dysfunction is accompanied by increased concentrations of BNP and NT-proBNP^141,151^, the strategy of sequential measurements is superior to a single measurement in CKD patients^141^. Thus, in the absence of defined BNP and NT-proBNP cut-off values for dialysis patients, sequential values may better and more dynamically reflect the trajectory of cardiac function and hydration status.

Key message:

- It is recommended to perform serial measure­ments of natriuretic peptides in dialysis patients (preferably NT proBNP), if available, as they may help in the assessment of volume overload, identification and control of HF associated with dialysis CKD, and especially in predicting the risk of CV outcomes and death (Class I/Level B).

### Arterial Stiffness (AS) Assessed by Pulse Wave Velocity (PWV) Measurement

The degree of AS is a marker of vascular health and aging. One way of estimating it is by measuring arterial PWV, defined as the time taken (m/s) for the pulse wave (generated by systole) to travel between two sites in the arterial system, usually the carotid and femoral arteries. The carotid-femoral (aortic) PWV reflects the viscoelastic properties of the aorta and is considered the gold standard method for assessing AS^152^.

Elevated aortic PWV indicates increased AS and is associated with a higher risk of CV disease and mortality^152^. Normal values for aortic PWV vary according to age and sex (Table 2)^153^.

**Table 2:** Normal pulse wave velocity (PWV) values for men and women according to age group

Age group	PWV (m/s)	
	Men	Women
20–29 years	6.4	6.0
30–39 years	6.9	6.4
40–49 years	7.4	7.0
50–59 years	8.0	7.7
> 60 years	9.2	8.6

In HD patients, a greater aortic PWV value predicts higher mortality^154^, and PWV > 10 m/s is an independent predictor of both CV and all-cause mortality^155^. Similarly, accelerated PWV progression (measured at time 0 and every 6 months) may predict CV mortality in patients undergoing HD^156^.

HD patients appear to have PWV levels that are less sensitive to BP control, reflecting the overlap between traditional CV risk factors and non-traditional factors (related to uremia and dialysis treatment) that are determinants of the greater AS which characterizes this population. Altogether, this may justify the recognition that the lower variation in PWV observed regarding the management of HTN in these individuals was associated with a higher risk of CV events^157^.

Increased arterial stiffness is relatively common in PD patients. Higher PWV in PD patients is associated with increasing age, the presence of DM and hyperhydration, and has been shown to be a predictor of outcome in these patients^158^.

In conclusion, the measurement of PWV, considered the gold standard tool for AS, helps calculate CV risk, although further research is needed to establish definitive standards of PWV normality and its role in outcomes within the dialysis population.

Key message:

- Assessment of arterial stiffness using carotid-femoral pulse wave velocity (PWV) is recommended, if available, and on a serial basis, as it potentially predicts CV events as well as mortality in HD and PD (Class I/Level B).

### Bioimpedance as a Diagnostic Method for Body Composition and Volume Status

Bioimpedance analysis (BIA) assesses body composition by passing an electrical current of different frequencies through the body. It is a diagnostic method used in the context of dialysis patients to estimate the level of hydration and support in BP management^159,160,161^.

In a systematic review with meta-analysis of chronic HD and PD patients, Tabinor et al. suggest that mortality is predicted by a hyperhydration index > 15%, and a phase angle reduced by 1 degree, regardless of comorbidities^162^. In the largest study included, Zoccali et al. demonstrated higher mortality in hyperhydrated patients, regardless of pre-dialysis BP^163^. Other systematic reviews and meta-analyses have focused on showing the impact of using BIA in dialysis patients, and these are summarized in Chart 6 ^161,164,165,166,167^.

**Chart 6:** Summary of the main studies demonstrating the impact on the use of bioimpedance in dialysis patients

Studies	Study design	Improved control of hyperhydration	Improved control of BP	Other outcomes	Observations
Scotland et al., 2018^161^	Systematic review	**yes**	no	I: no M: no	BIA showed no effect on arterial stiffness and was not cost effective
Covic et al., 2017^164^	Systematic review and meta-analysis	**yes**	**yes**	M: no	No change in body composition
Beaubien- Souligny et al., 2020^168^	Systematic review and meta-analysis of randomized studies	**yes**	**yes**	LVH: no ECV: no **I: yes** M: no	-
Yang K et al., 2023^166^	Meta-analysis of randomized studies	**yes**	**yes**	**LVH: yes (HD)** **M: yes**	Reduction of NT Pro-BNP and PWV
Horowitz et al., 2023^167^	Systematic review and meta-analysis of randomized studies	**yes**	**ye**s	LVH: no ECV: no I: no **M: yes**	-

Abbreviations – BP: blood pressure; BIA: bioelectrical impedance; LVH: left ventricular hypertrophy; ECV: cardiovascular events; I: hospitalization; M: mortality; PWV: pulse wave velocity.

It is important to highlight the diversity of parameters derived from the use of BIA to assess the volume status of dialysis patients, with no consensus on a preferred choice among them^169,170^. Conversely, the literature is more robust in indicating the use of BIA as a support in hypervolemia control rather than as a primary guideline in the conduct of antihypertensive therapy. The use of BIA could impact BP control; however, it is crucial to realize that it should be seen as one of several available alternatives for managing volume status and BP.

Key message:

- BIA is recommended as a complementary method for assessing and managing hyperhydration and BP in HD and PD patients (Class I/Level A).

### Point-of-Care Ultrasound in the Assessment of Volume Status in CKD Patients: Evaluation of Heart, Lungs, and Inferior Vena Cava (IVC)

Volume status is a fundamental parameter across the entire CKD spectrum, and its clinical assessment has limitations, stimulating the development of more objective volume assessment tools, such as point-of-care ultrasound (POCUS)^171^. POCUS allows for a multi-organ approach, assessing various facets of the patient’s volume status.

**Figure 3:**
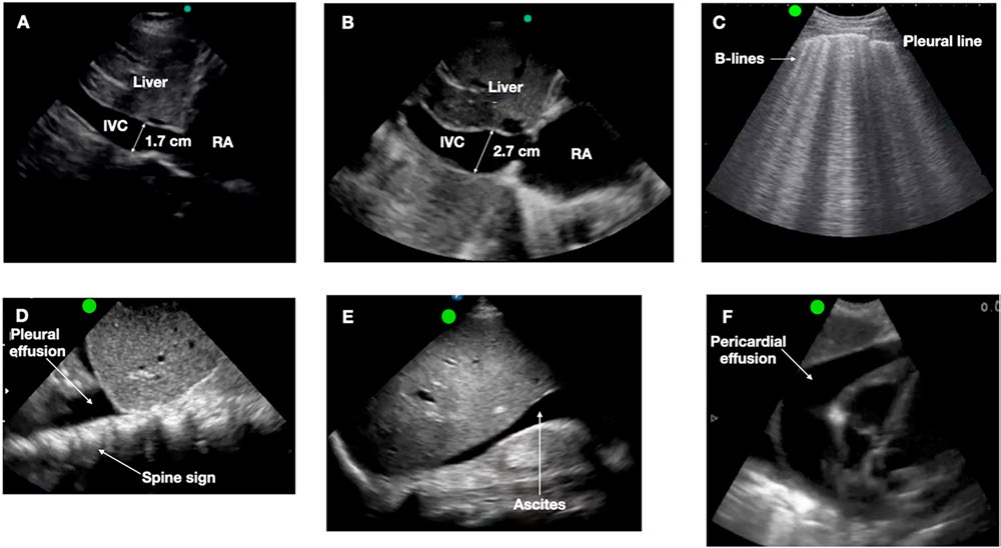
Point-of-care ultrasound for identifying volume status. Legend: A and B, ultrasound of the inferior vena cava (IVC): A, IVC < 1.7 cm, with a diameter decrease of more than 50% during inspiration, compatible with a euvolemic state; B, plethoric IVC, with a diameter of 2.7 cm and a decrease of less than 50% during inspiration, suggestive of hypervolemia; C, lung ultrasound: B-lines indicating interstitial lung syndrome, as seen in acute pulmonary edema; D, pleural effusion indicated by an anechoic image above the diaphragm; E, ascites, an anechoic image in the hepatorenal space; and F, pericardial effusion indicated by an anechoic image in the pericardial space.

The pump-pipes-leaks approach is a proposal for practical and objective assessment of volume status. Insonation of the heart through the parasternal long axis cardiac view enables LV systolic function to be assessed from the variation in its systolic and diastolic volumes, lateral wall and interventricular septum thickness in systole, and amplitude of septal displacement of the anterior mitral valve leaflet (pump)^171^. “Pipes” refers to the assessment of IVC diameter (Figure 3A-B).

**Figure 4:**
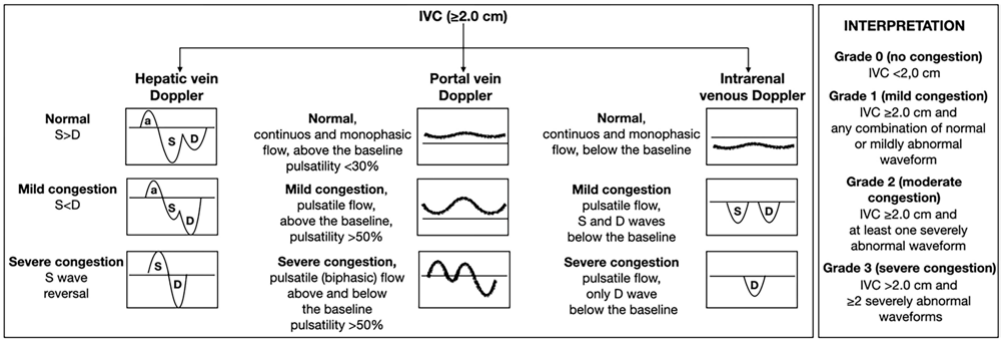
Doppler patterns of the hepatic, portal and renal interlobar veins in the context of mild, moderate and severe venous congestion.

A more objective model of volume assessment has been developed using Doppler examination of the hepatic, portal, and renal interlobar veins, known as Venous Excess Ultrasound (VExUS). Figure 4 summarizes the Doppler patterns of the hepatic, portal, and renal interlobar veins in the context of mild, moderate, and severe venous congestion. The low quality of evidence available for the use of VExUS in the volume assessment of CKD patients is noteworthy^168^.

Abbreviations – IVC: inferior vena cava; a: atrial contraction; S: systole; D: diastole. Note – based on Dinh V. POCUS 101 Vexus ultrasound score – fluid overload and venous congestion assessment.

Leaks denotes the search for pulmonary congestion through the identification of B-lines (Figure 3C) with high sensitivity, but lower specificity for congestion, as well as pleural effusion (Figure 3D), ascites (Figure 3E), and pericardial effusion (Figure 3F). This allows for the detection of extravascular congestion before the onset of clinical symptoms, thereby adjusting the treatment for dialysis patients^172^.

In dialysis patients, identifying DW is challenging. If overestimated, DW contributes to persistent volume overload and its consequences; when underestimated, DW may cause intradialytic hypotension and associated complications. In the context of dialysis treatment patients, POCUS can provide valuable information.

The pump-pipes-leaks strategy enables bedside answers to questions related to the interaction between absolute volume gain, multicompartmental redistribution of extracellular volume, and the multiorgan adverse impact. Further studies are needed to assess its impact on HD patient survival^172^.

Key message:

- It is recommended to use the pump-pipes-leaks volume estimation tool at the bedside, including the VExUS score, as a potential alternative for assessing the severity of venous congestion in CKD patients (Class II A/Level B).

## Blood Pressure Targets in Peritoneal Dialysis (PD) and Hemodialysis (HD)

BP targets in dialysis patients, regardless of the modality, remain controversial, and extrapolating BP targets from the general population to this subgroup does not seem the best choice. It has been documented in the literature, through observational studies, that there is a U or J-shaped curve in the BP control of HD and PD patients. However, there may be interpretation biases due to the inclusion of individuals with hypotension, severe CV disease or frailty, contributing to a poor prognosis^5,120^,^173,174^.

The CRIC Study (The Chronic Renal Insufficiency Cohort Study), a multicenter prospective cohort study, presented interesting results on the relationship between all-cause mortality and SBP. Among participants who initiated HD (n = 326), a U-shaped association was observed between SBP measured “in the dialysis unit”, presumably before the start of the session, and mortality (HR 1.26 for every 10-mmHg increase), while SBP “outside the dialysis unit” showed a linear association with mortality. These results suggest that the optimal target BP for treatment should be determined by ambulatory BP monitoring (ABPM), which is not always available, well tolerated, or considered cost-effective^100,175^.

An American study involving over 17,000 HD patients concluded that those with pre-dialysis SBP < 140 mmHg had significantly higher mortality, especially within the first 3 months after initiating HD^176^. Similar findings related to less unfavorable outcomes with BP control during the interdialytic period were found in a Brazilian center, which included 2,672 HD patients followed for 31 months^177^.

The best-designed study with the potential to define BP targets was the Blood-Pressure-in-Dialysis (BID), a pilot study that randomized 126 participants to two SBP targets measured in the clinic before the start of the dialysis session: intensive (110–140 mmHg) or standard (155–165 mmHg), with the primary objective of assessing feasibility and comparative safety over a one-year follow-up period. The study demonstrated the feasibility of the intervention; however, despite the protocol requiring investigators to adjust post-dialysis weight as an initial step towards reaching the target SBP, the intensive SBP goal was only achieved using additional antihypertensive drugs. The authors indicated that there may have been inadequate management of extracellular volume to obtain the dry weight^178^.

The Kidney Disease Outcomes Quality Initiative (2005) guideline, which has not been updated, arbitrarily recommends a pre-dialysis BP < 140/80 mmHg and post-dialysis BP < 130/80 mmHg in HD patients, primarily based on expert opinion (Class IIA/Level C)^179^.

Since BP is a biomarker of the “cardiovascular syndrome”, the HTN management should address the prevention and treatment of all its CV complications. In the absence of data from randomized controlled clinical trials in dialysis patients, treatment objectives should be extrapolated from available observational studies and those developed in the general population. Based on this premise, it is reasonable to aim for a pre-dialysis BP ≤ 140/90 mmHg or a home BP ≤ 130/80 mmHg (Class IIA/Level C). A group of experts participating in a KDIGO initiative that discussed controversies regarding volume and HTN management in dialysis chose not to recommend BP targets for patients on RRT, and defined that general strategies in the management of HTN apply to dialysis patients, such as “Individualizing BP targets and agents according to age, coexisting CV disease, and other comorbidities, in addition to treatment tolerance” and “Inquiring about postural dizziness and checking for orthostatic hypotension”^180^.

In PD patients, an observational study showed that SBP ≤ 110 mmHg was associated with increased mortality, and a protective effect was observed with SBP > 120 mmHg^181^. Thus, based on data from the general population and the CKD population, it is recommended that the target BP be < 140/90 mmHg in PD patients, in agreement with the International Society of Peritoneal Dialysis (ISPD) and other authors (class IIA/Level B)^120,182,183^.

In the absence of evidence from long-term, randomized, controlled clinical trials designed for the specific purpose of defining optimal BP targets in HD and PD, it is recommended that clinical trials be developed comparing different home BP measurement thresholds in relation to clinical outcomes and mortality^184,185,186,187,188^.

In Chart 7, the recommendations from the major guidelines that cite goals to be achieved in HD patients can be observed.

**chart 7:** Blood pressure targets in hypertension of dialysis patients according to the main Guidelines

Guidelines	Blood pressure target (mmHg)
KDOQI 2005^179^	Pre-dialysis: <140/90 Post-dialysis: <130/80
KDOQI 2015 (update)^186^	Not mentioned, citing lack of data
EURECA-m (ERA – EDTA) 2017^175^	Not mentioned, citing lack of data
Japanese Society for Dialysis Therapy 2012^187^	Pre-dialysis: <140/90
Brazilian Hypertension Guidelines 2020^4^	Pre-dialysis: ≤140/90 Post-dialysis: ≤130/80
2020 International Society of Hypertension Global Hypertension Practice Guidelines^188^	Not mentioned
International Society of Peritoneal Dialysis (ISPD) 2015^120^	Peritoneal dialysis: <140/90 mmHg

Key messages:

- It is recommended that BP targets should be pre-HD: ≤ 140/90 mmHg and post-HD ≤ 130/80 mmHg (Class IIA/Level B).
- It is recommended that the BP target in PD be < 140/90 mmHg (Class IIA/Level B).
- It is recommended to prescribe individualized goals for patients on HD and PD, according to age, degree of frailty, tolerance, presence of severe CV disease, and comorbidities (Class IIA/Level B).
- Randomized, controlled, and comparative clinical trials of BP targets are recommended for a high-quality evidence-based indication.

## Non-Pharmacological Treatment of HTN in PD and HD

### The Importance of Knowing, Achieving, and Maintaining Dry Weight

**Figure 5:**
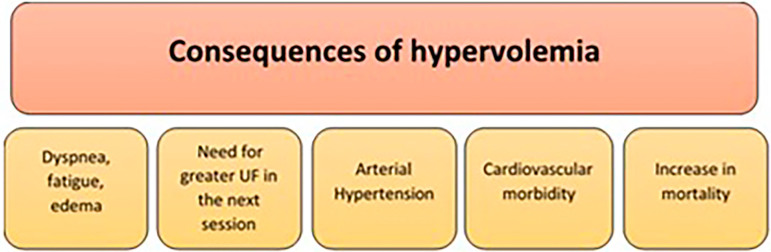
Consequences of maintaining hypervolemia.

DW represents the state in which there are no signs or symptoms of hypervolemia or hypohydration. For each HD session, a target weight needs to be defined (possibly higher than the DW), considering the volume status, the IDWG, and the tolerated UF rate, to avoid a target weight that is too low (because it could lead to hypotension and accelerate the loss of RRF)^9,105^, or inappropriately high, resulting in HTN and hypervolemia^9^. Chronic hypervolemia may cause HTN and an increased risk of hospitalization^189^ and death^163,190^ (Figure 5). The goal of achieving DW throughout the HD sessions is therefore prioritized, thereby avoiding chronic hypervolemia, even with a higher risk of intradialytic hypotension^105^.

Abbreviation – UF: ultrafiltration.

The regular application of a DW assessment protocol, preferably using multiple instruments^105^, has been associated with lower mortality, and the use of orthostatic BP measurement has been associated with a lower risk of hospitalization and CV events^191^. Nevertheless, the reliability of clinical signs in accurately estimating blood volume has been questioned^192^.

In PD, to achieve DW and BP control, priority is given to optimizing urine output with the use of diuretics (in patients with RRF)^193^ and peritoneal UF, with appropriate adjustments in glucose load considering the PD prescription and the peritoneal transport rate^5,9^. For these patients, it is recommended to adopt RRF preservation, dietary management, and efforts to limit peritoneal injury.

**Figure 6:**
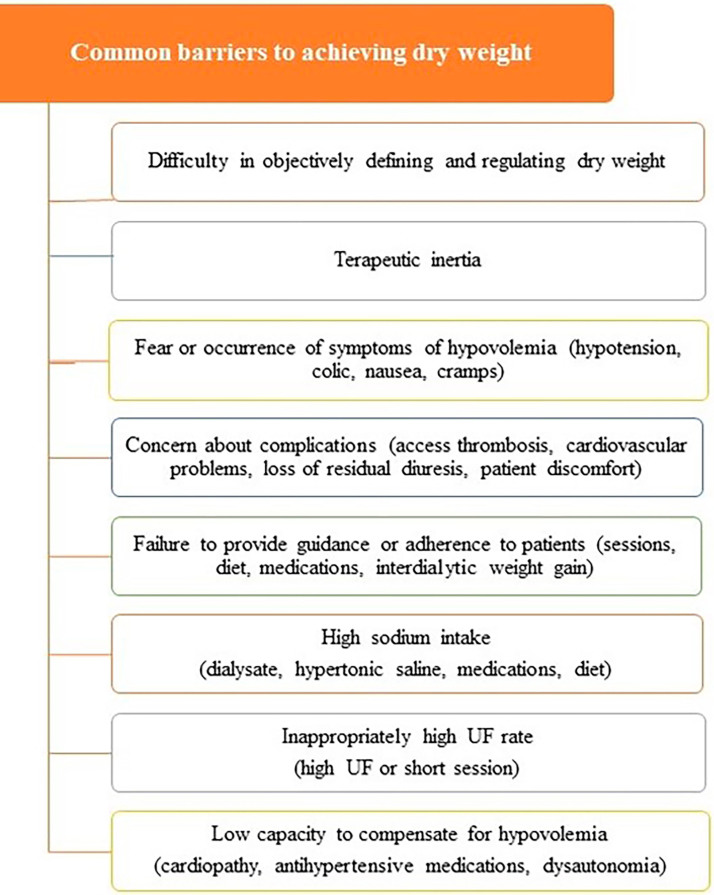
Common barriers to achieving dry weight in hemodialysis patients.

The barriers to achieving DW are depicted in Figure 6.

Abbreviations – UF: ultrafiltration; HTN: hypertension.

Key messages:

- For HTN control, the strategy of gradually adjusting UF to minimal hypovolemia symptoms is recommended, to define and reach DW (Class I/Level B).
- It is recommended that DW be assessed regularly and with multiple tools, such as clinical assessment, ultrasound, and bioimpedance (Class I/Level B).
- In PD, strategies for preserving RRF and the integrity of the peritoneal membrane are recommended (Class I/Level B).

### Changes in Dialysis Parameters in PD and HD

**Figure 7:**
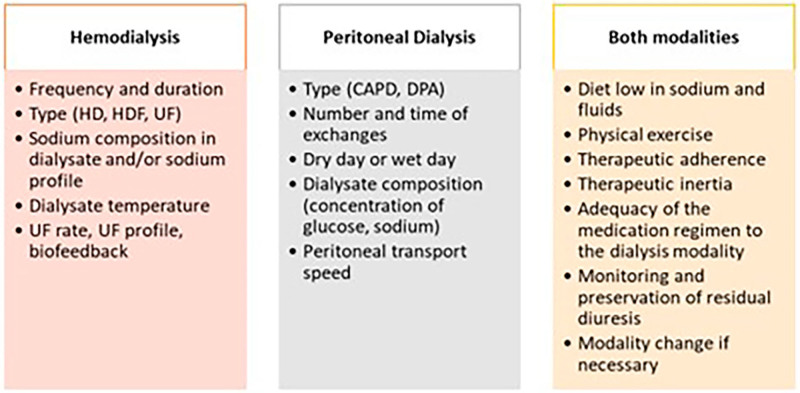
Factors related to dialysis modality and the patient that may affect blood pressure control.

There are factors related to dialysis parameters that could interfere with BP, both in HD and PD (Figure 7).

Abbreviations – HD: hemodialysis; HDF: online hemodiafiltration; UF: ultrafiltration.

Studies analyzing the sodium concentration in the HD dialysis solution are inconclusive as to its impact on BP and have already been detailed in Chapter 2, item 2.2.2 (Parameters interfering with BP)^27,29^. There is no evidence supporting a reduction in mortality when sodium is individualized^27^.

The benefit of sodium profiling used to avoid intradialytic hypotension and reduce sodium intake is controversial^103,191,194^. In one study, the use of this profile was associated with positive sodium balance, leading to higher IDWG rates, elevated BP, CV events, and death^191^. Another study showed that the linear profile did not reduce hypotension, although the “stepwise” profile proved effective^194^.

Methods for adjusting UF include the use of profiling, isolated UF followed by HD (sequential dialysis), or biofeedback devices^9,195^. A study evaluating linear UF profile showed no reduced risk of hypotension, nor differences in troponin levels and LV function^196^. UF biofeedback devices may reduce intradialytic hypotension^195^.

Reducing the dialysis solution temperature (in different definitions: a decrease in relation to body temperature, fixed at 35.5ºC or 36ºC, or adjusted using a biofeedback system) has been associated with a reduced intradialytic hypotension^197^ and CV mortality^191^, in addition to being well tolerated^197^. An ongoing study (MY TEMP, NCT02628366) will assess cooling dialysis solution in relation to hospitalization and death outcomes due to CV events.

In PD, the suggested maneuvers for HTN control include^9^: optimization of urine output with diuretics (in patients with RRF)^193^; reducing intraperitoneal residence time (of glucose-based solutions) in high transporters; glucose-based solutions with higher tonicity (less preferred due to peritoneal and systemic impact); icodextrin solution (increased UF without higher glycemic load)^198^, low-sodium dialysate, as it reduces HTN, without altering adequacy)^193^, and solutions with greater biocompatibility, neutral pH or lower content of glucose degradation products for preservation of the peritoneum and RRF^198,199,200^.

Key messages:

- Caution is recommended when adjusting the sodium concentration in dialysis solution for the purposes of BP and volume control (Class I/Level B).
- Caution is recommended in the use of sodium profiling to avoid intradialytic hypotension due to the risk of positive balance and its associated harms (Class I/Level B).
- It is not recommended that the use of linear UF profile be used to prevent episodes of intradialytic hypotension (Class I/Level B).
- Cooling dialysis solution is recommended to prevent intradialytic hypotension (Class I/Level A).
- For HTN control in PD, preservation of RRF and peritoneal membrane is recommended (Class I/Level B).

### Changes in Hemodialysis Modality (HDF, Daily HD)

Alternatives to conventional HD include intensive regimens (with increased frequency and/or length, such as in short daily HD and long daytime or nocturnal HD), and the use of convection (HDF), which may be performed in HD centers or at home. Daily HD is understood in the literature as any regimen of 5 to 7 times a week^201,202,203,204,205^.

The use of daily HD allows for a reduction in: BP^201,202,203^, the amount of antihypertensive drugs^203^, LVH^202^, tissue inhibitors of metalloproteinase^202^, and phosphorus, which in turn is associated with a reduction in FGF23^202^. A multicenter study of short daily HD in a home environment confirmed a reduction in the number of antihypertensive medications^204^. Intensive HD may also result in reduced hospitalizations (compared to conventional HD) and mortality (compared to conventional HD or PD)^205^.

Some studies have shown that nocturnal HD reduced SBP^202,206,207^, the number of antihypertensive drugs^207^, and LVH^202,207^, while also improving anemia^206^ and hyperphosphatemia^202,206,207^.

High-volume online hemodiafiltration (HDF) provides a lower risk of death from any cause, in contrast to high-flow HD^208^. A study evaluating peridialytic BP parameters showed no benefit of HDF over conventional HD^209^. In another study, HDF reduced CV mortality and episodes of hypotension^210^. To reduce intradialytic hypotension, the intermittent use of ultrapure dialysate infusions has been beneficial for the elderly and those with high IDWG^211^.

Key message:

- It is recommended, whenever available, to use intensive HD regimens (daily and/or long) with benefits in reducing BP (Class I/Level A), antihypertensive medication (Class I/Level A), and left ventricular mass (Class I, Level B).

### Dietary and Water Intake Recommendations for Hypertensive Patients on PD and HD, Especially to Prevent Interdialytic Weight Gain (IDWG)

It is recommended that dietary sodium intake for the hypertensive population (non-dialysis) should be up to 2 g/day, corresponding to 5.0 g/day of salt^4^. In individuals on dialysis, who are typically salt-sensitive, there is a greater impact of salt restriction on BP, suggesting a dietary intake of up to 1.5 g of sodium or 3.6 g of salt^5^. Compared to antihypertensive treatment, dietary sodium restriction associated with more intensive UF resulted in a reduction in IDWH, LVH, antihypertensive load, and episodes of intradialytic hypotension^212^.

A meta-analysis identified that reducing salt intake by at least 1 g was associated with a reduction in BP^213^. Conversely, high sodium intake was related to an increased risk of hypervolemia and death, but with no association with BP^214^.

In addition, hyperglycemia could increase thirst and salt intake, leading to an increase in IDWG and BP^215^. In dialysis patients, the Mediterranean diet appears to improve myocardial remodeling^216^, but has not been able to reduce mortality, similar to the DASH (Dietary Approaches to Stop Hypertension) and vegetarian diets^217^. In turn, polyphenol-rich diets have improved diastolic BP in the dialysis population^218^.

Key message:

- Dietary sodium restriction of 1.5 to 2.0g/day is recommended for dialysis patients to reduce BP and IDWG (Class I/Level B).

### Physical Exercise for Hypertensive Patients on Pd and Hd

Studies examining the effect of physical exercise on BP in dialysis patients have yielded divergent results. A meta-analysis did not identify an effect of physical training on BP^219^. However, in another meta-analysis consisting of 78 randomized studies with 3,326 participants, it was possible to highlight a greater reduction in DBP with combined aerobic and resistance exercises, involving patient-tolerated loads on the lower and upper limbs without AVF^220^. On the other hand, improvements are documented in parameters associated with CV outcomes, target organ damage, quality of life and cognitive function^219,220^. As for the types of physical exercise, evidence shows benefits from both aerobic and resistance exercises. Regarding the location, intradialytic or home-based exercises may be used^221^.

Key messages:

- It is recommended to encourage aerobic activity for a minimum of 30 minutes, at least 5 times a week, for both PD and HD patients (Class IIA/Level B).
- It is recommended that supervised resistance training should also be prescribed (Class IIA/Level B).
- It is recommended that physical activity be performed, either during dialysis sessions or in the interdialytic interval, outside of the dialysis setting (Class IIA/Level B).
- It is recommended that, whenever possible, physical exercises be supervised by physical educators and/or physiotherapists in the units (Class IIA/Level C).

### Other Lifestyle Changes (Spirituality, Stress Management, etc.)

Spirituality and religiosity are potentially important tools for dialysis patients, positively related to doctor-patient interaction, quality of life and life expectancy, coping with the disease, treatment and its consequences. They should therefore be considered by physicians^222^.

Spirituality may be understood as the pursuit of meaning and purpose in life, as well as the transcendence of the self. This experience may develop through religiosity and/or belief in God, family, naturalism, rationalism, humanism, and the arts, for example^222^. Religiosity, in turn, implies the human relationship with a transcendent being^222^.

These concepts correspond to a psychological construction in coping with chronic diseases, converting a personally challenging situation into a meaningful experience^223^. Strong religious beliefs among individuals on dialysis have been correlated with attenuated perceptions of the disease burden and an enhanced sense of social support^223,224^.

Adherence to dialysis is influenced by religious faith, age, and education: (a) Muslims have the desire to live and are less likely to discontinue dialysis^215,223^; (b) Christians with both extrinsic religiosity (religious behavior) and intrinsic religiosity (strong beliefs and commitments) show greater adherence^223^; (c) older age and longer dialysis vintage correlated with better adherence^223,225^; (d) patient counseling and education provide better results, increased adherence, and a potential reduction in healthcare-related costs^223^.

In some dialysis centers, social workers and psychologists have supported and encouraged non-formal religious activities (religious literature, prayer, discussion groups) as positive coping methods^223,226^.

Key message:

- It is recommended to respect and encourage religiosity and spirituality as a means of improving adherence and coping with dialytic CKD, involving the multidisciplinary team (Class I/Level B).

## Pharmacological Treatment of HTN in PD and HD

There is an apparent paradox regarding the use of antihypertensive drugs and BP control in dialysis patients, since the greater the number of medications, the greater the likelihood that BP is not controlled, suggesting that the combined use of different antihypertensive drugs may hinder the achievement of the target dry weight^227^. Antihypertensive medications should be used, if necessary, after volume control, preferably those assessed in RCTs. However, these studies are not widely available and have a limited number of participants, making it difficult to generalize guidelines for the use of antihypertensive drugs in dialytic CKD. The frequent CV impairment and the presence of comorbidities in dialysis patients make the combination of factors so diverse and unique that some guidelines recommend an individualized approach regarding the classes of drugs to be used^3,9,48^.

The use of antihypertensive medication in dialysis patients provides benefits, reducing overall and CV morbidity and mortality^228,229^. All classes of antihypertensives could be used for BP control in dialysis patients, if risks and benefits are considered^5^.

### Diuretics

The use of these drugs is not supported for HD or PD patients without residual diuresis (< 100 mL/day). In PD patients, diuretics may improve volume status and minimize the need for solutions containing a higher glucose concentration, although the International Society of Peritoneal Dialysis Guidelines do not recommend their use^120^. In HD patients, diuretics could help reduce IDWG, resulting in reduced UF rates and fewer intradialytic hypotension episodes^230,231^.

In a prospective cohort study involving ^5,219^ patients who continued using loop diuretics after the initiation of HD compared to ^6,78^ controls who did not, lower rates of hospitalization, intradialytic hypotension, and IDWG were observed, with no significant difference in BP or mortality rate within the first year of dialysis. In the USA, the most used diuretic in HD patients is furosemide, at doses of 20 to ≥ 320 mg/day^232^.

A prospective observational study followed 16,420 HD patients across three continents and observed that the use of diuretics (> 90% loop diuretics) in the first 3 months of HD was higher in Japan (47.8%) and Europe (45.1%) compared to the United States (26.4%), with a progressive decline over this period^233^. In this study, diuretic use was associated with lower IDWG and a lower probability of hyperkalemia. Patients with RRF undergoing diuretic therapy were nearly twice as likely to maintain residual diuresis after one year of follow-up compared to those not using diuretics. Patients receiving diuretics also had a lower risk of overall mortality (–7%; p = 0.12) and CV mortality (–14%; p = 0.03)^233^.

No studies were found evaluating the use of thiazide or thiazide-like diuretics as monotherapy in PD or HD patients. A study with high-dose triple diuretic therapy (furosemide 1000 mg/day, hydrochlorothiazide 100 mg/day, and spironolactone 50 mg/day) in 51 PD patients demonstrated increased diuresis and improved volume control compared to furosemide alone^234^.

A meta-analysis including ^28,226^ HD patients revealed a potential benefit in reducing intradialytic hypotension, CV and all-cause mortality^231^.

Key message:

- The use of loop diuretics is recommended in HD and PD patients if residual diuresis is present (Class I/Level B).

### Renin-Angiotensin-Aldosterone System (RAAS Inhibitors

RAAS inhibitors, including ACE inhibitors and ARBs are the most used antihypertensive medications in CKD at its different stages. A meta-analysis of randomized clinical trials evaluating the antihypertensive effect of different classes of drugs compared to placebo or to each other found that ACE inhibitors/ARBs modestly reduced BP when compared to placebo (–4.3 mmHg in SBP), as expected for the hypervolemic state typical of CKD in the dialysis phase^235^. When compared to placebo, mineralocorticoid receptor antagonists (MRAs) are the most powerful in reducing BP (–10.8 mmHg), followed by BB (–8.7 mmHg) and CCB (–4.6 mmHg)^235^.

In dialysis patients, the use of ACE inhibitors or ARBs is associated with a reduction in left ventricular mass index, morbid events - especially HF - and CV mortality^236,237,238^. Furthermore, there is evidence that the renal protection provided by these classes of drugs continues at this stage of the disease, as their use is associated with the maintenance of renal function or residual diuresis, which allows for better volume control and reduces the risk of intradialytic hypotension^239,240^. In PD patients, the use of ACE inhibitors/ARBs is preferably associated with preservation of the peritoneal membrane, with a reduction in fibrosis and maintenance of peritoneal clearance and UF^241,242^. The risk of hyperkalemia is plausible but controversial, as it is not observed in all studies^243,244,245^.

Key messages:

- The use of ACE inhibitors or ARBs in HD and PD patients is recommended as antihypertensive agents due to their pleiotropic effects: reduction of LVH, HF, reduction of peritoneal fibrosis in PD, and CV mortality (Class I/Level B).
- Monitoring serum potassium levels is recommended, although the risk of hyperkalemia is controversial and not always observed (Class IIA/Level B).

### Beta-Blockers (BB)

In dialytic CKD, BB are recommended as the preferred medication. Two RCTs have demonstrated the efficacy and CV protection provided by BB in a subpopulation of dialysis patients. The first, a placebo-controlled RCT, showed that carvedilol used in 114 HD patients with HF (dilated cardiomyopathy and reduced ejection fraction) resulted in clinical improvement, a reduction (–49%) in overall and CV mortality (–68, 0%) and in hospitalizations due to HF (–81%)^58^. The second study aimed to compare the effect of reducing LVH with atenolol or lisinopril (administered following HD sessions) in HD patients with BP ≥ 140/90 mmHg and LVH. However, it was halted early because the interim results were so beneficial in favor of atenolol that it would have been unethical to continue the study^246^. Although with a limited number of participants, 100 in each group, the study has become a reference. The rates of severe CV events and hospitalizations were at least halved in the atenolol-randomized group when compared to lisinopril^246^. The 44-hour ABPM was consistently lower in the group receiving atenolol, even though participants in the lisinopril group had received more antihypertensive medication and reduced their dry weight by an additional 3 kilos during follow-up, indicating the superiority of atenolol over lisinopril^246^. Other studies with BB (carvedilol, bisoprolol, and atenolol) have shown similar results regarding BP reduction, on average –8.7 mmHg in SBP^235^.

There is controversy in the literature regarding the dialyzable BB (atenolol, bisoprolol, metoprolol, nadolol) and the non-dialyzable ones (carvedilol, nebivolol, propranolol, pindolol)^247^. Some studies present objective data and suggest that dialyzable BB are superior to the non-dialyzable ones, as they show a reduction in major CV events (stroke, AMI, and CHF), and in both CV and overall mortality, when compared^248,249^. There is also a meta-analysis of observational studies that found no differences in the CV protective effects between dialyzable and non-dialyzable BB in individuals undergoing HD^250^. All studies that have evaluated possible differences in CV protection between dialyzable and non-dialyzable BB are observational, either retrospective or prospective cohorts, and based on drug prescription data.

Regardless of the BB, an important question arises: “Could its association with other antihypertensive drugs have an additional beneficial effect?” At least one observational study has evaluated this aspect in HD patients who developed HF after the initiation of dialysis and started using BB, ACE inhibitors/ARBs, or both^251^. In the 5-year mortality follow-up, patients who used only BB were considered a reference for those who used only ACEI/ARBs, those who used both BB and ACEI/ARBs, and for those using any other drugs. Compared to the reference (use of BB alone), mortality for those using only ACE inhibitors/ARBs was similar (+8%; not statistically significant). For those who used a combination of BB and ACE inhibitors/ARBs, mortality was lower (–33%; p < 0.001), while for those who used neither BB nor ACE inhibitors/ARBs, mortality was higher (+74%; p < 0.001)^251^.

Key messages:

- Beta-blockers are recommended as preferred drugs for HD and PD patients, unless contraindicated (Class I/Level B).
- Preferential use of atenolol is recommended, unless contraindicated (Class I/Level B).
- There is insufficient evidence on the preferential use of dialyzable *vs.* non-dialyzable BB (Class IIB/Level B).

### Calcium Channel Blockers (CCB)

There are few studies evaluating CCB in the treatment of HTN and the prevention of CV complications in dialysis patients. A meta-analysis of randomized studies^235^ found only 3 studies with CCB compared to placebo (nitrendipine, diltiazem, and anlodipine), in which, on average, the hypotensive effect was similar to that of ACE inhibitors (–4.6 mmHg in systolic BP). The most robust RCT included 251 patients and compared anlodipine *vs.* placebo. It demonstrated a mean reduction of –10 mmHg in SBP, a non-significant reduction in overall mortality (primary outcome; RR = 0.62), and a significant reduction (RR = 0.53, p = 0.03) in secondary outcomes (AMI, stroke, myocardial revascularization, and peripheral artery disease requiring revascularization or amputation)^252^. It was observed that the hypotensive effect of nitrendipine was more pronounced in patients with higher IDWG^253^.

A systematic review with meta-analysis identified 13 randomized or quasi-randomized studies involving the use of CCB in dialysis patients^254^. Although the number of patients included in this analysis was large (1,459), the diversity of the studies and the low level of confidence limited the conclusions^254^.

Key message:

- The use of CCB in HD and PD patients is recommended, as they maintain their antihypertensive and CV-protective effects, even in the presence of hypervolemia (Class I/Level B).

### Mineralocorticoid Receptor Antagonists (MRA)

A meta-analysis including 1,133 HD patients in 11 studies on MRA (10 with spironolactone and 1 with eplerenone) observed a greater hypotensive effect when compared to placebo (–10.8 mmHg in SBP) and higher drug discontinuation rates, although with no additional risk of hyperkalemia^235^.

Another meta-analysis, which included 1,309 HD patients, in 14 RCTs involving the use of MRAs (13 with spironolactone) *vs.* placebo or no treatment, demonstrated a significant reduction in non-fatal CV events (–49%), a reduction in overall mortality (–56%) and CV mortality (–59%), without causing statistically significant hyperkalemia^255^.

A meta-analysis including 829 dialysis patients from 9 RCTs on MRA observed a significant reduction in overall mortality (–60%) and CV mortality (–66%). However, a 3-fold increase in the risk of hyperkalemia and up to a 5-fold increase in the risk of gynecomastia were observed^256^. In the available meta-analyses, although hyperkalemia may pose a risk, it does not appear to mitigate the beneficial effect on CV and overall mortality^255,256^.

Two large ongoing RCTs could provide more reliable data on the efficacy and safety of spironolactone use in dialytic CKD. The Aldosterone Antagonist Chronic Hemodialysis Interventional Survival Trial (ALCHEMIST), which has already recruited 825 HD patients and is due to be completed in 2024, evaluates the effects of spironolactone on clinical outcomes of interest. The Aldosterone Blockade for Health Improvement Evaluation in ESKD Trial (ACHIEVE) plans to enroll 2,750 dialysis patients and compare the effects of spironolactone in relation to CV death or hospitalization.

It is worth noting that the existing studies predominantly use spironolactone, with a higher risk of gynecomastia, and a low and controversial risk of hyperkalemia (Class I/Level B). To date, there has been no RCT using selective non-steroidal MRAs in HD or PD.

Key message:

- The use of MRAs in HD patients is recommended, given their good antihypertensive effect and their capacity to reduce CV and overall morbidity and mortality (Class I/Level A). For PD patients, the number of studies and the evidence are weak (Class IIB/Level B).

### Other Classes of Antihypertensives

#### Centrally acting sympatholytics

There are no studies involving methyldopa, and a systematic review with meta-analysis concluded that there is no evidence to support the chronic use of clonidine in HD. In addition, its use is associated with a significant side effect profile^257^.

#### Direct-acting vasodilators

There have been no studies of direct-acting vasodilators such as hydralazine and minoxidil in the dialysis population. Hydralazine has been used in association to isosorbide in HD patients with HF with reduced ejection fraction, but not as an antihypertensive agent^258,259^.

However, sympatholytics and direct vasodilator agents are used empirically in dialytic CKD, particularly in resistant and refractory HTN. A study conducted in 210 dialysis clinics in the United States found that in the first 6 months of dialysis, sympatholytics are used by 19% of patients, while vasodilators are used by 4% to 10%^260^.

Key message:

- It is recommended that centrally acting sympatholytics and direct vasodilators be used as the 5th or 6th antihypertensive medication in dialysis patients, or if there are contraindications to the other antihypertensive drugs (Class IIA/Level C).

### Renal and Hemodialysis Clearance of Antihypertensive Drugs

The need for dose adjustment or post-dialysis replacement of the prescribed antihypertensive medication is a recurring concern for nephrologists when monitoring HD patients. Chart 8 shows a summary of antihypertensive drugs available in Brazil that are either renally cleared or more extensively dialyzable, and therefore may require adjustment. Antihypertensives not mentioned do not require adjustment or post-HD replacement^261^.

**Chart 8:** Main antihypertensive drugs available in Brazil. Dose adjustment in chronic kidney disease and rate of removal by dialysis*

Drug class and medications	Drug adjustment in CKD	Dialysis removal
**ACE Inhibitors**		
**Captopril**	**GFR < 10 mL/min:** administer 50% of the dose every 24 hours	**HD:** administer an extra dose after HD **PD:** insignificant removal
**Enalapril**	**GFR ≤ 30 mL/min:** 2.5 mg every 24 hours. Increase progressively according to BP	**HD:** clearance rate 20% - 50%. Extra dose of 2.5 mg after HD **PD:** adjust to 25% of the usual dose
**Benazepril**	**GFR ≤ 30 mL/min:** 5 mg every 24h. Increase progressively according to BP	**HD:** adjust the dose to 25% - 50% of the usual dose. No extra dose post-HD **PD:** adjust the dose to 25% to 50% of the usual dose
**Lisinopril**	**GFR 10-30 mL/min:** 2.5 mg - 5mg every 24 hours. Increase progressively according to BP **GFR < 10 mL/min:** consider replacing with another medication (high risk of AE).	**HD:** 50% clearance rate. The use of 10 mg after HD (3 times/week) is recommended, increasing the dose progressively according to BP **PD:** 2.5 mg every 24 hours. Increase progressively according to BP
**Ramipril**	**GFR ≤40 mL/min:** 25% of the usual dose.	**HD:** insignificant removal **PD:** insignificant removal
**Perindopril**	**GFR <30 mL/min:** not recommended	**HD:** post-HD use, if so **PD:** not recommended
**Beta-blockers**		
**Atenolol** **Bisoprolol** **Metoprolol** **Nadolol**	**GFR >30 mL/min:** no need for dose adjustment **GFR 10-30mL/min:** half ofdaily dose **GFR <10mL/min:** ¼ of daily dose	**HD:** removal rate 25% to 50%. Use of dose after HD. **PD:** insignificant removal. Use ¼ of daily dose
**Propranolol** **Pindolol** **Carvedilol** **Nebivolol**	No need for dose adjustment	**HD:** insignificant removal **PD:** insignificant removal
**Sympatholytic drugs**		
**Alpha-methyldopa**	**GFR <10 mL/min:** Administer every 12 to 24 hours	**HD:** removal rate of up to 60%. Use one dose after HD. **PD:** Administer every 12 to 24 hours.
**Clonidine**	**GFR < 30 mL/min:** start with a low dose and increase slowly according to BP and EA	**HD:** insignificant removal **PD:** insignificant removal
**ARB**		
**Angiotensin Receptors Blockers**	No need for dose adjustment	**HD:** insignificant removal **PD:** insignificant removal
**CCB**		
**Calcium Channel Blockers**	No need for dose adjustment	**HD:** insignificant removal **PD:** insignificant removal
**Diuretics**		
**Loop Diuretics**	No need for dose adjustment	**HD:** insignificant removal **PD:** insignificant removal
**Alpha-blockers**		
**Alpha-blockers**	No need for dose adjustment	**HD:** insignificant removal **PD:** insignificant removal
**Vasodilators**		
**Direct Vasodilators**	No need for dose adjustment	**HD:** insignificant removal **PD:** insignificant removal

Abbreviations – ACE: angiotensin-converting enzymes; GFR: glomerular filtration rate; CKD: chronic kidney disease; HD: hemodialysis; PD: peritoneal dialysis; AE: adverse effects; ARB: dos angiotensin receptors blockers; CCB: calcium channel blockers. Notes –

*Chart adapted from reference^261^. Information for dose adjustment and removal during dialysis based on UpToDate 2021 recommendations for each drug listed.

### Timing of Administration or Suspension of Predialysis Drug Dosing

Intradialytic hypotension is always a risk for HD patients, particularly if there is excessive IDWG or use of drugs that block defense mechanisms in the event of transient hypovolemia during HD (sympatholytics, BB, and ACE inhibitors/ARBs). It is therefore tempting to reduce the dose or refrain from administering some medication(s) on the day of HD or in the hours prior to it. There is a single cluster-randomized study, conducted in five centers that maintained antihypertensive medication (65 participants) and five centers that discontinued medication on the day of HD (n = 66)^98^. In both groups, the medication used in a single daily dose was maintained as a bedtime dose. The study revealed that there was no reduced risk of intradialytic hypotension nor was there a higher frequency of reaching the estimated dry weight in the group where medication was discontinued on the day of HD. However, there was a higher risk of pre-dialysis hypertension (SBP > 160 mmHg)^98^.

Key message:

- There is no evidence of benefits in suspending or reducing the dose of antihypertensive medication on the day of HD (Class III/Level B).

### Combination Therapy

The classes of antihypertensive drugs with the most appropriate profile, considering the effect on BP, the reduction in CV complications, and safety, would be: BB, followed by CCBs and ACE inhibitors/ARBs, and finally MRA. However, this is not the reality in clinical practice. Most patients start their dialysis treatment using BB, ACE inhibitors/ARB, and CCB^260^. Thus, the most reasonable initial strategy should be to control hypervolemia, reaching the estimated DW, avoiding the combination of antihypertensives, and (re)introducing them according to the characteristics of each patient, in the order suggested above. Other antihypertensive drugs could be added, if necessary, only after euvolemia has been reached.

## Special Situations of Hypertension in Dialysis

### Hypertension in Pregnant Women on DP and HD

The prevalence of pregnancies in women on dialysis is estimated at 1% to 7%, being more frequent in HD patients than in those on PD^262,263^. Chronic hypertension in early pregnancy is an important factor for maternal complications, but it is not associated with neonatal complications (1/5 = 20.0% *vs.* 2/9 = 22.2%, with no chronic hypertension)^262,263^. Recently, an increase in the number of pregnancies in women with CKD has been observed, possibly due to improvements in HTN management and a reduction in complications such as polyhydramnios^264^.

The most common maternal complications in women with CKD, whether dialytic or not, during pregnancy are hypertensive disorders. Identifying the difference between preeclampsia and decompensation of CKD may be challenging. HTN in these patients can be related to fluid overload, and its management with UF could lead to target organ hypoperfusion, including the placenta^262^. There are no randomized clinical trials establishing the optimal BP for pregnant women with CKD on dialysis^265^. Available studies typically follow the HTN management recommendations from the major obstetric guidelines^263,264,265,266^. In addition, strict BP control is necessary, with a target pressure < 140/90 mmHg or a DBP < 85 mmHg, according to the CHIPS study^267^.

Pregnancy in dialysis patients shows better outcomes when dialysis time and dose are intensified, based on serum urea levels (below 50–70 mg/dL)^265^. A systematic review highlighted a higher risk of pregnancy complications in women with CKD, including preeclampsia (odds ratio [OR] 10.4, 95% CI 6.3–17.1), preterm birth (OR 5.7, 95% CI 3.3–10.0), intrauterine growth restriction or low birth weight (OR 4.9, 95% CI 3.0–7.8), caesarean sections (OR 2.7, 95% CI 2.0–3.5), and pregnancy failures [including stillbirth and fetal and neonatal death] (OR 1.8, 95% CI 1.0–3.1), compared to healthy women in the control group^264^.

The live birth rate on dialysis has improved significantly over the decades, from 25% in 1960 to over 75% currently. However, 53.4% of babies are still born prematurely, and 65% have a low birth weight, i.e. less than 2.5 kilos^268^. A review that analyzed 10 articles revealed that most pregnant women on HD follow therapeutic regimens ranging from 15 to 40 hours a week (in daily or four-times-a-week regimens), urea target below 60 mg/dL, and creatinine target of 6 mg/dL. The same review demonstrated that most patients use a dialysate flow rate of 500 mL/min, while blood flow and the type of dialyzer varied considerably^269^. Another systematic review identified an overall live birth rate of 82%, with a positive relationship between the number of hours on dialysis and better outcomes, including a reduced risk of preterm birth before 37 weeks of gestation and small-for-gestational-age newborns below the tenth percentile^270^.

Key messages:

- HTN management in pregnant women on PD or HD should be conducted in accordance with the guidelines established for BP control in the general population.
- Increased dialysis time and dose are related to better maternal and fetal outcomes (Class I/Level 2A).

### Resistant (RHT) and Refractory Hypertension (RFHT) in Peritoneal Dialysis and Hemodialysis Patients

According to the main Guidelines, resistant hypertension (RHT) is characterized by a lack of BP control (usually BP < 140/90 mmHg) for more than 3 months, even with the use of three antihypertensive drugs, preferably a thiazide diuretic, a RAAS inhibitor, and a CCB, or the use of four BP-controlling drugs^271^. RfHT, in turn, occurs when BP remains outside the therapeutic target for more than 6 months, even with the use of five or more drugs, including a MRA (spironolactone) and a long-acting thiazide diuretic (chlorthalidone)^272,273^.

To diagnose these conditions, it is important to assess treatment adherence and confirm uncontrolled BP using ABPM or HBPM, according to the protocols discussed in Chapter 3.

Although the pathophysiological mechanism of RHT is directly related to volume overload, which is worsened in the presence of CKD 5D, clinical practice shows that many patients, upon initiating dialysis treatment, still have elevated BP values, despite adequate UF. Studies report a prevalence of RHT ranging from 18%^274^ to 24%^6^.

For better control of RHT and RfHT, it is important to ensure adequate volume control, achieving optimal DW, as well as individualized prescription of sodium in the dialysis fluid, appropriate dialysis length, and restriction of salt intake in the interdialytic period^274^.

Pharmacological treatment for patients undergoing dialysis with RHT or RfHT has some particularities. The use of diuretics is not recommended for patients without RRF, and pharmacological treatment follows the recommendations already described in Chapter 7. A non-randomized intervention study has highlighted the BP response to sacubitril/valsartan in these patients, albeit with a small sample size and short duration^275^.

The use of spironolactone has been shown to be poorly effective for BP control, but it has a positive effect on reducing overall mortality and the incidence of CV events^276^.

In more challenging cases of BP control in RHT or RfHT patients, once the options of other antihypertensive classes have been exhausted, direct vasodilators such as hydralazine or minoxidil may be considered as therapeutic options^277^. Furthermore, the use of renal denervation in RHT on dialysis has been proposed, although this approach remains a matter of debate. Recent studies, such as RCTs, have shown a favorable short- and long-term BP response in patients undergoing this intervention^278,279^. The pharmacological treatment follows the recommendations established in Chapter 7.

Key messages:

- It is recommended that, for the diagnosis of true RHT and RfHT, adherence to treatment should be checked and confirmed, preferably by ABPM or HBPM (Class I/Level A).
- Renal denervation may be a therapeutic option in selected patients (Class 2B/Level C).

### Systolic Hypertension in the Elderly Undergoing Dialysis

Isolated systolic arterial hypertension (SHTN) is highly prevalent in elderly patients due to the impaired elasticity of the thoracic great vessels. Conversely, it is known that SHTN, with or without elevated diastolic levels, assessed during the interdialytic period by ABPM or HBPM, is diagnosed in more than 70% of CKD 5D patients^280^. Thus, elderly patients initiating dialysis treatment, like all other subpopulations, may experience accelerated vascular damage, characteristic of CKD 5D patients^281^.

Elderly patients on dialysis may present with SHTN, even after reaching their DW. In such cases, it is necessary to consider the possibility that increases in systolic levels are the result of central vascular stiffness^274^. The so-called pseudo hypertension should be included in the differential diagnosis of these patients^4^. It is not uncommon to prescribe gradual fluid removal through UF to normalize BP in these conditions. However, it is important to consider that elderly individuals with pseudo hypertension may develop intravascular hypovolemia with severe CV consequences. Additionally, it is important to consider that elderly hypertensive patients on dialysis may also have secondary hypertension (see item 8.5) and other HTN phenotypes, such as WCH and MH (see item 3.4.3), and that clinical investigations should be conducted as minimally invasively as possible.

The lack of RCTs on the harmful effects of SHTN in dialysis patients leads to all recommendations being based on expert opinions. The situation becomes more complex when it comes to suggestions for the elderly undergoing dialysis therapy.

### Arterial Hypertension in Hemodialysis Associated with High-Flow Arteriovenous Fistula

The hemodynamic effects of the creation of an arteriovenous fistula (AVF)^282^ have been studied in patients requiring HD. Before creating an AVF, it is crucial to perform a comprehensive cardiac function assessment, with an emphasis on the RV, complemented by information on the LV. Together, a detailed CV evaluation, electrocardiogram and transthoracic echocardiogram enable cardiac functions and structures to be assessed prior to AVF. Furthermore, it is essential to perform a thorough assessment to establish the optimal diameter of the arteriovenous anastomosis, ensuring that the consequent flow reduction manages to lower BP, with benefits outweighing the risk of volume overload and increased pressure on the RV.

Significant hemodynamic changes occur due to the redistribution of fluids from the high-pressure, low-capacitance arterial system to a low-pressure, high-capacitance system, resulting in reduced peripheral resistance, an increased RV preload and LV afterload^283^. Clinical studies have shown that SBP and DBP decrease in the short and long term after the creation of an AVF, but increase following its ligation^284,285,286^. Scholz et al.^283^, in a meta-analysis, confirmed these findings.

It is essential to assess the hemodynamic and cardiac effects of an AVF and its possible complications, such as right heart failure caused by increased venous return, RV overload, and LV remodeling. Additionally, side effects such as venous stenosis, increased pulmonary BP, and right ventricular dysfunction may occur due to the increased venous flow^284,285,286^. These alterations may lead to the need for AVF closure in symptomatic patients^287^. It is crucial to consider the long-term effects of this artificially increased volume load on the right heart. Reddy et al.^288^ have demonstrated that the creation of an AVF resulted in significant right heart dilatation and deterioration in right ventricular function, consequently leading to heart failure in over 40% of patients. Conversely, the creation of an AVF may lead to a modest reduction in the left ventricular size and mass, as well as slowing CKD progression in some cases^288^.

### Secondary Hypertension in Dialysis

Dialysis patients who meet the established criteria for RHT/RfHT (see item 8.2) are candidates to be assessed for possible underlying causes, in addition to the CKD condition itself, which could alone justify the difficulty in BP management^5^. Patients with secondary endocrine or non-endocrine causes are rarely identified, and the use of medications that may lead to uncontrolled BP - such as nonsteroidal anti-inflammatory drugs, recombinant human erythropoietin, and corticosteroids - should be particularly noted^84^. Another non-endocrine cause highly prevalent among chronic kidney disease patients, including over 50% of those on HD and PD, is OSA, which should be recognized and treated^78,289,290^. All possible etiologies should be considered and investigated in clinically and laboratory-selected patients, after achieving the appropriate dry weight.

### Complications of HTN in Dialysis Patients

The complications of HTN in dialysis patients are eminently CV and fall within the spectrum of target organ damage due to HTN in patients with normal renal function (Chart 9). However, due to multiple other CV injury mechanisms in CKD, such as systemic inflammation, endothelial dysfunction, arterial calcification, hyperparathyroidism, anemia, salt overload, and the presence of AVF, it is difficult to demonstrate an independent relationship between HTN and CV complications in dialytic CKD. There are very few studies reporting improvement in these complications with the treatment of HTN, which is a limiting factor for conclusions.

**Chart 9:** Cardiovascular complications of arterial hypertension in dialysis chronic kidney disease

Left ventricular hypertrophy
Heart failure
Coronary heart disease
Sudden cardiac death
Atrial fibrillation
Stroke
Peripheral arterial obstructive disease (including carotid territory)
Aortic aneurysms (thoracic, abdominal)
Occlusion of the central retinal artery or central retinal vein

The most important association is that with clinically manifest CV outcomes such as HF, CAD, sudden cardiac death, and stroke. The relationship between HTN and CV events in dialytic CKD is complex. Studies indicate a U-shaped association between the BP obtained in the dialysis unit and CV events, both coronary and cerebral^3^, as well as with ambulatory BP measurements in PD^291^. Events occur at the highest rates in patients with pre-dialysis SBP below 110–120 mmHg. The lowest incidence of events is seen at SBP values between 140–160 mmHg, and there is a slight but significant increase above these levels. There are two possible explanations, supported by evidence, for this paradox. One explanation refers to the method of BP measurement, particularly that measured outside the dialysis unit, as some studies suggest that the use of ABPM instead of peridialytic BP converts the U-shaped relationship into a linear one, more similar to what is observed in hypertensive patients with no kidney disease^3,5^,^99,292,293^. Similarly, in PD, the absence of a linear relationship between BP and CV outcomes becomes positive when based on SBP values obtained with ABPM^294^. The other explanation is related to the coexistence of cardiac dysfunction in patients with lower BP. A study showed that in patients assessed by ABPM, an analysis excluding patients with HF or AF transformed a U-shaped relationship into a positive linear relationship between BP and outcomes^295^.

LVH is a common complication of HTN that often precedes the onset of HF and predisposes to arrhythmias that lead to sudden cardiac death. Despite the presence of various other factors in the genesis of LVH in dialysis, HTN is an independent causal factor in several studies^296^. A recent study showed that LVH in HD patients progressively increased with a pre-dialysis SBP rise (1.9× between 131–139 mmHg, 7.7× between 140–149 mmHg, and 12.9× if > 150 mmHg)^297^. Unfortunately, it is not clear whether lowering BP results in improved LVH in dialysis patients. In the BID (BP in Dialysis) study, there were no differences in LV mass between patients randomized to intensive BP control (SBP 115–140 mmHg) *vs.* the control group (SBP 155–165 mmHg) after one year of follow-up^178^. Conversely, salt restriction and prolonged dialysis, two interventions that result in better BP control, provide regression of LVH in dialysis patients^298^. ARBs and ACE inhibitors lead to LVH regression in HD and PD patients^299^, which is not the case with aldosterone receptor blockers^300^. It is not defined whether this effect is mediated by BP reduction or by other mechanisms.

Atrial Fibrillation (AF) has an average prevalence of 11.6% and an average annual incidence of 2.7% in dialytic CKD^301^. While the relationship between HTN and AF is strong in patients without CKD, this association in dialysis is not consistent across publications. Among the largest studies evaluating the issue, there was an approximately 22% increase in the risk of chronic AF in hypertensive patients in a USRDS study^302^, while two studies from other databases (Medicare/Medicaid and DOPPS)^303,304^ demonstrated a decreased risk of AF in the presence of HTN, both in a binary manner (21% reduction)^304^ and linearly (6% reduction for every 10 mmHg increase in pre-HD SBP)^303^. There are no studies analyzing the impact of HTN treatment on the incidence of AF in dialysis patients.

PAD is common in patients with CKD. The relationship between HTN and PAD in dialysis patients is inconsistent. In the CRIC study, HTN was not a risk factor for PAD^305^. On the other hand, the DOPPS study showed a significant relationship between HTN and the prevalence of PAD in HD patients^306^. There is no evidence that the treatment of HTN has any impact on the onset or regression of PAD in CKD. By extrapolation, it makes sense for patients with aortic aneurysms to be managed with impulse control (reduction in both BP and HR).

Retinal vein occlusion (central artery, central vein) is a complication associated with several risk factors, including HTN. Central retinal artery occlusion is 4.5-fold more frequent in dialytic CKD than in patients with normal renal function. Similarly, central retinal vein occlusion, a more common entity than central artery occlusion, occurs 2.6 times more frequently in dialysis patients^307^. In retrospective studies with multivariate analysis, HTN is independently associated with both complications^308,309^.

Key message:

- To reduce the risk of CV complications in hypertensive dialysis patients, it is recommended to avoid pre-dialysis SBP < 120 or > 160 mmHg (Class IIA/Level B).

## Conclusions

The diagnosis and management of HTN in dialytic CKD are even more complex than for the general population. Different pathophysiological determinants are involved in CKD 5D hypertension, with sodium and volume overload playing a major role. Technologies for this analysis must prove to be both safe and feasible.

There is greater variability in the circadian rhythm of BP, and 44-hour ABPM appears to provide the most accurate diagnostic and prognostic information. However, HBPM is better tolerated and may be a useful substitute in resource-limited settings. RCTs should be conducted in the long-term using these diagnostic and follow-up tools to determine their importance and significance in outcomes.

Both intradialytic and off-dialysis hyper- and hypotension are harmful and should be avoided.

Non-pharmacological interventions, such as dietary sodium restriction, prolonged dialysis vintage, and changing dialysis prescription to improve sodium diffusion, may help in the management of HTN, and have an impact on symptoms, quality of life, and CV complications.

When dry weight is achieved and it is not sufficient for HTN control, antihypertensive pharmacotherapy is recommended. Although meta-analyses reveal a survival benefit from therapy, no study has fully demonstrated the benefit of one class of antihypertensive drugs over another. Large head-to-head comparative RCTs are needed to elucidate the optimal pharmacological treatment for HTN in dialysis, a gap to be filled by researchers worldwide.

Treatment and BP targets should be individualized, considering best practices, cost/benefit, dialysis medication clearance, age, frailty, vulnerability, comorbidities, polypharmacy, as well as the patient’s priorities and preferences.

## Abbreviations

ABPM: ambulatory blood pressure monitoring
ACEI: angiotensin-converting enzyme inhibitors
ADMA: asymmetric dimethylarginine
AF: atrial fibrillation
AMI: acute myocardial infarction
ARBs: angiotensin II receptor blockers
AS: arterial stiffness
BB: beta-blockers
BIA: Bioimpedance analysis
BMI: body mass index
BNP: B-type natriuretic peptide
BP: blood pressure
BSN: Brazilian Society of Nephrology
CAD: coronary artery disease
CAP: central arterial pressure
CCB: calcium channel blockers
CH: controlled hypertension
CKD: chronic kidney disease
CKD 5D: chronic kidney disease with dialysis patient
CNS: central nervous system
CO: cardiac output
CV: cardiovascular
CVA: cerebrovascular accident
CVD: cardiovascular disease
DBP: diastolic blood pressure
DM: *diabetes mellitus*
DW: dry weight
ED: endothelial dysfunction
EPO: human recombinant erythropoietin
ET1: endothelin
GFR: glomerular filtration rate
HBPM: home blood pressure monitoring
HD: hemodialysis
HDF: high-volume online hemodiafiltration
HF: heart failure
HTN: hypertension
IDH: intradialytic hypertension
IDWG: interdialytic weight gain
KDIGO: KIDNEY DISEASES IMPROVING GLOBAL OUTCOMES
LV: left ventricle
LVH: left ventricular hypertrophy
MH: masked hypertension
MRA: mineralocorticoid receptor antagonists
MUH: masked uncontrolled hypertension
NH: nocturnal hypertension
NO: nitric oxide
NT-proBNP: N-terminal pro-B-type natriuretic peptide
OSA: obstructive sleep apnea syndrome
PD: peritoneal dialysis
POAD: peripheral obstructive artery disease
PP: pulse pressure
PRA: plasma renin activity
RAAS: renin-angiotensin-aldosterone system
RCT: randomized controlled trials
RRF: residual renal function
RRT: renal replacement therapy
RV: right ventricle
SBP: systolic blood pressure
SH: sustained hypertension
SHTN: systolic hypertension
SMBP: self-measured blood pressure
SNS: sympathetic nervous system
SUH: sustained uncontrolled hypertension
SVR: systemic vascular resistance
TI: therapeutic inertia
TN: true normotension
UF: ultrafiltration
WCH: white coat hypertension
WCUH: white coat uncontrolled hypertension
