# Supplementary material for: I Brazilian guideline on hypertension in dialysis of the Brazilian Society of Nephrology
Source: J Bras Nefrol. 2025 Feb 24;47(1):e20240033. doi: 10.1590/2175-8239-JBN-2024-0033en (PMC11864789; doi:10.1590/2175-8239-JBN-2024-0033en)
Supplement: Translation

# Translation: I Diretriz Brasileira de hipertensão arterial na diálise da Sociedade Brasileira de Nefrologia

**DOI:** 10.1590/2175-8239-JBN-2024-0033pt

**Authors:** Cibele Isaac Saad Rodrigues, Sebastião Rodrigues Ferreira-Filho, Ana Flávia de Souza Moura, Carlos Eduardo Poli-de-Figueiredo, Dirceu Reis da Silva, Fernanda Salomão Gorayeb Polacchini, Fernando Antônio de Almeida, Maria Eliete Pinheiro, Rodrigo Bezerra, Rogério Baumgratz de Paula, Aldo José Peixoto, Ana Elizabeth Prado Lima Figueiredo, Audes Diógenes Magalhães Feitosa, Carlos Alberto Machado, Celso Amodeo, Décio Mion, Elizabeth Silaid Muxfeldt, Giovanio Vieira da Silva, José Andrade Moura-Neto, José Muniz Pazeli, Leda Daud Lotaif, Luciano F. Drager, Luis Cuadrado Martín, Luiz Aparecido Bortolotto, Marcus Gomes Bastos, Marcus Vinícius Bolívar Malachias, Marcos Vinícius Paiva Cavalcanti Moreira, Maria Eugenia Fernandes Canziani, Roberto Dischinger Miranda, Roberto Jorge da Silva Franco, Roberto Pecoits, Rogerio Andrade Mulinari, Rosilene Motta Elias, Weimar Kunz Sebba Barroso, Wilson Nadruz

**Affiliations:** 1 Sociedade Brasileira de Nefrologia, São Paulo, SP, Brasil.; 2 Sociedade Brasileira de Hipertensão, São Paulo, SP, Brasil.; 3 Pontifícia Universidade Católica de São Paulo, Sorocaba, SP, Brasil.; 4 Universidade Federal de Uberlândia, Uberlândia, MG, Brasil.; 5 Escola Bahiana de Medicina e Saúde Pública, Salvador, BA, Brasil.; 6 Pontifícia Universidade Católica do Rio Grande do Sul, Porto Alegre, RS, Brasil.; 7 Hospital de Clínicas de Porto Alegre, Porto Alegre, RS, Brasil.; 8 Hospital de Base de São José do Rio Preto, São José do Rio Preto, SP, Brasil.; 9 Universidade Federal de Alagoas, Alagoas, AL, Brasil.; 10 Universidade Federal de Pernambuco, Recife, PE, Brasil.; 11 Universidade Federal de Juiz de Fora, Juiz de Fora, MG, Brasil.; 12 Yale University, Yale School of Medicine, New Haven, United States.; 13 Sociedade Brasileira de Cardiologia, São Paulo, SP, Brasil.; 14 Secretaria Municipal de Saúde de Campos do Jordão, Campos do Jordão, SP, Brasil.; 15 Hospital do Coração da Associação Beneficente Síria de São Paulo, São Paulo, SP, Brasil.; 16 Universidade de São Paulo, São Paulo, SP, Brasil.; 17 Universidade Federal do Rio de Janeiro, Rio de Janeiro, RJ, Brasil.; 18 Faculdade de Medicina de Barbacena, Barbacena, MG, Brasil.; 19 Universidade Estadual Paulista, Botucatu, SP, Brasil.; 20 Faculdade de Ciências Médicas de Minas Gerais, Belo Horizonte, MG, Brasil.; 21 Universidade Federal de São Paulo, São Paulo, SP, Brasil.; 22 Pontifícia Universidade Católica do Paraná, Curitiba, PR, Brasil.; 23 Arbor Research Collaborative for Health, Ann Arbor, Estados Unidos.; 24 Universidade Federal do Paraná, Curitiba, PR, Brasil.; 25 Universidade Nove de Julho, São Paulo, SP, Brasil.; 26 Universidade Federal de Goiás, Goiania, GO, Brasil.; 27 Universidade Estadual de Campinas, Campinas, SP, Brasil.

**Keywords:** Hipertensão, Diálise Renal, Diálise Peritoneal, Fatores de Risco, Tratamento Farmacológico, Guias de Prática Clínica Como Assunto, Falência Renal Crônica, Hipertensão Renal, Determinação da Pressão Arterial, Insuficiência Renal Crônica

## Abstract

A hipertensão arterial em pacientes em diálise (HAD) tem alta prevalência, de pelo menos 80% ou mais, e seu manejo na prática do nefrologista ocorre de forma heterogênea e, frequentemente, empírica. Saber definir, conhecer a fisiopatologia, diagnosticar, acompanhar e tratar com mudanças no estilo de vida, e adequar os medicamentos anti-hipertensivos para alcançar a meta de pressão arterial (PA) recomendada, com vistas à redução da morbidade e mortalidade, requerem conhecimentos e abordagens específicos nos contextos da hemodiálise (HD) e da diálise peritoneal (DP). Este documento é a primeira diretriz da Sociedade Brasileira de Nefrologia, elaborada pelos departamentos de Hipertensão e de Diálise, que visa orientar os médicos que prestam assistência em centros de diálise a como manejar pacientes com HAD, de forma integral e individualizada, com base no julgamento crítico das melhores evidências científicas disponíveis e, quando essas são escassas ou indisponíveis, indicar a opinião de especialistas. Os diferentes temas abordados envolvem: a definição (PA pré-HD ≥ 140/90 mmHg e PA pós-HD ≥ 130/80 mmHg), epidemiologia e fisiopatologia; diagnóstico da HAD preferencialmente com medidas da PA fora do ambiente de diálise (PA ≥ 130/80 mmHg); avaliação complementar; metas pressóricas; tratamento não medicamentoso; uso dos anti-hipertensivos mais adequados; situações especiais; e complicações da HAD, predominantemente as cardiovasculares.

## Regras Para Recomendações e Níveis de Evidência^1^

As recomendações foram estratificadas em Classes ou Graus I, IIa, IIb ou III e níveis de evidência, assim descritas:

### Classes (Graus) de Recomendação

Classe I – Condições para as quais há evidências conclusivas, ou, na sua falta, há recomendação de que o procedimento seja seguro e útil/eficaz;

Classe II – Condições para as quais há evidências conflitantes e/ou divergência de opinião sobre segurança e utilidade/ eficácia do procedimento;

Classe IIA – Peso ou evidência/opinião a favor do procedimento. A maioria aprova;

Classe IIB – Segurança e utilidade/eficácia menos bem estabelecida, não havendo predomínio de opiniões a favor;

Classe III – Condições para as quais há evidências e/ou consenso de que o procedimento não é útil/eficaz e, em alguns casos, pode ser prejudicial.

### Níveis de Evidência

Nível A – Dados obtidos a partir de múltiplos estudos randomizados de bom porte, concordantes e/ou de meta-análise robusta de estudos clínicos randomizados;

Nível B – Dados obtidos a partir de meta-análise menos robusta, a partir de um único estudo randomizado ou de estudos não randomizados (observacionais);

Nível C – Dados obtidos de opiniões consensuais de especialistas.

## Introduçao

O objetivo principal desta publicação é trazer ao médico nefrologista, que atua no cuidado de pacientes renais crônicos em diálise, as melhores evidências científicas disponíveis sobre os diferentes aspectos da HA, desde sua fisiopatologia até o tratamento. Trata-se da construção de um documento inédito em língua portuguesa que seja útil na prática clínica diária do nefrologista.

Os departamentos da SBN envolvidos almejam que as recomendações e sugestões aqui expressas tenham repercussão nacional, contribuindo para a melhor abordagem e o tratamento das pessoas que necessitam de terapias dialíticas, com consequentes benefícios na redução da morbidade e mortalidade cardiovascular e renal.

É fato que, mesmo o prestigioso KDIGO (*Kidney Diseases Improving Global Outcomes*), em seus *guidelines* de HA na DRC, não postulou recomendações para o estágio 5D, provavelmente pela falta de estudos clínicos randomizados e controlados e de revisões sistemáticas e meta-análises nessa população que pudessem ancorar recomendações com altos níveis de confiança e qualidade.

Atualmente, no Brasil, segundo o Censo da Sociedade Brasileira de Nefrologia, de 2022, existem 872 centros de diálise, com prevalência estimada de 153.831 pacientes em estágio 5D, sendo 91% em HD. A HA é apontada como causa direta responsável pela DRC 5D em cerca de 34% dos pacientes e está presente como comorbidade em mais de 80% dos pacientes em programas de diálise. Ou seja, existe um enorme problema a ser enfrentado e possivelmente uma inércia terapêutica que pode ser responsável por desfechos piores de morbidade e mortalidade^2^.

## Definição, Epidemiologia e Fisiopatologia da ha no Paciente em Diálise (HAD)

### Definição/Epidemiologia

HAD é definida a partir das observações previamente estabelecidas para a população geral, e os limites sobre o que é normotensão ou hipertensão variam nas diferentes diretrizes^3^. Assim, a medida da PA em pacientes em diálise representa um desafio à padronização. O ideal é que o diagnóstico de HAD seja baseado em avaliações interdialíticas domiciliares com MRPA ou MAPA, com a padronização recomendada^4,5^.

Em termos epidemiológicos, a HA ocorre em mais de 80% dos pacientes em diálise, sendo, frequentemente, mal controlada^6^.

Principais mensagens:

- A HID tem prevalência estimada de 5% a 15% e correlaciona-se com hospitalizações e mortalidade^7^. (Classe I/Nível B);
- Muitos trabalhos mostram a presença de epidemiologia reversa, com curvas em J ou U^8^ (Classe I/Nível B).

Para o diagnóstico de HAD, recomenda-se:

- MRPA: média ≥ 130/80 mmHg, considerando o dia de instalação e mais 6 dias consecutivos (vide protocolo no capítulo 3);
- MAPA em HD: média ≥ 130/80 mmHg em 44h (vide protocolo no capítulo 3);
- MAPA em DP: média ≥ 130/80 mmHg em 24h (vide protocolo no capítulo 3);
- MRPA/MAPA indisponíveis: medidas realizadas em dia não dialítico no meio da semana na HD, ou no consultório na DP;
- HID é definida por aumento ≥ 10 mmHg na PAS durante ou imediatamente após a HD em 4 de 6 sessões. Motiva avaliação adicional^7^.

Principais mensagens:

- As medidas da PA pré, intra e pós-HD são imprecisas para diagnóstico, mas úteis para diagnosticar HID e para o controle hemodinâmico^9^ (Classe I/Nível B).
- O limite diagnóstico pré-HD é > 140/90 mmHg e pós-HD > 130/80 mmHg^10^ (Classe I/Nível B).

### Fisiopatologia da HAD

A fisiopatologia da HAD é complexa e multifatorial, englobando fatores gerais e específicos da DRC^5^ (Figura 1). Entretanto, a maioria dos pacientes com DRC 5D tem como causas principais a HA e o DM, ou seja, trata-se de uma população com HA que pode anteceder em décadas o início da terapia dialítica. Além disso, pacientes com DRC de outras etiologias também apresentam HA, frequentemente secundária à doença renal de base.

**Figura 1:** Fisiopatologia da hipertensão arterial em pacientes sob tratamento dialítico.

Abreviaturas – DRC Grau 5D: doença renal crônica estágio 5 em diálise; AOS: apneia obstrutiva do sono; Na+: sódio; TRS: terapia renal substitutiva; SRAA: sistema renina angiotensina aldosterona; SNS: sistema nervoso simpático; EPO: eritropoietina recombinante humana.

### Relação entre sobrecarga de volume e hipertensão arterial em DRC

O papel da hipervolemia na HAD pode ser evidenciado pela redução da PA associada à intensificação da UF. Em um estudo, a realização de HD diária e de longa duração resultou no controle pressórico em cerca de 90% dos pacientes^11^, sendo observada dissociação entre a obtenção do PS estimado e o controle pressórico, verificado após semanas ou meses do alcance da euvolemia. Esse fenômeno, conhecido como “*lag phenomenum*”, parece se relacionar à remoção adicional de sal, à medida que se intensifica a UF^12^. Resultados semelhantes foram observados na Turquia^13^. Estratégias comuns nesses estudos incluíram a restrição de sal na dieta e a realização de sessões de diálises prolongadas. Estudo randomizado, no qual a intensificação da UF foi obtida sem ampliação do tempo das sessões de HD, confirmou significativa melhora do controle da PA nos pacientes que atingiram o PS estimado, quando comparados aos do grupo controle^14^.

Em pacientes em DP, poucos são os estudos que avaliaram a relação da hipervolemia com a HA^15^. Um deles avaliou pacientes em DP, submetidos à intensificação da UF, com redução do peso corporal em 5%^16^. Após 5 semanas, foram observadas reduções do peso corporal, da água extracelular, do diâmetro inspiratório da veia cava inferior e melhor controle pressórico. A hipervolemia parece exercer papel preponderante na HA em pacientes em DP.

Nas últimas décadas, um novo elemento tem sido associado ao excesso de sódio presente na DRC. Em 2003, Titze e cols.^17^ mostraram retenção de sódio complexado à glicosaminoglicanos na pele e músculos de modelos animais, portanto sem retenção de água. O acúmulo não osmótico de sódio forma um sistema “tampão” de armazenamento de sódio^18^, com recrutamento de macrófagos, indução da expressão do gene da *“tonicity-responsive enhancer-binding protein*” (TonE) e do fator de crescimento derivado do endotélio (VEGF), que induzem à hipertrofia de vasos linfáticos, à liberação de ON e vasodilatação. Contudo, em situações de excesso de sódio ou de incapacidade de sua eliminação, o funcionamento desse sistema “tampão” está comprometido, gerando aumento do sódio na pele e nos músculos, com ativação do sistema imune, inflamação e fibrose^19^. Aumento do conteúdo de sódio subcutâneo, demonstrável por meio de ressonância nuclear magnética, contribui para a HA e para aumento de eventos CV em renais crônicos^20,21^.

Principais mensagens:

- O papel da hipervolemia na HA da DRC estágio 5D pode ser evidenciado pela redução da PA associada à intensificação da UF (Classe I/Nível B). Em pacientes em DP, também parece exercer papel preponderante na HA (Classe I/Nível C).

#### Parâmetros que interferem na pa: sódio/composição da solução de diálise

Pacientes em diálise são habitualmente hipervolêmicos e hipertensos, em especial por sensibilidade exacerbada ao sal, ativação inapropriada do SRAA à ingestão de sódio e natriurese diminuída^22^. O pool corporal de sódio inclui depósitos cutâneos e musculares, particularmente em diabéticos^23^, com remoção parcial pela HD^21^ e impacta no controle pressórico e na HVE^24^. Quando persiste ao final da HD, a HA pode traduzir hipervolemia residual^25^, assim como no intervalo interdialítico^26^.

Concentrações diferentes de sódio na solução de diálise podem relacionar-se com o controle volêmico e da PA^27,28,29,30,31^ e, se superiores às concentrações séricas, resulta em aumento do GPID, sem se correlacionar com variações da PA^27^. Os pacientes submetidos a menor concentração de sódio na solução de diálise tiveram maior ocorrência de hipotensão intradialítica^27,28,29,31^, menor GPID^28,29,30,31^, menor PA^29,30,31^ e uso minimizado de anti-hipertensivos^29^, mas sem efeitos positivos na redução da HVE^28^, nas taxas de internação^27^ e na mortalidade^27^. Estudo em andamento pretende avaliar o efeito de diferentes concentrações de sódio do dialisato em eventos CV e mortalidade em pacientes em HD (RESOLVE, NCT02823821).

Além disso, a natremia pré-HD costuma aproximar-se do nível fisiológico de ajuste individual (*set point*), e pode-se adotar sódio da solução de diálise semelhante a esse nível (diálise isonatrêmica), com base na retirada do sódio em convecção, e evitando balanço positivo^32^.

Principais mensagens:

- Recomenda-se cautela quanto às manobras de aumento ou diminuição do sódio da solução de diálise, pois o sódio alto aumenta o GPID e, ao contrário, sódio baixo diminui o GPID e a PA, mas aumenta o risco de hipotensão intradialítica (Classe I/Nível B).
- Não há indicação de ajustar a concentração de sódio na solução de diálise com objetivo de reduzir internação ou mortalidade (Classe I/Nível B).

#### Taxa de ultrafiltração (UF)

Taxas de UF elevadas associam-se à hipotensão arterial, mas, caso estejam demasiadamente baixas, podem perpetuar a hipervolemia, principal determinante da HA^6^. A relação entre UF e redução da PA é consistente, mas em hipertensos pode sofrer influência de outros fatores envolvidos na fisiopatologia da HA^33^.

Necessária para se evitar hipotensão, a manutenção do volume plasmático durante a HD depende do *refilling* (absorção de fluidos intersticiais e linfáticos na microcirculação), que independe *per se* da adoção de taxas de UF maiores ou não^34^.

Mecanismos compensatórios contrarregulatórios à UF são a taquicardia reflexa e vasoconstrição. Portanto, pacientes com disfunção autonômica e/ou ventricular podem desenvolver hipotensão intradialítica mesmo com taxas baixas de UF^35^. Taxas elevadas de UF associam-se à hipotensão intradialítica, redução da FRR e aumento de mortalidade^35,36,37,38,39^. A adoção de taxa de UF > 13 mL/kg/h^35,36,37,38,39^ pareceu globalmente deletéria e taxas de UF > 10 mL/kg/h^36,37,39^ são prejudiciais, principalmente na presença de disfunção ventricular^35^. Em esquema de HD de 1 a 2 vezes por semana, parecem ser necessárias taxas ainda menores de UF (inferiores a 6 mL/Kg/h)^36^. Se a FRR permanece preservada, a associação de alta taxa de UF com mortalidade é atenuada^36^.

A hipotensão está associada à redução acelerada da FRR, atenuando o efeito positivo desta sobre a mortalidade, tanto em HD como em DP^40^. Recomenda-se aumentar o tempo da sessão de HD ou sua frequência para evitar taxas de UF > 13 mL/kg/h e, ainda assim, manejar hipervolemia^9,41^. Essencialmente, esses limites de taxa de UF podem ser adotados, mas deve-se considerar ainda um conjunto de fatores, incluindo hemodinâmica intradialítica, comorbidades, sintomas e uso de medicamentos^41^.

Principais mensagens:

- Recomenda-se evitar taxas elevadas de UF, ou seja, > 13 mL/kg/h, pois se associa a aumento da mortalidade, hipotensão intradialítica e redução acelerada da FRR.
- Recomenda-se que, em pacientes com disfunção ventricular, o limiar sugerido é ainda menor (até 10 mL/kg/h) (Classe I/Nível B).

#### Ativação do sistema renina angiotensina aldosterona (SRAA)

Os rins são capazes de sintetizar todos os componentes do SRAA, inclusive em pacientes em diálise^42^, sendo que neles a APR está inapropriadamente elevada em relação à hipervolemia. Esses dados são reforçados pelo aumento da APR após as sessões de diálise, indicando que néfrons remanescentes são capazes de perceber as variações de sódio e aumentar a atividade do SRAA. A angiotensina II exerce efeitos deletérios ao estimular a produção de aldosterona e a retenção de sódio ao causar lesão endotelial e estimular o SNS^42,43,44^. A administração de lisinopril a pacientes em HD resultou em melhor controle da PA, avaliada por meio da MAPA de 44 horas, quando comparados ao grupo controle^45^.

Além da angiotensina II, tem sido evidenciada a ação vasculotóxica da aldosterona, principalmente na presença de sal. Tal achado sugere que a aldosterona possa desempenhar um papel permissivo na toxicidade do sódio no endotélio, tornando-se assim um fator de risco para complicações CV^46^.

Em concordância, o bloqueio dos receptores mineralocorticoides com espironolactona resultou em melhor controle pressórico e em redução de mortalidade CV em pacientes em HD^47^. Assim, a aldosterona contribui para o agravamento da HA ao exercer efeitos genômicos e não genômicos, com indução de inflamação e toxicidade vascular, na presença de excesso de sal.

Principais mensagens:

- Recomenda-se o bloqueio do SRAA devido à sua ativação inapropriada, a despeito da hipervolemia, o que contribui de modo significativo para a gênese da HA e para o aumento do risco CV em pacientes com DRC (Classe I/Nível B).

#### Ativação do Sistema Nrvoso Simpático (SNS)

Ativação do SNS na DRC constitui um dos principais mecanismos relacionados à fisiopatologia da HA. Entre os fatores responsáveis pela hiperatividade do SNS na DRC, estão a redução da biodisponibilidade de ON, DE, toxicidade urêmica e inflamação, além de elevada prevalência de AOS^48,49,50^.

Aumento das concentrações de norepinefrina plasmática e da atividade simpática, avaliada por meio da atividade simpática muscular (MSNA), evidenciam a importância do SNS na fisiopatologia da HA em DRC. Aferentes simpáticos renais que inervam as artérias renais e eferentes do SNC estimulados por receptores B1, causam vasoconstrição arteriolar renal e ativação do SRAA, com consequente aumento da resistência vascular renal e retenção de sódio^51^. Pacientes com DRC apresentam deficiência de renalase, enzima produzida pelos rins e responsável pela metabolização de catecolaminas^52^.

A hiperatividade do SNS também contribui para a mortalidade CV, estando associada a doença aterosclerótica, HVE e presença de arritmias cardíacas, responsáveis por cerca de 25% dos óbitos de pacientes em diálise^53^. A hiperatividade simpática é confirmada pelo melhor controle pressórico após nefrectomia de rins nativos em pacientes com DRC estágio 5D^54^ e pela denervação renal em pacientes em diferentes estágios da DRC^55^ e em HD^56^. Finalmente, a administração de BB em pacientes em diálise reduz a progressão da DRC e da mortalidade^57,58^.

Principais mensagens:

- Recomenda-se o bloqueio do SNS para o tratamento da HA, pois a hiperatividade simpática é importante mecanismo fisiopatológico na HA e na mortalidade em DRC (Classe I/Nível A).

#### Disfunção endotelial (DE)

É razoável afirmar que a DE associada à HA em pacientes em HD ou DP precede em décadas o diagnóstico da DRC, considerando-se que a maioria dos pacientes com DRC tem DM e/ou HA, ou é idoso e apresenta diversos fatores de risco CV. Estudo em ratos submetidos à nefrectomia 5/6 documentaram a redução da atividade da ON-sintase endotelial, com consequente menor disponibilidade de ON e elevação da PA^59^. A redução da oferta de ON tem sido atribuída às alterações no metabolismo das pteridinas, que são compostos aromáticos que agem como cofatores em diversos processos inflamatórios e imunológicos^60^. Em pacientes com DRC estágio 5D, a redução da relação entre BH4/BH2, compostos pertencentes ao grupo das pteridinas, se associou à redução da disponibilidade de NO endotelial, ao processo de inflamação/desnutrição e à DCV^61^. Também o estresse oxidativo presente na DR^62^ e, particularmente, o aumento dos níveis plasmáticos de dimetil-arginina-assimétrica (DMAA) interferem na produção de NO e estão associados à HVE e mortalidade CV em pacientes em HD^63,64,65^. A DMAA é inibidor endógeno da síntese de NO, que se acumula devido às reduções da TFG e da metabolização intracelular^65^.

Pacientes com DRC no estágio 5D apresentam elevação dos níveis de ET1, o que contribui para o agravamento da HA^66^ e desempenha papel significativo na ocorrência de HID^67^.

Principais mensagens:

- A disfunção endotelial previamente existente na maioria dos pacientes com DRC torna-se ainda mais grave à medida que ocorre redução da TFG, contribuindo desse modo para a fisiopatologia da HA e para a ocorrência de eventos CV e morte (Classe II/Nível B).

#### Aumento da Rigidez Arterial (RA)

A RA causa aumento nas pressões arteriais periféricas e centrais, tais como PAS, PP e da massa do VE, assim como reduções na PAD e perfusão coronariana. A RA também pode ser considerada um fator independente de mortalidade CV e progressão da DRC.

Os mecanismos envolvidos na RA dos pacientes com DRC ainda não estão completamente definidos, mas incluem calcificação arterial, sobrecarga crônica de volume, estresse mecânico pela HA, microinflamação crônica, hiperatividade simpática e do SRAA, acúmulo dos produtos da degradação da glicose, peroxidação lipídica e anormalidades do sistema de ON. Mesmo uma leve redução na TFG é fator de risco para o desenvolvimento da RA. A UF na HD não é capaz de reverter/diminuir significativamente a RA^68,69,70,71^.

A RA é multifatorial e alguns deles são exclusivos para DRC: a) níveis elevados de fósforo, que ativa genes relacionados ao fenótipo osteoblástico nas células musculares lisas, levando à calcificação arterial^72^; b) a desnutrição proteico-energética, que é comum na DRC e causa aumento na RA^73^; c) elevação do DMAA; d) aumento do FGF 23; e) redução de magnésio^74,75,76^. Baixas concentrações séricas de fetuína-A estão associadas à calcificação vascular na DRC^77^.

#### Apneia obstrutiva do sono na HAD

AOS é definida como obstrução parcial ou completa das vias áreas superiores durante o sono, promovendo despertares e hipoxemia intermitente. O diagnóstico e a gravidade da AOS é baseada no índice apneia-hipopneia (IAH) obtida pela polissonografia ou poligrafia: até 4,9 = ausência de AOS; 5,0 a 14,9 = AOS leve; 15 a 29,9 = AOS moderada; e ≥ 30 eventos por hora = AOS grave^78^.

A AOS é muito prevalente na população dialítica, atingindo 50% a 70% dos pacientes. Quando associada a HA, apresenta mecanismos fisiopatológicos que se amplificam, destacando-se entre eles a hipervolemia^79,80^.

O quadro clínico é pouco específico e a prevalência de ronco e sonolência diurna são menos relatadas na DRC 5D^81^. Indicação de um exame objetivo do sono deve ser considerada de maneira mais liberal nesses pacientes*.* AOS grave pode estar relacionada à HA resistente e refratária^82^.

Identificada AOS moderada ou grave, independentemente do comportamento da PA, o tratamento deve ser individualizado. A indicação de pressão positiva nas vias áreas durante o sono (CPAP) pode não ser a primeira escolha em pacientes em diálise. Para diminuir a hipervolemia nesse subgrupo, técnicas de otimização da UF, como HD longa noturna ou DP com máquinas cicladoras, mostraram-se efetivas em ensaios clínicos pequenos^78^.

Principais mensagens:

- O quadro clínico da AOS em pacientes com DRC 5D é pouco específico. A indicação de exame objetivo do sono (polissonografia ou poligrafia) deve ser considerada de maneira mais liberal e individualizada, assim como o uso de CPAP (Classe IIB/Nível C).

#### Medicamentos que interferem no controle pressórico

Aumento da PA é uma complicação bem reconhecida da terapia com EPO em pacientes em HD^83^. Aproximadamente 30% dos pacientes desenvolvem HA ou necessitam de ajuste dos medicamentos anti-hipertensivos, após algumas semanas ou meses. A fisiopatologia da HA por uso de EPO parece ser independente do seu efeito sobre a massa e viscosidade das hemácias^84^. Os mecanismos mais prováveis envolvem aumento na produção ou resposta aumentada à ET-1, aumento acentuado na resposta da PA à infusão de angiotensina II e hipersensibilidade à noradrenalina^83^. Há relatos da associação entre anormalidades no ritmo circadiano da PA e uso de EPO na MAPA^3^. A prevenção da hipertensão induzida por EPO é um desafio clínico com várias estratégias de manejo possíveis listadas abaixo como recomendações^83,84^.

Por fim, deve-se evitar o uso de medicações que notoriamente elevam a PA, sobre as quais as Diretrizes Brasileiras de Hipertensão já fazem ampla menção^4^.

Principais mensagens:

Na HA induzida por EPO refratária ao manejo dos anti-hipertensivos recomenda-se:

- Atenção ao peso seco (Classe IIA/Nível B);
- Preferência pela via subcutânea da EPO (Classe IIA/Nível B);
- Reduzir o alvo da hemoglobina (Classe IIA/Nível C);
- Iniciar com baixa dose de EPO, aumentar lentamente e, em casos extremos, suspender o uso (Classe IIA/Nível B);
- Evitar o uso de medicações que notoriamente elevam a PA (Classe IIA/Nível B).

#### Inércia da equipe assistencial

Inércia clínica ou terapêutica (IT) consiste na falha dos profissionais de saúde em iniciar, intensificar ou encerrar tratamentos quando indicado, e inclui medidas medicamentosas ou não^85^. A IT é observada em cerca de 2/3 das visitas de hipertensos^86^, e na literatura em diálise encontram-se evidências associadas à falência em introduzir ou intensificar a terapia anti-hipertensiva ou ajuste da volemia. Em análise retrospectiva de pacientes com DRC e HA não controlada, parece ter ocorrido IT em aproximadamente 44% das ocasiões, a julgar pela não identificação de justificativa para a falha de decisão médica^87^.

Quanto ao perfil psicológico do médico mais propenso à IT, parece predominar a preferência pela aparente segurança no curto prazo com a inação^88^. O enfrentamento desse comportamento envolve o encorajamento continuado de condutas proativas destinadas a atingir as metas terapêuticas, dentro de política de melhoria da qualidade assistencial^89^.

IT é descrita em vários cenários de prevenção CV^89^, como no manejo de pacientes em diálise com DM^90^ ou que sejam idosos em uso de polifarmácia^91^. Fatores implicáveis incluem: incerteza clínica sobre a medida da PA, baixa adesão medicamentosa e hipotensão diastólica e/ou ortostática^87^.

Examinando o emprego de medicações potencialmente inapropriadas (alfa-agonistas de ação central ou alfa-bloqueadores), o risco de IT em idosos em diálise foi maior em negros, com polifarmácia e sem limitações funcionais, mas não ocorreu maior número de hospitalizações ou mortalidade naqueles que mantiveram esses medicamentos^87^.

Entre pacientes hipertensos, houve êxito com o emprego de educação médica estruturada e de um sistema de *feedback* regular, com maior controle da HA, mas sem melhora em desfechos CV^92^.

Recomendações existentes na literatura indicam que a educação e participação dos pacientes no manejo dos seus riscos CV reduzem a IT^88,93^.

Principais mensagens:

- Recomenda-se, para reduzir a inércia terapêutica, que os protocolos para a obtenção de metas clínicas do controle da HA devam envolver pacientes, cuidadores, médicos e equipe multiprofissional (Classe I/ Nível B).

#### Má adesão do tratamento anti-hipertensivo

Adesão pode ser definida como o grau em que uma pessoa corresponde às recomendações do prestador de assistência à saúde^94^. Baixa adesão ao tratamento está associada a desfechos clínicos desfavoráveis e é comum em pacientes com DRC 5D^95^.

Em relação ao controle da PA em HD ou DP, a má-adesão pode trazer uma série de dificuldades para o manejo da HA, visto que a não implementação de medidas comportamentais, como a redução do consumo de sal e a limitação do ganho de peso no período interdialítico, aliado à falha no uso dos medicamentos anti-hipertensivos prescritos, seja por esquemas posológicos complexos, efeitos colaterais associados e, particularmente em paciente em HD, o receio de episódios de hipotensão intradialíticas, possam inviabilizar o alcance das metas terapêuticas.

Quanto às estratégias para melhorar a adesão, os estudos nessa população são escassos. De forma geral, recomendam-se as seguintes estratégias:

1. Redução do consumo de sal/limitação do ganho de peso interdialítico: intervenções estruturadas baseadas em educação continuada do paciente, realizadas por equipe multiprofissional, podem melhorar essas variáveis^96^.
2. Medida da PA no período interdialítico, por meio da MRPA, promovem maior adesão aos anti-hipertensivos, impactando diretamente no controle da PA^97^.
3. Tratamento medicamentoso: escolha de medicamentos com menor perfil de eventos adversos e melhor comodidade posológica^4^.
4. Nos pacientes em HD, o horário de tomada dos medicamentos deve ser ajustado de acordo com a sessão, não suspendendo os anti-hipertensivos, particularmente nos pacientes com tendência a hipertensão intradialítica^98^.

Principais mensagens:

São estratégias para melhorar a adesão ao tratamento anti-hipertensivo em pacientes em HD:

- Educação continuada visando à limitação do consumo de sal e do ganho de peso interdialítico (Classe IIA/Nível B).
- Medida domiciliar ou ambulatorial da PA no período interdialítico (Classe IIA/Nível B).
- Uso de anti-hipertensivos, preferencialmente em dose única diária (Classe I/ Nível A).

## Diagnóstico de Hipertensão em Pacientes Submetidos à Diálise Peritoneal (DP) e Hemodiálise (HD)

As medidas da PA relacionadas à sessão de HD são insuficientes para o diagnóstico de HA e apresentam pobre valor prognóstico. Estudos observacionais relatam associação em forma de “U” entre a PA peridialítica e mortalidade. Em contraste, a PA fora da unidade de diálise apresenta associação linear e direta com mortalidade^99,100^. O comportamento da PA no paciente em diálise está diretamente relacionado ao seu estado volêmico, de modo que a medida antes da HD superestima a PA e após a HD subestima a PA^101^. Oposto a essa esperada queda da PA durante a HD, 7–30% desses pacientes apresentam elevação da PA nesse período^102^. Tudo isso contribui para uma variabilidade e inconsistência das medidas da PA no período peridialítico. Em contrapartida, crescem as evidências da superioridade das medidas fora da unidade de HD.

### Medida da Pressão Arterial Relacionada à Sessão de Hd

A aferição da PA relacionada à sessão de HD pode ser avaliada a partir de três medidas: PA pré-dialítica, PA intradialítica e PA pós-dialítica. A determinação da quantidade de medidas a serem realizadas no período intradialítico dependerá do julgamento clínico do nefrologista. Para essa decisão, serão considerados os valores de PA pré-diálise, o histórico de episódios de hipotensão ou hipertensão intradialítica, a necessidade de taxas de ultrafiltração elevadas, pacientes em processo de ajuste do peso seco e o estado clínico geral do paciente.

Principais mensagens:

- A PA durante a sessão de HD apresenta baixo valor prognóstico (Classe I/Nível B).
- Recomenda-se que durante a sessão de HD sejam realizadas, no mínimo, a medida de PA a cada hora (Classe IIA/ Nível C).
- De acordo com o *Kidney Disease Outcomes Quality Initiative* (KDOQI), nos pacientes em HD, recomenda-se que a HA deve ser diagnosticada como PA pré-dialítica > 140/90 mmHg ou PA pós-dialítica > 130/80 mmHg^10^ (Classe IIA/Nível B).

Contudo, são realizadas em circunstâncias que fogem ao preconizado para a medida adequada da PA, entre elas: ansiedade pelo início do tratamento, expectativa pela dor da punção da fístula arteriovenosa^103^, interrupção da medicação anti-hipertensiva no dia da HD (em alguns pacientes), depuração das medicações anti-hipertensivas, uso de medicações estimulantes da eritropoiese, efeito do avental-branco e do mascaramento, alimentação pré ou intradialítica, validação dos aparelhos oscilométricos utilizados nas máquinas de HD, e número elevado de pacientes a serem avaliados pelo mesmo profissional de saúde em curto intervalo de tempo.

Diversos estudos foram realizados com o intuito de avaliar a acurácia diagnóstica das medidas da PA peridialítica, bem como sua representatividade da PA no período interdialítico^101,104,105^. O padrão encontrado na maioria dos pacientes é uma tendência à redução da PA pós-diálise em relação à PA pré-diálise. Isso pode ser explicado pela resposta hemodinâmica à UF durante a diálise e, em geral, a magnitude dessa redução está associada ao volume ultrafiltrado. Do mesmo modo, estudos sugerem que o GPID tem influência direta na elevação da PA pré-diálise^105^. A execução da técnica correta da medida da PA na unidade de HD é um desafio e, por essa razão, a técnica inadequada já foi responsabilizada pelo mau desempenho da PA pré e pós-HD para diagnosticar e/ou avaliar prognóstico da HA nesses pacientes. No entanto, um estudo mostrou que, mesmo com correta execução, esse tipo de medida não apresenta significado prognóstico^104^. Algumas condições são inerentes ao ambiente de diálise, e realizar as medidas da PA pré e pós-diálise em local discriminado, que preserve os critérios necessários para a medida correta da PA, pode não ser ajustável à logística de uma sessão de HD. Assim, as aferições da PA durante a sessão de HD não devem ser utilizadas para diagnóstico de HA ou definição de ajustes no esquema anti-hipertensivo do paciente^101^.

As medidas da PA peridialíticas são estimativas imprecisas da PA, limitando sua capacidade de fornecer associações prognósticas claras e diretas, mesmo quando tal associação realmente existe.

### Hipertensão Intradialítica (HID)

Apesar da retirada de líquidos com a UF, existe um subconjunto de pacientes que apresenta elevação da PA durante e/ou após a sessão de HD, em comparação aos níveis pressóricos pré-HD. Esses pacientes são classificados como portadores de HID^5^. A elevação da PAS ≥ 10 mmHg durante ou após a sessão de HD em relação aos níveis pré-diálise em 4 de 6 sessões é comumente usada para caracterizar o paciente com HID^106^. No entanto, Singh e cols. relataram que quaisquer aumentos observados na PAS estão relacionados a maior risco de eventos CV fatais^107^.

É importante notar que a HID não é detectada em todas as sessões de HD para um mesmo paciente. Por outro lado, existe maior correlação entre os valores pressóricos detectados nos primeiros 90 minutos de HD e os valores aferidos imediatamente após a sessão de HD. No Brasil, uma análise unicêntrica mostrou que 11% dos pacientes tinham aumento da PAS ≥ 10 mmHg em mais de 50% das sessões de HD^108^. Os mecanismos fisiopatológicos que justificam essa condição incluem a hipervolemia, o balanço positivo de sódio, a hiperatividade do SRAA e do SNS, a DE e a depuração maior ou menor das medicações anti-hipertensivas^109^.

Os principais mecanismos envolvidos na HA intradialítica estão destacados no Quadro 1.

**Quadro 1:** Mecanismos fisiopatológicos da hipertensão intradialítica (HID)

Hipervolemia	O excesso de fluido é uma das mais importantes causas de elevação dos níveis pressóricos em pacientes com HID. Os achados indicam que o aumento paradoxal dos valores pressóricos que ocorre em alguns pacientes durante a UF pode ser causado por aumento do DC na presença de hipervolemia e dilatação de câmaras cardíacas, sugerindo a intensificação da UF como forma de tratamento da HID.
Ativação do sistema renina-angiotensina-aldosterona	O estímulo do SRAA é causado pela hipovolemia induzida pela UF.
Hiperatividade do sistema nervoso simpático	Indivíduos portadores de DRC usualmente apresentam hiperatividade simpática e que pode ser reduzida com o aumento da frequência das sessões de HD110.
Controle do sódio	O balanço positivo do sódio é um dos principais mecanismos de sobrecarga de líquido extracelular e hipertensão em pacientes em diálise. A remoção satisfatória do sódio pode ser atingida por meio de adequação da taxa de UF e da concentração do sódio na solução de diálise.
Disfunção endotelial	Em resposta à UF, aos estímulos mecânicos e hormonais, as células endoteliais sintetizam e liberam fatores humorais que contribuem para a homeostase da PA. Pacientes com HID apresentavam acréscimo significativo nos níveis plasmáticos de endotelina-1 e decréscimo nos níveis de ON, comparado aos controles109.
Depuração dialítica das medicações anti-hipertensivas	As medicações anti-hipertensivas apresentam diferentes suscetibilidades à filtração pela membrana do dialisador.

Abreviaturas – HID: hipertensão intradialítica; DC: débito cardíaco; UF: ultrafiltração; PS: peso seco; SRAA: sistema renina angiotensina aldosterona; DRC: doença renal crônica; ON: óxido nítrico.

Várias estratégias têm sido propostas para tratamento da HID, tais como^111^:

- Otimização do tratamento anti-hipertensivo: certificar-se do uso de medicamentos apropriados, buscar o peso seco ideal, verificar a adesão ao tratamento farmacológico e não farmacológico, além de estender o tempo e/ou a frequência das sessões de HD;
- Considerar o uso de BB com atividade alfa bloqueadora;
- Usar anti-hipertensivos de curta duração antes do início da sessão de HD;
- Escolha de anti-hipertensivos menos dialisáveis;
- Considerar reduzir a concentração do sódio da solução de diálise;
- Evitar concentração de cálcio muito elevada na solução de diálise;
- Considerar a elevação da temperatura do dialisato. Banhos muito frios são favoráveis ao surgimento de HID;
- Considerar administrar os agentes estimulantes da eritropoiese pela via subcutânea.

### Medidas da Pressão Arterial Fora da Unidade de Hemodiálise

Essas medidas se mostraram melhor parâmetro para avaliar alterações na PA relacionadas à redução do PS, além de estar mais associadas a lesões de órgãos-alvo e eventos CV. Nesses pacientes as prevalências de HA noturna (*risers*) e do padrão *non-dipper* costumam ser superiores às da população hipertensa geral^112,113^.

#### Automedida da pressão arterial (AMPA) no paciente em diálise

Não existem evidências conclusivas que validem protocolos específicos (número de medidas e horários de realização) e nem valores de normalidade para esse método^114^. Entretanto, a AMPA deve seguir as mesmas recomendações e cuidados da medida da PA realizada em consultório.

Principais mensagens:

- AMPA não é recomendada como rotina para o diagnóstico e manejo da HA em pacientes em HD ou DP, devendo apenas ser utilizada como método de triagem (Classe I, Nível C).

#### Monitorização Ambulatorial da Pressão Arterial (MAPA) e Monitorização Residencial da Pressão Arterial (MRPA)

Nos pacientes em HD, a MAPA e MRPA demonstraram melhor correlação com lesão de órgão-alvo, eventos CV e morte por todas as causas^100,101,104^. Ambas apresentam boa correlação entre si, como relatado no estudo DRIP, no qual as alterações da PA avaliadas pela MRPA, depois de 4 e 8 semanas de ajuste do PS, foram associadas às alterações da PA avaliada pela MAPA de 44h^105^. Outro benefício mostrado em estudo com pacientes em HD foi a constatação de que uma estratégia de ajuste das medicações anti-hipertensivas baseada na MRPA mostrou ser mais eficaz no controle da PA do que uma estratégia baseada na PAS pré-HD^97^.

A MAPA de 44h fornece informações prognósticas em pacientes em HD, além de ser o único método capaz de avaliar o comportamento da PA no sono. Assim como na população geral, a relação vigília/sono é um preditor significativo de desfechos clínicos em pacientes em diálise. Portanto, a ausência de descenso da PA durante o sono (< 10% da média da PA no sono em relação à vigília) ou o descenso reverso (elevação da PA no sono em relação à vigília), associa-se ao risco de mortalidade geral e CV^115,116^. Entretanto, a MAPA não é um exame de execução prática e apresenta baixa aceitação nessa população, em parte por causa da prevalente frequência de distúrbios do sono e de prurido associados à DRC^78,117^. Por outro lado, a MRPA, além de apresentar boa correlação com prognóstico, apresenta baixo custo e melhor tolerabilidade^118^. As vantagens dos métodos estão listadas no Quadro 2 e os protocolos recomendados para sua execução estão apresentados nos Quadros 3 e 4 (Classe IIA/Nível C).

**Quadro 2:** Vantagens da monitorização ambulatorial da pressão arterial (MAPA) e da monitorização residencial da pressão arterial (MRPA)

Vantagens comuns da MAPA e MRPA	
• Caracterização da hipertensão do avental branco	
• Caracterização da hipertensão mascarada	
• Identificação da hipertensão arterial resistente verdadeira	
• Maior adesão ao diagnóstico e ao tratamento da hipertensão arterial	
• Melhor eficácia no controle da pressão arterial	
• Boa reprodutibilidade	
• Melhor correlação com lesões de órgãos-alvo e eventos CV do que a PA medida na HD	
**Vantagens MAPA**	**Vantagens MRPA**
• Considerada o padrão-ouro na avaliação da PA	• Avaliação do maior número de medidas da PA e em maior número de dias
• Avaliação da PA durante o sono e atividades diárias	• Envolve o paciente no seu autocuidado (medição da PA)
• Avaliação da elevação rápida da PA matinal	• Apresenta baixo custo
• Avalia o controle da PA nas 24h	• Apresenta boa aceitação pelos pacientes

Abreviaturas – MAPA: monitorização ambulatorial de pressão arterial; MPRA: monitorização residencial da pressão arterial; CV: cardiovasculares; PA: pressão arterial; HD: hemodiálise.

**Quadro 3:** Protocolo para realização da monitorização ambulatorial da pressão arterial (MAPA) nos pacientes em diálise, segundo as *Diretrizes brasileiras de medidas da pressão arterial dentro e fora do consultório* – 2023^119^

Monitorização Ambulatorial da Pressão Arterial (MAPA)	
Nos pacientes que fazem HD três vezes por semana, a MAPA de 24h não contempla a avaliação da PA ao longo do ciclo interdialítico. Recomenda-se a realização de MAPA de 44^5^. Vale ressaltar que o manguito **não** deve ser instalado no braço da FAV.
**Período de verificação:**
• 44h (se o *software* utilizado não contempla o protocolo de 44h, sugerimos a realização de dois exames de 22h consecutivos);
**Número de medidas:** no mínimo 48 medidas válidas na MAPA de 44h (32 medidas válidas na vigília e 16 durante o período de sono) e 24 medidas válidas na MAPA de 24h (16 medidas válidas na vigília e 8 durante o período de sono).
• **Dia de instalação:** na HD deve ser instalada após a sessão de diálise do meio da semana e retirada imediatamente após a sessão seguinte.
**Laudo da MRPA:** no laudo deverá conter a data e o horário de início e término do exame (adicionar em qual período interdialítico foi realizado), número e porcentagem das medidas realizadas e das efetivamente válidas, as médias de PAS e PAD (nas 24h, vigília e sono), o comportamento da PA entre vigília e sono, episódios de hipotensão e/ou picos de PA, se houve correlação entre atividades, sintomas e medicamentos, e na conclusão especificar se o comportamento da PA foi considerado normal ou alterado (é considerado alterado quando as médias forem ≥ 130 mmHg e/ou ≥ 80 mmHg, Tabela 1).

Abreviaturas – MAPA: monitorização ambulatorial da pressão arterial; HD: hemodiálise; PA: pressão arterial; h: horas; FAV: fístula arteriovenosa; MRPA: monitorização residencial da pressão arterial; PAS: pressão arterial sistólica; PAD: pressão arterial diastólica.

**Tabela 1:** Valores de pressão arterial considerados anormais nas medidas pela MAPA (monitorização ambulatorial da pressão arterial) nas 24 horas, vigília e durante o sono e na MRPA (monitorização residencial da pressão arterial) para definição de diagnósticos

	PAS (mmHg)			PAD (mmHg)	
MAPA 44h	≥ 130		e/ou	≥ 80	
MAPA Vigília	≥ 135		e/ou	≥ 85	
MAPA Sono	≥ 120		e/ou	≥ 70	
MRPA	≥ 130		e/ou	≥ 80	

Abreviaturas – MAPA: monitorização arterial da pressão arterial; MRPA: monitorização residencial da pressão arterial; PAS: pressão arterial sistólica; PAD: pressão arterial diastólica.

**Quadro 4:** Protocolo para realização da monitorização residencial da pressão arterial (MRPA) nos pacientes em diálise, segundo as *Diretrizes brasileiras de medidas da pressão arterial dentro e fora do consultório* – 2023^119^

Monitorização Residencial da Pressão Arterial (MRPA)	
Período de verificação: • Dia da instalação + 6 dias consecutivos. • Na DP ou HD diária: dia da instalação + 6 (seis) dias consecutivos ao longo de uma semana.
**Número de medidas:** idealmente, 36 medidas (no mínimo 18 medidas válidas e com medidas em todos os dias, com representação dos períodos da manhã e da noite).
**Dia 0 ou dia de instalação:** para HD e DP são realizadas medidas no consultório ou clínica de HD, nunca no braço da FAV, idealmente três medidas (utilizando-se a média das duas últimas para o cálculo de reação de alarme/mascaramento) e medidas à noite em domicílio, para diminuir um possível efeito do avental branco. As medidas realizadas no consultório ou no domicílio no dia de instalação devem ser excluídas do cálculo da média da MRPA.
**Dias da MRPA:** no domicílio são realizadas medidas de PA por mais 6 dias. O paciente deverá fazer 3 medidas pela manhã e 3 medidas ao entardecer ou à noite. Sempre nos pacientes que realizam HD convencional (duas a três vezes semanais), as medidas realizadas nos dias de diálise devem ser excluídas do cálculo da média da MRPA. Nos casos de HD diária e DP, todas as medidas serão consideradas para o cálculo da média.
**Laudo da MRPA:** deve conter o motivo da solicitação do exame; o número de dias de medidas efetivas, horário e número das medidas de cada dia; a qualidade do procedimento; as médias de PA (total, diárias e as dos períodos da manhã e do fim de tarde/noite), se houve reação de alarme ou mascaramento e se o resultado foi considerado normal ou não (considerado exame anormal quando as médias forem ≥ 130 mmHg e/ou ≥ 80 mmHg, Tabela 1).

Abreviaturas – MRPA: monitorização residencial da pressão arterial; HD: hemodiálise; DP: diálise peritoneal; PA: pressão arterial; FAV: fístula arteriovenosa.

Principais mensagens:

- Mensagem: MAPA e MRPA apresentam melhor correlação com lesões em órgãos-alvo, eventos CV e morte por todas as causas quando comparadas com as medidas peridialíticas (Classe I/Nível B).
- MAPA e MRPA apresentam boa correlação entre si em HD e DP (Classe I/Nível B).
- Recomenda-se a MAPA de 44h como método padrão ouro para o diagnóstico de HA nos pacientes em HD e DP^113^ (Classe I, Nível B).
- Recomenda-se a MRPA (dia de instalação e mais 6 dias) como alternativa, quando a MAPA estiver indisponível (Classe I, Nível C).
- Recomenda-se para DP e HD diária seguir as recomendações para a população hipertensa em geral, para MRPA e MAPA de 24h (Classe I/Nível C).

Em relação à diálise peritoneal (DP), por ser uma modalidade dialítica domiciliar, sugere-se que a MRPA deva ser amplamente aplicada. Em sua última atualização, a Sociedade Internacional de Diálise Peritoneal recomenda que, além da medida da PA na visita clínica, também seja realizada medida da PA domiciliar semanalmente^120^. Porém, por causa da escassez de evidências, estudos prospectivos são necessários para elucidar o real significado prognóstico das medidas de PA fora do consultório na DP.

#### Fenótipos

Levando-se em consideração a PA de consultório e a fora do consultório, a PA apresenta 9 tipos possíveis de comportamento: Normotensão Verdadeira (NV), Hipertensão Controlada (HC), Hipertensão Sustentada (HS), Hipertensão Sustentada Não Controlada (HSNC), Hipertensão do Avental Branco (HAB), Hipertensão do Avental Branco Não Controlada (HABNC), Hipertensão Mascarada (HM), Hipertensão Mascarada Não Controlada (HMNC) e Hipertensão Noturna (HN).

A HAB refere-se à condição em que a PA é anormal no consultório, mas é normal quando medida pela MAPA ou MRPA. A HM refere-se a pacientes nos quais a PA é normal no consultório, mas é anormal quando medida pela MRPA ou MAPA. Nos pacientes que utilizam medicações anti-hipertensivas, deve-se adicionar o termo “não controlada”. A NV é quando as medidas da PA no consultório e fora dele são normais e o paciente não utiliza medicação anti-hipertensiva; caso utilize, é definida como HC. A HS é usada quando ambas são anormais na ausência de anti-hipertensivos. Se o paciente utiliza medicação anti-hipertensiva, é definida como HSNC. A HN é definida quando a medida da média da PA pela MAPA está alterada no sono e nas 24h ou 44h, porém normal na vigília. A prevalência dos fenótipos varia consideravelmente de acordo com o método de medição da PA e definição de HA. Em um dos poucos estudos que avaliaram a prevalência dos fenótipos entre os pacientes em HD, foram utilizados os valores de anormalidade da MAPA de 44h ≥ 135/85 mmHg e de ≥ 140/80 mmHg para a mediana da PA pré-HD e pós-HD de seis sessões de meio da semana consecutivas. As prevalências encontradas podem ser visualizadas na Figura 2 ^121^.

**Figura 2:** Prevalência dos fenótipos de hipertensão arterial em pacientes com doença renal crônica em hemodiálise.

Abreviaturas – PA: pressão arterial; MRPA: medida residencial da pressão arterial; MAPA: medida ambulatorial da pressão arterial.

Há carência de estudos que avaliem o prognóstico dos fenótipos em pacientes dialíticos. Entretanto, entre pacientes com DRC não dialítica, o risco de morbidade e mortalidade CV não aumentou naqueles com HABNC, mas aumentou naqueles com HMNC e HSNC^122^.

Principais mensagens:

- São necessários mais estudos para avaliar os fenótipos de HA na população em todas as modalidades dialíticas.

## Avaliações Complementares de Hipertensão e Risco Cardiovascular na Diálise Peritoneal (DP) e Hemodiálise (HD)

### Cálculo do Risco Cardiovascular na Doença Renal Crônica Dialítica

Se por um lado a DRC é um forte preditor independente de doença CV^123 124,125^, temos que a própria doença CV é a principal causa de morte em doentes renais crônicos, traduzindo a importância da identificação dos pacientes com alto risco de eventos e morte CV^126,127^. Na população geral, existem métodos capazes de prever a probabilidade de ocorrência em 10 anos de um evento CV, destacando-se o escore de Framingham e o algoritmo de risco Atherosclerotic Cardiovascular Disease (ASCVD), que consideram apenas fatores tradicionais de risco CV^128,129^. Nos pacientes com DRC, esses escores apresentam menor acurácia, provavelmente pela existência de fatores de risco não tradicionais relacionados à perda de função renal e ao tratamento dialítico^130,131,132,133^. Coerentemente com essa visão, os fatores tradicionais de risco são predominantes nos estágios iniciais da DRC, ao passo que aqueles não tradicionais ganham relevância com a progressão da DRC^130^.

Modelos de cálculos de risco valorizando fatores específicos para pacientes em diálise têm sido propostos^131,132,133^. A partir da análise de pacientes em HD, avaliando o risco de eventos CV e de morte em 5 anos, foi desenvolvido escore preditivo com inclusão de sete características (idade, sexo, DM, doença CV prévia, tipo de acesso vascular ao iniciar em diálise, relação monócito/linfócito e ácido úrico), resultando em melhor desempenho que o escore de Framingham^10^. Avaliando pacientes japoneses em HD (J-DOPPS) ao longo de um ano, desenvolveu-se um modelo incluindo seis variáveis (idade, DM, doença CV prévia, tempo da sessão de diálise, fósforo e albumina), também com melhor discriminação de risco que o escore de Framingham^132^. Um estudo europeu com pacientes em HD, seguidos por até 2 anos e incluindo dados clínicos e laboratoriais (idade, IMC, tabagismo, doença CV prévia, etiologia da DRC, PA pré-diálise, UF, hemoglobina, PCR, albumina, creatinina e cálcio), demonstrou-se boa predição de risco de mortalidade geral^133^. No conjunto, esses estudos mostram que o acréscimo de fatores de risco não tradicionais aos modelos preditivos melhorou sua acurácia em pacientes dialíticos, ainda que a taxa observada de morte persista superior à predita, fazendo crer que fatores de risco adicionais possam ser incluídos na construção de modelo mais definitivo^131,132,133^. De todo modo, não há ferramenta disponível para predizer o risco CV em pacientes dialíticos com ampla aceitação, e que contemple simplicidade e boa acurácia.

Principais mensagens:

- Pacientes com DRC devem ser considerados de alto risco CV (Classe I/Nível A).
- A avaliação do risco CV de pacientes com DRC pode ser realizada por modelos de cálculo de risco da população geral (Classe IIA/Nível B).
- A discriminação do risco CV em pacientes com DRC tem sua acurácia aumentada pelo emprego de modelos específicos que incluem fatores de risco tradicionais e não tradicionais relacionados à DRC e à diálise (Classe IIA/Nível B).

### Peptídeos Natriuréticos em Pacientes com Doença Renal Crônica

As dosagens séricas de peptídeos natriuréticos são usualmente utilizadas na prática clínica para o diagnóstico e seguimento de pacientes com IC^134^. O diagnóstico de IC é corroborado por pelo menos um dos seguintes fatores: elevações das concentrações séricas de BNP ou de NT-proBNP ou evidências objetivas de congestão pulmonar ou sistêmica de origem cardiogênica^134^.

Esses peptídeos, principalmente o NT-proBNP, parecem predizer o risco de desfechos CV e morte em indivíduos com alto risco CV^135,136,137,138,139,140,141^, incluindo o potencial de uso do NT-proBNP como variável isolada que se assemelha ou mesmo supera as predições de modelos de risco multivariados^136,137,138,139,140,142^.

Na Quadro 5 estão exemplificados alguns estudos mostrando a associação do NT-proBNP com risco CV aumentado na população com DRC^139,141,143,144,145,146,147^.

**Quadro 5:** Estudos em pacientes com doença renal, avaliando os peptídeos natriuréticos como biomarcadores de riscos de doenças, eventos e morte cardiovascular

Estudo	Desenho do estudo	Doença renal/ outras comorbidades	Resultados
TREAT^144^	Avaliação da inserção de NT-proBNP e troponina T a um modelo de predição multivariável	DRC, com DM2 e anemia	Aumentou em ^17,8^% a habilidade preditiva de desfechos CV
ALTITUDE^137^	Análise *post hoc* da predição de NT-proBNP (variável única) comparada a modelo com 20 variáveis	DRC e/ou doença CV	Preditor de risco de eventos e morte CV, similar ao modelo multivariável
CREATE^145^	NT-proBNP (variável única)	DRC (TFG 15–35 mL/min) e anemia	Nível plasmático elevado como preditor de risco de eventos CV e de progressão da DRC
Harrison TG et al., 2020^141^	Meta-análise com 49 estudos, avaliou BNP e NT-proBNP como marcador de risco de eventos CV e morte	DRC 5D	Níveis elevados de NT-proBNP e de BNP foram preditores de mortalidade CV e morte por todas as causas
Satoh A et al., 2021^147^	NT-proBNP com dosagem antes da primeira HD da semana	Pacientes em HD	Níveis séricos elevados foram preditores de risco de morte CV e geral

Abreviatura – DM2: diabetes *mellitus* tipo 2, CV: cardiovascular, DRC: doença renal crônica, TFG: Taxa de filtração glomerular pelo *clearance* de creatinina, HD: hemodiálise.

Em pacientes dialíticos, o NT-proBNP parece predizer independentemente de morte CV e por todas as causas^141,147^, além de ser um fator preditivo para sobrecarga de volume em pacientes em HD (com ou sem declínio da fração de ejeção do VE)^147,148,149^ e para o aumento do risco de hospitalizações por AVC^150^.

Como a disfunção renal faz-se acompanhar de concentrações aumentadas de BNP e NT-proBNP^141,151^, a estratégia de medições sequenciais é superior à de medida única em pacientes com DRC^141^. Assim, na indefinição de valores de corte de BNP e NT-proBNP para pacientes em diálise, valores sequenciais podem traduzir melhor e de forma dinâmica a trajetória da função cardíaca e o *status* de hidratação.

Principais mensagens:

- Recomenda-se realizar dosagens seriadas de peptídeos natriuréticos em pacientes dialíticos (preferencialmente o NT proBNP), se disponível, pois podem auxiliar na avaliação de sobrecarga volumétrica, identificação e controle de IC associada à DRC dialítica e, principalmente, na predição do risco de desfechos CV e morte (Classe I/Nível B).

### Rigidez Arterial (RA) Avaliada Pela Medida da Velocidade da Onda de Pulso (VOP)

O grau de RA é um marcador de saúde e de envelhecimento vascular. Uma das formas de estimá-lo é pela medida da VOP arterial, definida pela medida do tempo gasto (m/s) para que a onda de pulso (gerada pela sístole) transite entre dois pontos no sistema arterial, geralmente a artéria carótida e a artéria femoral. A VOP carotídeo-femoral (aórtica) reflete as propriedades viscoelásticas da aorta, sendo considerada o método padrão-ouro para avaliação da RA^152^.

A elevação da VOP aórtica indica maior RA, sendo associada a um risco aumentado de doença CV e mortalidade^152^. Os valores normais para a VOP aórtica variam de acordo com idade e sexo (Tabela 2)^153^.

**Tabela 2:** Valores normais de VOP para homens e mulheres de acordo com a faixa etária

Faixa etária	Velocidade de onda de pulso (VOP) (m/s)	
	Homens	Mulheres
20–29 anos	6,4	6,0
30–39 anos	6,9	6,4
40–49 anos	7,4	7,0
50–59 anos	8,0	7,7
> 60 anos	9,2	8,6

Em pacientes em HD, o maior valor de VOP aórtica prediz maior mortalidade^154^, e VOP > 10 m/s é um preditor independente de mortalidade CV e por todas as causas^155^. Da mesma forma, a evolução acelerada da VOP (medida no tempo 0 e a cada 6 meses) pode prever a mortalidade CV em pacientes em HD^156^.

Os pacientes em HD parecem ter níveis de VOP menos sensíveis ao controle da PA, traduzindo a superposição dos fatores tradicionais de risco CV aos fatores não tradicionais (relacionados à uremia e ao tratamento dialítico), que são determinantes da maior RA que caracteriza essa população. No conjunto, isso pode justificar o reconhecimento de que a menor variação da VOP observada quanto ao manejo da HA nesses indivíduos associou-se a maior risco de eventos CV^157^.

O aumento da rigidez arterial é relativamente comum em pacientes com DP. Maior VOP em pacientes com DP está associado ao aumento da idade, presença de DM e hiperidratação, e se mostrou preditor de resultado em pacientes com DP^158^.

Em conclusão, a medida da VOP, ferramenta considerada padrão-ouro de RA, auxilia no cálculo do risco CV, ainda que mais pesquisas sejam necessárias para definir padrões definitivos de normalidade de VOP e seu papel em desfechos na população dialítica.

Principais mensagens:

- Recomenda-se a avaliação da rigidez arterial (RA) por meio da velocidade de onda de pulso arterial (VOP) carotídeo femoral, se disponível e de forma seriada, pois potencialmente prediz eventos CV e mortalidade em HD e DP (Classe I/Nível B).

### Bioimpedância (BIA) Como Método Diagnóstico da Composição Corporal e do *Status* Volêmico

A bioimpedância (BIA) avalia a composição corporal através da passagem de corrente elétrica de diferentes frequências pelo corpo, sendo um método diagnóstico empregado no contexto de pacientes dialíticos para estimativa do nível de hidratação e do apoio no gerenciamento da PA^159,160,161^.

Em revisão sistemática com meta-análise reunindo pacientes crônicos em HD e DP, Tabinor et al. sugerem que a mortalidade é predita pelo índice de hiper-hidratação > 15% e pelo ângulo de fase reduzido em 1 grau, independentemente de comorbidades^162^. No maior dos estudos incluídos, Zoccali et al. demonstraram maior mortalidade em pacientes hiper-hidratados, independentemente da PA pré-diálise^163^. Outras revisões sistemáticas e meta-análises se dedicaram a mostrar o impacto do uso da BIA em pacientes em diálise, e estão resumidas no Quadro 6 ^161,164,165,166,167^.

**Quadro 6:** Resumo dos principais estudos demonstrando o impacto no uso de bioimpedância em pacientes em diálise

Estudos	Tipo de estudo	Melhor controle da hiper-hidratação	Melhor controle da PA	Outros desfechos	Observações
Scotland et al., 2018^161^	Revisão sistemática	**sim**	não	I: não M: não	BIA sem efeito na rigidez arterial e não foi custo efetivo
Covic et al., 2017164	Revisão sistemática e meta-análise	**sim**	**sim**	M: não	Sem alteração da composição corporal
Beaubien- Souligny et al., 2020^168^	Revisão sistemática e meta-análise de estudos randomizados	**sim**	**sim**	HVE: não ECV: não **I: sim** M: não	-
Yang K et al., 2023^166^	Meta-análise de estudos randomizados	**sim**	**sim**	**HVE: sim (HD)** **M: sim**	**Redução do** **NT Pro-BNP e VOP**
Horowitz et al., 2023167	Revisão sistemática e meta-análise de estudos randomizados	**sim**	**sim**	HVE: não ECV: não I: não **M: sim**	-

Abreviaturas – PA: pressão arterial; BIA: bioimpedância elétrica; HVE: hipertrofia ventricular esquerda; ECV: eventos cardiovasculares; I: internação; M: mortalidade; VOP: velocidade de onda de pulso.

Importante destacar a diversidade de parâmetros advindos do uso da BIA para avaliar o estado volêmico do paciente dialítico, não havendo um consenso para a escolha preferencial de um deles^169,170^. Por outro lado, a literatura é mais robusta em indicar o uso da BIA como auxílio no controle da hipervolemia do que como orientador primordial na condução da terapia anti-hipertensiva. O uso da BIA pode ter impacto no controle da PA, porém é crucial perceber que deve ser vista como uma entre várias alternativas disponíveis para a gestão da volemia e PA.

Principais mensagens:

- Recomenda-se utilizar a BIA como método complementar para avaliação e manejo da hiper-hidratação e da PA em pacientes em HD e em DP (Classe I/Nível A).

### Ultrassonografia *Point-of-Care* (POCUS) na Avaliação da Volemia do Paciente Com DRC: Avaliação do Coração, Pulmão e Veia Cava Inferior (VCI)

A volemia é um parâmetro fundamental em todo o espectro da DRC, sendo que sua avaliação clínica tem limitações, estimulando o desenvolvimento de instrumentos de estimativa volêmica mais objetivos, como a ultrassonografia *point-of-care* (POCUS)^171^. A POCUS permite uma abordagem multiorgânica, com avaliação de facetas diversas do *status* de volume do paciente.

A abordagem *pump-pipes-leaks* é uma proposta de avaliação prática e objetiva da volemia. A insonação do coração através da janela cardíaca paraesternal eixo longo permite avaliar a função sistólica do VE a partir da variação dos seus volumes sistólico e diastólico, espessamento da parede lateral e do septo interventricular na sístole e amplitude do deslocamento septal do folheto anterior da válvula mitral (*pump*)^171^. “*Pipes*” alude à avaliação do diâmetro da VCI (Figura 3A-B).

**Figura 3:** Ultrassonografia *point-of-care* para identificação do *status* volêmico. Legenda: A e B, ultrassonografia da veia cava inferior (VCI): A, VCI < 1,7 cm, cujo diâmetro diminui mais de 50% com a inspiração, compatível com quadro de euvolemia; B: VCI pletórica, com diâmetro de 2,7 cm e que diminui menos que 50% com a inspiração, sugestivo de hipervolemia; C: ultrassonografia pulmonar: linhas-B identificam síndrome intersticial pulmonar, como no edema agudo de pulmão; D: derrame pleural identificado pela imagem anecoica acima do diafragma; E: ascite, imagem anecoica no espaço hepatorrenal; e F: derrame pericárdico, identificado pela imagem anecoica no espaço pericárdico.

Um modelo mais objetivo de avaliação volêmica foi desenvolvido com exame Doppler das veias hepática, porta e interlobar renal, denominado *Venous Excess Ultrasound* (VExUS). A Figura 4 sumariza os padrões de Doppler das veias hepática, porta e interlobar renal no contexto de congestão venosa leve, moderada e grave. Destaca-se a baixa qualidade de evidências disponíveis para emprego de VExUS na avaliação volêmica do paciente com DRC^168^.

**Figura 4:** Padrões de Doppler das veias hepática, porta e interlobar renal no contexto de congestão venosa leve, moderada e grave.

Abreviaturas – VCI: veia cava inferior; a: contração atrial; S: sístole; D: diástole. Nota – baseado em Dinh V. POCUS 101 Vexus ultrasound score – fluid overload and venous congestion assessment.

*Leaks* denota a pesquisa de congestão pulmonar através da identificação das linhas-B (Figura 3C) com alta sensibilidade, mas menor especificidade para congestão, além de derrame pleural (Figura 3D), ascite (Figura 3E) e derrame pericárdico (Figura 3F), permitindo detectar congestão extravascular antes do início dos sintomas clínicos, e assim adequar o tratamento de pacientes dialíticos^172^.

No paciente em diálise, a identificação do PS é desafiadora. Se superestimado, o PS contribui para a sobrecarga persistente de volume e suas consequências; quando subestimado, o PS pode causar hipotensão intradialítica e complicações associadas. No contexto do paciente em tratamento dialítico, a POCUS pode fornecer informações valiosas.

A estratégia *pump-pipes-leaks* permite responder, à beira-leito, a questões relacionadas à interação entre ganho absoluto de volume, redistribuição multicompartimental do volume extracelular e o impacto adverso multiorgânico. Necessitamos de mais estudos para avaliar seu impacto na sobrevida do paciente em HD^172^.

Principais mensagens:

- Recomenda-se utilizar o instrumento de estimativa volêmica *pump-pipes-leaks* à beira do leito, incluindo o escore VExUS, que podem ser uma alternativa para avaliar a gravidade de congestão venosa em pacientes com DRC (Classe II A/Nível B).

## Metas Pressóricas na Diálise Peritoneal (DP) e Hemodiálise (HD)

As metas de PA em pacientes em diálise, independentemente da modalidade, permanecem controversas, e a extrapolação dos alvos pressóricos da população geral para esse subgrupo não parece a melhor opção. Encontra-se documentado na literatura, em estudos observacionais, que há uma curva U ou J no controle pressórico de pacientes em HD e em DP, mas podem existir vieses de interpretação, por terem incluído indivíduos que apresentavam hipotensão, doença CV grave ou fragilidade, contribuindo para o mau prognóstico^5,120^,^173,174^.

O CRIC Study (*The Chronic Renal Insufficiency Cohort Study*), um estudo de coorte prospectivo multicêntrico, apresentou resultados interessantes sobre a relação entre mortalidade por todas as causas e PAS. Entre os participantes que iniciaram a HD (n = 326), foi observada associação em forma de U entre PAS medida “na unidade de diálise”, presumidamente antes do início da sessão, e mortalidade (HR 1,26 a cada aumento de 10 mmHg), enquanto a PAS “fora da unidade de diálise” teve associação linear com a mortalidade. Esses resultados sugerem que a PA alvo ideal para o tratamento deve ser determinada pela monitorização ambulatorial da PA (MAPA), que nem sempre está disponível, é bem tolerada ou considerada custo-efetiva^100,175^.

Um estudo americano envolvendo mais de 17 mil pacientes em HD concluiu que pacientes com PAS pré-diálise < 140 mmHg apresentaram mortalidade significativamente mais elevada, especialmente nos primeiros 3 meses após o início da HD^176^. Achados semelhantes relacionados a desfechos menos desfavoráveis com controle pressórico no período interdialítico foram encontrados em um centro brasileiro, que incluiu 2672 pacientes em HD seguidos por 31 meses^177^.

O estudo mais bem desenhado, com potencial definidor de metas pressóricas, porém sob a forma de piloto, o *Blood-Pressure-in-Dialysis* (BID), randomizou 126 participantes para duas metas de PAS medida na clínica antes do início da sessão de diálise: intensiva (110–140 mmHg) ou padrão (155–165 mmHg), com o objetivo principal de avaliar a viabilidade e segurança comparativa no período de um ano de seguimento. O estudo mostrou a viabilidade da intervenção; mas, apesar do protocolo exigir que os investigadores ajustassem o peso pós-diálise como etapa inicial para atingir a PAS alvo, a meta de PAS intensiva só foi alcançada pelo uso de medicamentos anti-hipertensivos adicionais. Os autores indicaram que possivelmente houve manejo inadequado do volume extracelular para obtenção do peso seco^178^.

A diretriz do *Kidney Disease Outcomes Quality Initiative* (2005), não atualizada, recomenda arbitrariamente uma PA pré-diálise < 140/80 mmHg e pós-diálise < 130/80 mmHg em pacientes em HD, baseada principalmente na opinião de especialistas (Classe IIA/Nível C)^179^.

Como a PA é um biomarcador da “síndrome cardiovascular”, o manejo da HA deve abordar a prevenção e o tratamento de todas as suas complicações CV. Na ausência de dados provenientes de estudos clínicos controlados e randomizados em pacientes em diálise, os objetivos do tratamento devem ser extrapolados a partir dos estudos observacionais disponíveis e aqueles desenvolvidos na população em geral. Com base nessa premissa, é razoável apontar para uma PA pré-diálise ≤ 140/90 mmHg ou uma PA domiciliar ≤ 130/80 mmHg (Classe IIA/Nível C). Um grupo de especialistas participantes de uma iniciativa do KDIGO que discutiu controvérsias no manejo volêmico e da HA em diálise optou por não recomendar metas de PA para pacientes em TRS e definiu que estratégias gerais no manejo da HA se aplicam a pacientes em diálise, tais como “Individualizar alvos e agentes de PA de acordo com a idade, doença CV coexistente e outras comorbidades, além da tolerância ao tratamento” e “Indagar sobre tontura postural e verificar hipotensão ortostática”^180^.

Nos pacientes em DP, um estudo observacional mostrou que PAS ≤ 110 mmHg foi associada a aumento da mortalidade, e um efeito protetor foi observado com uma PAS > 120 mmHg^181^. Assim, com base em dados da população geral e da população com DRC, recomenda-se que a PA alvo seja < 140/90 mmHg em pacientes com DP, em concordância com a *International Society of Peritoneal Dialysis* (ISPD) e outros autores (classe IIA/Nível B)^120,182,183^.

Na ausência de evidências de ensaios clínicos randomizados e controlados, de longo prazo, desenhados com a finalidade específica de definir os alvos ideais de PA em HD e DP, recomenda-se o desenvolvimento de pesquisas clínicas comparando diferentes limiares de medida da PA em domicílio em relação a desfechos clínicos e mortalidade^184,185,186,187,188^.

No Quadro 7, podemos observar as recomendações dos principais *Guidelines* que citam metas a serem atingidas em pacientes em HD.

**Quadro 7:** Metas pressóricas na hipertensão arterial de pacientes em diálise, segundo os principais *Guidelines*

*Guidelines*	Meta de pressão arterial (mmHg)
KDOQI 2005^179^	Pré-diálise: < 140/90 Pós-diálise: < 130/80
KDOQI 2015 (*update*)^186^	Não cita e alega falta de dados
EURECA-m (ERA – EDTA) 2017175	Não cita e alega falta de dados
*Japanese Society for Dialysis Therapy* 2012^187^	Pré-diálise: < 140/90
Diretrizes Brasileiras de HA 2020^4^	Pré-diálise: ≤ 140/90 Pós-diálise: ≤ 130/80
2020 *International Society of Hypertension Global Hypertension Practice Guidelines* ^188^	Não cita
*International Society of Peritoneal Dialysis* (ISPD) 2015^120^	Diálise peritoneal: < 140/90 mmHg

Principais mensagens:

- Recomenda-se que as metas pressóricas devem ser pré-HD: ≤ 140/90 mmHg e pós-HD ≤ 130/80 mmHg (Classe IIA/Nível B).
- Recomenda-se que a meta de PA em DP seja < 140/90 mmHg (Classe IIA/Nível B).
- Recomenda-se a prescrição de metas individualizadas para pacientes em HD e DP, de acordo com idade, grau de fragilidade, tolerância, presença de doença CV grave e comorbidades (Classe IIA/Nível B).
- Recomenda-se a realização de ensaios clínicos randomizados, controlados e comparativos de metas pressóricas para uma indicação baseada em evidências de alta qualidade.

## Tratamento não Medicamentoso da ha na DP e HD

### A Importância de Conhecer, Atingir e Manter o Peso Seco

O PS representa aquele no qual não se verificam sinais ou sintomas de hipervolemia ou hipo-hidratação. Para cada sessão de HD, é necessário definir um peso-alvo (eventualmente superior ao PS), levando-se em consideração o *status* volêmico, o GPID e a taxa de UF tolerada, de forma a se evitar o peso-alvo demasiadamente baixo (porque pode levar à hipotensão e acelerar a perda da FRR)^9,105^, ou inapropriadamente elevado incorrendo em HA e hipervolemia^9^. A hipervolemia crônica pode causar HA e aumento do risco de internação^189^ e de morte^163,190^ (Figura 5). Prioriza-se então a meta de atingir o PS ao longo das sessões de HD, evitando assim a hipervolemia crônica, mesmo com maior risco de hipotensão intradialítica^105^.

**Figura 5:** Consequências da manutenção da hipervolemia.

Abreviatura – UF: ultrafiltração.

A aplicação regular de um protocolo de avaliação do PS, preferencialmente com múltiplos instrumentos^105^, foi associada à menor mortalidade, e o uso da medida da PA ortostática foi associado a menor risco de hospitalização e eventos CV^191^. Ainda assim, a confiabilidade dos sinais clínicos na estimativa acurada da volemia tem sido questionada^192^.

Em DP, para atingir o PS e o controle da PA, prioriza-se a otimização do débito urinário com uso de diuréticos (em pacientes com FRR)^193^ e da UF peritoneal, com o ajuste apropriado da carga de glicose considerando a prescrição de DP e a velocidade de transporte peritoneal^5,9^. Recomenda-se para esses pacientes a adoção da preservação da FRR, do manejo dietético e de esforços para limitar a lesão peritoneal.

As barreiras para se atingir o PS são apresentadas na Figura 6.

**Figura 6:** Barreiras usuais de não atingimento do peso seco em pacientes em hemodiálise.

Abreviaturas – UF: ultrafiltração; HA: hipertensão arterial.

Principais mensagens:

- Para controle da HA, recomenda-se a estratégia de ajustar gradualmente a UF até sintomas mínimos de hipovolemia, a fim de definir e atingir o PS (Classe I/Nível B).
- Recomenda-se que a avaliação do PS seja feita regularmente e com múltiplos instrumentos, tais como avaliação clínica, ultrassonografia e bioimpedância (Classe I/Nível B).
- Em DP, recomenda-se estratégias para a preservação da FRR e da integridade da membrana peritoneal (Classe I/Nível B).

### Mudanças nos Parâmetros Dialíticos na DP e HD

Há fatores relacionados aos parâmetros dialíticos que podem interferir na PA, tanto em HD quanto em DP (Figura 7).

**Figura 7:** Fatores relacionados à modalidade de diálise e ao paciente que podem interferir no controle da pressão arterial.

Abreviaturas – HD: hemodiálise; HDF: hemodiafiltração on-line; UF: ultrafiltração.

Os estudos que analisaram a concentração de sódio na solução de diálise de HD não são conclusivos quanto ao seu impacto na PA e já foram detalhados no Capítulo 2, item 2.2.2 (Parâmetros que interferem na PA)^27,29^. Não há evidências que comprovem a redução da mortalidade na individualização do sódio^27^.

O benefício do perfil de sódio empregado na expectativa de evitar hipotensão intradialítica e diminuir o aporte desse elemento é controverso^103,191,194^.

Em um estudo, o uso desse perfil associou-se a balanço positivo de sódio, determinando maiores taxas de GPID, elevação da PA, eventos CV e morte^191^. Outro estudo mostrou que o perfil linear não reduziu hipotensão, embora o perfil “em etapas” (*stepwise*) tenha se mostrado eficaz^194^.

Os métodos de ajuste de UF incluem o emprego de perfil, UF isolada seguida de HD (diálise sequencial) ou dispositivos de *biofeedback* ^9,195^. Um estudo avaliando perfil linear de UF não mostrou menor risco de hipotensão, nem diferenças nos níveis de troponina e função do VE^196^. Os dispositivos de *biofeedback* de UF podem diminuir hipotensão intradialítica^195^.

A redução da temperatura da solução de diálise (em diferentes definições: diminuição em relação à temperatura corporal, fixa em 35,5ºC ou 36ºC, ou ajustada em sistema de *biofeedback*) associa-se à diminuição da hipotensão intradialítica^197^ e da mortalidade CV^191^, além de ser bem tolerada^197^. Estudo em andamento (MY TEMP, NCT02628366) avaliará o resfriamento da solução de diálise em relação a desfechos de internação e óbito por eventos CV.

Em DP, as manobras sugeridas para controle da HA incluem^9^: otimização do débito urinário com uso de diuréticos (em pacientes com FRR)^193^; reduzir o tempo de permanência intraperitoneal (de soluções à base de glicose) em pacientes alto transportadores; soluções à base de glicose de maior tonicidade (menos preferível, pelo impacto peritoneal e sistêmico); solução de icodextrina (aumento da UF sem maior carga de glicose)^198^, dialisato de baixo sódio, pois reduz HA, sem alteração de adequação)^193^ e soluções de maior biocompatibilidade, pH neutro ou menor conteúdo de produtos finais de degradação da glicose para preservação do peritônio e da FRR^198–200^.

Principais mensagens:

- Recomenda-se cautela no ajuste da concentração de sódio na solução de diálise com finalidade de controle pressórico e volêmico (Classe I/Nível B).
- Recomenda-se cautela no uso do perfil de sódio para evitar hipotensão intradialítica devido ao risco de balanço positivo e seus malefícios (Classe I/Nível B).
- Não se recomenda que o uso de perfil linear de UF seja utilizado para prevenir episódios de hipotensão intradialítica (Classe I/Nível B).
- Recomenda-se o resfriamento da solução de diálise para prevenir hipotensão intradialítica (Classe I/Nível A).
- Para controle da HA em DP, recomenda-se a preservação da FRR e da membrana peritoneal (Classe I/ Nível B).

### Mudanças na Modalidade de Hemodiálise (HDF, HD Diária)

As alternativas à HD convencional incluem regimes intensivos (com aumento da frequência e/ou da duração, como na HD curta diária e na HD longa diurna ou noturna), e o emprego de convecção (HDF), podendo ser realizados em centros de HD ou em domicílio. HD diária é compreendida na literatura como qualquer esquema de 5 a 7 vezes por semana^201,202,203,204,205^.

O emprego de HD diária permite reduzir: a PA^201,202,203^, a quantidade de medicamentos anti-hipertensivos^203^, a HVE^202^, os fatores inibidores teciduais da metaloproteinase^202^ e o fósforo, que, por sua vez, se associa à redução do FGF23^202^. Estudo multicêntrico de HD curta diária em ambiente domiciliar confirmou redução do número de anti-hipertensivos^204^. A HD intensiva também pode resultar na redução de hospitalizações (em relação à HD convencional) e da mortalidade (em comparação com HD convencional ou DP)^205^.

Alguns estudos mostraram que HD noturna reduziu a PAS^202,206,207^, o número de anti-hipertensivos^207^ e a HVE^202,207^, além de proporcionar melhora da anemia^206^ e da hiperfosfatemia^202,206,207^.

A hemodiafiltração on-line de alto volume convectivo (HDF) proporciona menor risco de morte por todas as causas, em contraste com a HD de alto fluxo^208^. Estudo avaliando parâmetros peridialíticos da PA não mostrou benefício da HDF em relação a HD convencional^209^. Em outro estudo, a HDF reduziu a mortalidade CV e episódios de hipotensão^210^. Para reduzir a hipotensão intradialítica, o emprego intermitente de infusões de dialisato puro trouxe benefícios para idosos e aqueles com alto GPID^211^.

Principais mensagens:

- Recomenda-se, sempre que disponível, utilizar regimes intensivos de HD (diário e/ou longo) com benefícios na redução da PA (Classe I/Nível A), da medicação anti-hipertensiva (Classe I/Nível A) e da massa ventricular esquerda (Classe I, Nível B).

### Recomendações Dietéticas e de Ingestão Hídrica Para Pessoas com HA em DP e HD, Especialmente Para Prevenir o Ganho de Peso Interdialítico (GPID)

Recomenda-se que a ingestão de sódio na dieta para a população hipertensa (não dialítica) seja de até 2 g/dia, correspondente a 5,0 g/dia de sal^4^. Em indivíduos em diálise, tipicamente sensíveis ao sal, há maior impacto da restrição de sal sobre a PA, sugerindo-se uma ingestão dietética de até 1,5 g de sódio ou 3,6 g de sal^5^. Em comparação com o tratamento anti-hipertensivo, a restrição dietética de sódio associada a uma UF mais intensiva, resultou em diminuição do GPID, da HVE, da carga anti-hipertensiva e de episódios de hipotensão intradialítica^212^.

Uma meta-análise identificou que a redução de pelo menos 1 g de sal associa-se à redução da PA^213^. Por outro lado, a ingestão elevada de sódio relacionou-se a risco aumentado de hipervolemia e de morte, mas sem associação com a PA^214^.

Adicionalmente, a hiperglicemia pode aumentar a sede e a ingestão de sal, levando a um aumento do GPID e da PA^215^. Em pacientes em diálise, a dieta mediterrânea parece melhorar o remodelamento miocárdico^216^, mas não foi capaz de reduzir a mortalidade, assim como dietas DASH (*Dietary Approaches to Stop Hypertension*) e vegetariana^217^. Por sua vez, dietas ricas em polifenóis melhoraram a PA diastólica na população dialítica^218^.

Principais mensagens:

- Recomenda-se a restrição de sódio na dieta de 1,5 a 2,0 g/dia para paciente dialítico, para redução da PA e GPID (Classe I/Nível B).

### Exercícios Físicos Para Hipertensos em DP e HD

Estudos que verificaram o efeito do exercício físico sobre a PA em pacientes dialíticos produziram resultados divergentes. Uma meta-análise não identificou efeito do treinamento físico sobre a PA^219^, mas em outra meta-análise de 78 estudos randomizados com 3326 participantes, foi possível evidenciar maior redução da PAD com exercícios aeróbicos combinados com exercícios resistivos, que envolvem carga tolerada pelo paciente nos MMII e MS sem FAV.^220^ Por outro lado, são documentadas melhoras em variáveis associadas a desfechos CV, lesões em órgãos-alvo, qualidade de vida e função cognitiva^219,220^. Para os tipos de exercício físico, há evidências de benefícios tanto com exercícios aeróbicos quanto resistivos. Quanto ao local, exercícios intradialíticos ou domiciliares podem ser empregados^221^.

Principais mensagens:

- Recomenda-se o estímulo à atividade aeróbica com duração mínima de 30 minutos, pelo menos 5 vezes na semana, para pacientes tanto em DP quanto em HD (Classe IIA/ Nível B).
- Recomenda-se que exercícios resistivos supervisionados também devem ser prescritos (Classe IIA/Nível B).
- Recomenda-se que seja realizada atividade física, intradialítica ou no intervalo interdialítico, fora do ambiente de diálise (Classe IIA/Nível B).
- Recomenda-se que, sempre que possível, os exercícios físicos sejam supervisionados por educadores físicos e/ou fisioterapeutas nas unidades de diálise (Classe IIA/Nível C).

### Outras Mudanças no Estilo de Vida (Espiritualidade, Controle de Estresse etc.)

Espiritualidade e religiosidade são instrumentos potencialmente importantes para o paciente dialítico, relacionando-se positivamente com a interação médico-paciente, qualidade e esperança de vida, enfrentamento da doença, e do tratamento e suas consequências, sendo por isso objetos de consideração pelos profissionais^222^.

A espiritualidade pode ser entendida como a busca de significados e propósitos de vida, e como a transcendência de si, que é uma vivência que pode desenvolver-se por meio da religiosidade e/ou crença em Deus, família, naturalismo, racionalismo, humanismo e artes, por exemplo^222^. A religiosidade, por sua vez, implica a relação do ser humano com um ser transcendente^222^.

Esses conceitos correspondem a uma construção psicológica na relação com doenças crônicas, convertendo uma situação pessoalmente dramática em algo útil^223^. As fortes crenças religiosas em pessoas em diálise foram correlacionadas com percepções atenuadas do fardo da doença e maior sensação de apoio social^223,224^.

A adesão à diálise é afetada pela fé religiosa, idade e educação: (a) muçulmanos são aderentes ao desejo de viver, e menos frequentemente interrompem a diálise^215,223^; (b) cristãos com religiosidade extrínseca (comportamento religioso) e intrínseca (fortes crenças e compromissos) manifestam maior adesão^223^; (c) idade mais avançada e maior tempo de diálise correlacionaram-se com maior adesão^223,225^; (d) aconselhamento e educação do paciente fornecem melhores resultados, maior adesão e potencial redução nos custos relacionados à saúde^223^.

Em alguns centros de diálise, assistentes sociais e psicólogos têm apoiado e estimulado atividades religiosas não formais (literatura religiosa, oração, grupos de discussão) como métodos positivos de enfrentamento^223,226^.

Principais mensagens:

- Recomenda-se o respeito e o estímulo à religiosidade e à espiritualidade como medida de melhor adesão e enfrentamento da DRC dialítica, envolvendo a equipe multiprofissional (Classe I/Nível B).

## Tratamento Medicamentoso da ha na DP e HD

Existe aparente paradoxo em relação ao uso de medicamentos anti-hipertensivos e o controle pressórico nos pacientes em diálise, pois, quanto maior o número de medicamentos, maior a chance de a PA não estar controlada, sugerindo que o uso combinado dos diferentes anti-hipertensivos dificulta atingir a meta do peso seco^227^. Os anti-hipertensivos devem ser utilizados, se necessário, após controle volêmico, sendo preferível os medicamentos avaliados em ECR, embora estes sejam pouco disponíveis e com número limitado de participantes, dificultando a generalização de diretrizes para a utilização de anti-hipertensivos na DRC dialítica. O frequente comprometimento CV e a presença de comorbidades nos pacientes em diálise torna a combinação de fatores tão diversa e peculiar que algumas diretrizes recomendam uma abordagem individualizada em relação às classes de medicamentos a serem utilizados^3,9,48^.

O uso de anti-hipertensivos em pacientes em diálise traz benefícios, reduzindo a morbidade e a mortalidade geral e CV^228,229^. Todas as classes de anti-hipertensivos podem ser utilizadas no controle da PA em pacientes em diálise, desde que sejam considerados riscos e benefícios^5^.

### Diuréticos

O uso desses medicamentos não tem respaldo em pacientes em HD ou em DP, sem diurese residual (< 100 mL/dia). Nos pacientes em DP, o uso de diuréticos pode melhorar o *status* volêmico e minimizar a necessidade de soluções contendo maior concentração de glicose, embora os *Guidelines* da *International Society of Peritoneal Dialysis* não recomendem seu uso^120^. Em pacientes em HD, os diuréticos podem auxiliar na redução do GPID, resultando em diminuição das taxas de UF e menos episódios de hipotensão intradialítica.^230,231^

Em um estudo coorte prospectivo com 5219 pacientes que continuaram utilizando diurético de alça após o início de HD comparado a 6078 controles que não o fizeram, verificou-se menores taxas de hospitalização, hipotensão intradialítica e GPID, sem diferença significante na PA ou na taxa de mortalidade no primeiro ano de diálise. Nos EUA, o diurético mais utilizado em pacientes em HD é a furosemida, em doses de 20 a ≥ 320 mg/dia^232^.

Um estudo prospectivo observacional acompanhou 16420 pacientes em HD em três continentes e observou que o uso de diuréticos (> 90% diuréticos de alça) nos primeiros 3 meses de HD foi maior no Japão (47,8%) e na Europa (45,1%) em comparação aos Estados Unidos (2,4%), caindo progressivamente ao longo desse período^233^. Nesse estudo, usar diuréticos associou-se a menor GPID e menor chance de hipercalemia. Pacientes com FRR em terapia diurética tiveram quase o dobro de chances de manter a diurese residual após um ano de acompanhamento comparado aos pacientes que não utilizavam diuréticos. Os pacientes que receberam diuréticos tiveram menor risco de mortalidade geral (–7%; p = 0,12) e CV (–14%; p = 0,03)^233^.

Não foram encontrados estudos que avaliaram o uso de diuréticos tiazídicos ou tiazídicos-símile como monoterapia em pacientes em DP ou HD. Um estudo com tripla terapia diurética em doses altas (furosemida 1000 mg/dia, hidroclorotiazida 100 mg/dia e espironolactona 50 mg/dia) em 51 pacientes em DP mostrou aumento da diurese e melhor controle de volume em comparação com a furosemida isoladamente^234^.

Meta-análise que incluiu 28226 pacientes em HD evidenciou benefício potencial em reduzir hipotensão intradialítica, mortalidade CV e por todas as causas^231^.

Principais mensagens:

- Recomenda-se o uso de diuréticos de alça em pacientes em HD e DP, enquanto houver diurese residual (Classe I/Nível B).

### Inibidores do Sistema Renina Angiotensina-Aldosterona (SRAA)

Os inibidores do SRAA, IECA e BRA são os mais utilizados em DRC em seus diferentes estágios. Meta-análise de ensaios clínicos randomizados que avaliou o efeito anti-hipertensivo das diferentes classes de medicamentos em comparação à placebo ou entre si observou que os IECA/BRA reduzem a PA de forma modesta quando comparados a placebo (–4,3 mmHg na PAS), como esperado para o estado de hipervolemia próprio da DRC em fase dialítica^235^. Quando comparados à placebo, os antagonistas dos receptores mineralocorticoides (ARM) são os mais potentes em reduzir a PA (–10,8 mmHg), seguidos pelos BB (–8,7 mmHg) e BCC (–4,6 mmHg)^235^.

Em pacientes em diálise, o uso de IECA ou BRA está associado a redução do índice de massa ventricular esquerda, de eventos mórbidos, particularmente IC, e de mortalidade CV^236,237,238^. Além disso, há evidências de que a proteção renal dessas classes de medicamentos continua ocorrendo nessa fase da doença, pois seu uso está associado à manutenção da função renal ou diurese residual, o que permite melhor controle volêmico e reduz o risco de hipotensão intradialítica^239,240^. Em pacientes em DP, o uso de IECA/BRA preferencialmente, associa-se à preservação da membrana peritoneal com redução da fibrose e manutenção da depuração peritoneal e da UF^241,242^. O risco de hiperpotassemia é plausível, mas controverso, pois não é observado em todos os estudos^243,244.245^.

Principais mensagens:

- Recomenda-se o uso de IECA ou BRA em pacientes em HD e DP como medicamentos anti-hipertensivos por seus efeitos pleiotrópicos: redução de HVE, IC, redução de fibrose peritoneal em DP e mortalidade CV (Classe I/Nível B).
- Recomenda-se o acompanhamento dos níveis séricos de potássio, embora o risco de hiperpotassemia seja controverso e nem sempre observado (Classe IIA/Nível B).

### Betabloqueadores (BB)

Na DRC dialítica os BB são recomendados como medicação preferencial. Dois ECR evidenciaram a eficácia e a proteção CV proporcionada pelos BB na subpopulação de pessoas em diálise, sendo o primeiro, um ECR contra placebo, que demonstrou que o carvedilol utilizado em 114 pacientes em HD e IC (miocardiopatia dilatada e fração de ejeção reduzida), resultou em melhora clínica, redução (–49%) da mortalidade geral e CV (–68%) e das internações por IC (–81%)^58^. O segundo estudo propôs comparar o efeito de redução da HVE com atenolol ou lisinopril (administrados após as sessões de HD) em pacientes em HD com PA ≥ 140/90 mmHg e HVE, mas foi interrompido precocemente porque os resultados intermediários foram tão benéficos a favor do atenolol que não seria ético continuar o estudo^246^. Embora com número limitado de participantes, 100 em cada grupo, o estudo tornou-se uma referência. As taxas de eventos CV graves e de internações foram pelo menos metade no grupo randomizado para o atenolol quando comparado ao lisinopril^246^. A MAPA de 44h foi consistentemente menor no grupo que recebeu atenolol, ainda que os participantes do grupo com lisinopril tivessem recebido mais medicamentos anti-hipertensivos e reduzido o peso seco em 3 quilos a mais durante o acompanhamento, indicando a superioridade do atenolol sobre lisinopril^246^. Outros estudos com BB (carvedilol, bisoprolol e atenolol) mostraram resultados semelhantes em relação à redução da PA, em média –8,7 mmHg na PAS^235^.

Há controvérsia na literatura em relação aos BB dialisáveis (atenolol, bisoprolol, metoprolol, nadolol) e os não dialisáveis (carvedilol, nebivolol, propranolol, pindolol)^247^. Alguns estudos apresentam dados objetivos e sugerem que os BB dialisáveis são superiores aos não dialisáveis, pois, quando comparados, apresentam redução dos eventos CV maiores (AVC, IAM e ICC) e da mortalidade CV e geral^248,249^. Existe também uma meta-análise de estudos observacionais que não encontrou diferenças entre os efeitos protetores CV dos BB dialisáveis e não dialisáveis em pessoas em HD^250^. Todos os estudos que avaliaram possíveis diferenças na proteção CV entre BB dialisáveis e não dialisáveis são observacionais, coortes retrospectivas ou prospectivas e baseados em dados de prescrição de medicamentos.

Independentemente do BB, há uma pergunta importante que se impõe: sua associação com outros anti-hipertensivos, poderia ter efeito benéfico somatório? Há pelo menos um estudo observacional que avaliou esse aspecto em pacientes em HD e que desenvolveram IC após início da diálise e passaram a utilizar BB, IECA/BRA ou ambos^251^. No acompanhamento da mortalidade por 5 anos, os pacientes que utilizaram apenas BB foram considerados referência para os que utilizaram apenas IECA/BRA, para os que utilizaram BB e IECA/BRA e para os que utilizaram quaisquer outros medicamentos. Em relação à referência (uso apenas de BB), a mortalidade para os que utilizaram apenas IECA/BRA foi semelhante (+8%; não significância estatística), para os que utilizaram a associação de BB e IECA/BRA a mortalidade foi menor (–33%; p < 0,001) e para os que não utilizaram BB nem IECA/BRA a mortalidade foi maior (+74%; p < 0,001)^251^.

Principais mensagens:

- Recomenda-se a utilização de betabloqueadores como medicamentos preferenciais nos pacientes em HD e DP, exceto se contraindicados (Classe I/Nível B).
- Recomenda-se o uso preferencial de atenolol, exceto se contraindicado (Classe I/Nível B).
- Não há evidências suficientes sobre o uso preferencial daqueles dialisáveis *vs*. não dialisáveis (Classe IIB/Nível B).

### Bloqueadores dos Canais de Cálcio (BCC)

Existem poucos estudos avaliando os BCC no tratamento da HA e na prevenção de complicações CV em pacientes em diálise. Uma meta-análise de estudos randomizados^235^ encontrou apenas 3 estudos com BCC comparados a placebo (nitrendipino, diltiazem e anlodipino), nos quais, em média, o efeito hipotensor foi semelhante aos IECA (-4,6 mmHg na PA sistólica). O ECR mais robusto incluiu 251 pacientes e comparou anlodipino *vs.* placebo, e evidenciou redução média de -10 mmHg na PAS, redução não significante da mortalidade geral, desfecho primário, (RR = 0,62) e redução significante (RR = 0,53; p = 0,03) nos desfechos secundários (IAM, AVC, revascularização miocárdica e doença arterial periférica com necessidade de revascularização ou amputação)^252^. Observou-se que o efeito hipotensor da nitrendipina foi mais pronunciado nos pacientes com maior GPID^253^.

Uma revisão sistemática com meta-análise identificou 13 estudos randomizados ou quasi-randomizados envolvendo o uso de BBC em pacientes em diálise^254^. Embora o número de pacientes incluídos nessa análise fosse grande (1459), a diversidade dos estudos e o baixo grau de confiança limitaram as conclusões^254^.

Principais mensagens:

- Recomenda-se o uso de BCC em pacientes em HD e DP, pois mantém seus efeitos anti-hipertensivos e protetores CV mesmo na presença de hipervolemia (Classe I/Nível B).

### Antagonistas dos Receptores Mineralocorticoides (ARM)

Uma meta-análise incluiu 1133 pacientes em HD em 11 estudos com ARM (10 com espironolactona e 1 com eplerenone) e observou maior efeito hipotensor quando comparado a placebo (–10,8 mmHg na PAS) e maiores taxas de descontinuação do medicamento, embora sem risco adicional de hiperpotassemia^235^.

Outra meta-análise, que incluiu 1309 pacientes em HD, em 14 ECR que envolveram o uso de ARMC (13 com espironolactona) *vs.* placebo ou sem tratamento, evidenciou importante redução de eventos CV não fatais (–49%), redução de mortalidade geral (–56%) e CV (–59%), sem causar hiperpotassemia estatisticamente significante^255^.

Uma meta-análise que incluiu 829 pacientes em diálise oriundos de 9 ECR com ARM observou redução significante da mortalidade geral (–60%) e CV (–66%), mas foi observado aumento em 3 vezes do risco de hiperpotassemia e até 5 vezes de ginecomastia^256^. Nas meta-análises disponíveis, ainda que a hiperpotassemia possa ser um risco, parece não mitigar o efeito benéfico sobre a mortalidade CV e geral^255,256^.

Dois grandes ECR em andamento poderão fornecer dados mais confiáveis sobre a eficácia e segurança do uso de espironolactona na DRC dialítica. O *Aldosterone Antagonist Chronic Hemodialysis Interventional Survival Trial* (ALCHEMIST), que já recrutou 825 pacientes em HD e deve ser concluído em 2024, avalia os efeitos da espironolactona em relação aos desfechos clínicos de interesse. O estudo *Aldosterone Blockade for Health Improvement Evaluation in ESKD Trial* (ACHIEVE) planeja inscrever 2750 pacientes em diálise e comparar os efeitos da espironolactona em relação à morte CV ou hospitalização.

Ressalte-se que os estudos existentes são predominantemente com espironolactona, com maior risco de ginecomastia e risco de hiperpotassemia baixo e controverso (Classe I/Nível B). Não há ECR, até o presente momento, que tenha utilizado ARM não esteroidais seletivos em HD ou DP.

Principais mensagens:

- Recomenda-se o uso dos ARM em pacientes em HD, pois têm bom efeito anti-hipertensivo e reduzem a morbidade e mortalidade CV e geral (Classe I/Nível A). Para pacientes em DP, o número de estudos e as evidências são frágeis (Classe IIB/Nível B).

### Outras Classes de Anti-Hipertensivos

#### Simpatolíticos de ação central

Não há estudos com metildopa e foi encontrada uma revisão sistemática com meta-análise que concluiu que não há evidências que suportem o uso de clonidina cronicamente em HD, além de seu uso estar associado a um perfil de efeitos colaterais significativos^257^.

#### Vasodilatadores de ação direta

Não há estudos com vasodilatadores de ação direta, como a hidralazina e o minoxidil na população em diálise. Hidralazina tem sido utilizada em associação à isossorbida em pacientes em HD com IC com fração de ejeção reduzida, mas não como anti-hipertensivo^258,259^.

Entretanto, simpatolíticos e vasodilatadores diretos são utilizados de forma empírica na DRC dialítica, particularmente na HA resistente e refratária. Um estudo realizado em 210 clínicas de diálise nos Estados Unidos identificou que, nos 6 primeiros meses de diálise, os simpatolíticos são utilizados por 19% dos pacientes e os vasodilatadores, por 4% a 10%^260^.

Principais mensagens:

- Recomenda-se que simpatolíticos de ação central e vasodilatadores diretos sejam utilizados como 5ª ou 6ª medicação anti-hipertensiva em pacientes em diálise, ou se houver contraindicações para os demais anti-hipertensivos (Classe IIA/Nível C).

### Depuração Renal e Pela Hemodiálise dos Anti-Hipertensivos

A necessidade de ajuste de dose ou reposição pós-diálise do anti-hipertensivo prescrito é uma preocupação recorrente do nefrologista no acompanhamento dos pacientes em HD. O Quadro 8 mostra um resumo dos anti-hipertensivos, disponíveis no Brasil, que têm eliminação renal ou que são mais extensamente dialisáveis e, por isso, podem requerer ajustes. Os anti-hipertensivos não mencionados não precisam de ajuste ou reposição pós-HD^261^.

**Quadro 8:** Principais anti-hipertensivos disponíveis no Brasil. Ajuste de dose na doença renal crônica e taxa de remoção pela diálise*

Classe e medicamento	Ajuste de dose na DRC	Remoção na diálise
**Inibidores ECA**		
**Captopril**	**TFG < 10 mL/min:** administrar 50% da dose a cada 24h.	**HD:** administrar uma dose extra após a HD. **DP:** remoção insignificante.
**Enalapril**	**TFG ≤ 30 mL/min:** 2,5 mg a cada 24h. Aumentar progressivamente de acordo com a PA.	**HD:** taxa de remoção 20%-50%. Dose extra de 2,5 mg após HD. **DP:** ajustar a dose para 25% da dose usual.
**Benazepril**	**TFG ≤ 30 mL/min:** 5 mg a cada 24h. Aumentar progressivamente de acordo com a PA.	**HD:** ajustar dose para 25% a 50% da dose habitual. Sem dose extra pós-HD. **DP:** ajustar dose para 25% a 50% da dose habitual.
**Lisinopril**	**TFG 10–30 mL/min:** 2,5 mg–5 mg a cada 24h. Aumentar progressivamente de acordo com a PA. **TFG < 10 mL/min:** considerar substituir por outro medicamento (risco elevado de EA).	**HD:** taxa de remoção de 50%. Recomendar o uso de 10 mg após HD (3x/semana), aumentando a dose progressivamente de acordo com a PA. **DP:** 2,5 mg a cada 24h. Aumentar progressivamente de acordo com a PA.
**Ramipril**	**TFG ≤ 40 mL/min:** 25% da dose habitual.	**HD:** remoção insignificante. **DP**: remoção insignificante.
**Perindopril**	**TFG < 30 mL/min:** uso não recomendado.	**HD:** usar dose após HD. **DP:** sem ajuste recomendado.
**Beta-bloqueadores**		
**Atenolol Bisoprolol Metoprolol Nadolol**	**TFG > 30 mL/min:** não necessita ajuste da dose. **TFG 10–30 mL/min:** metade da dose/dia. **TFG < 10 mL/min:** ¼ da dose/dia.	**HD:** taxa de remoção 25% a 50%. Uso da dose após HD. **DP:** remoção insignificante. Usar ¼ da dose/dia.
**Propranolol Pindolol** **Carvedilol Nebivolol**	Não necessita ajuste da dose.	**HD:** remoção insignificante. **DP:** remoção insignificante.
**Simpatolíticos**		
**Metildopa**	**TFG < 10 mL/min:** administrar a cada 12h a 24h.	**HD:** taxa de remoção até 60%. Uso da dose após HD. **DP:** administrar a cada 12h a 24h.
**Clonidina**	**TFG < 30 mL/min:** iniciar baixa dose e aumentar lentamente conforme PA e EA.	**HD:** remoção insignificante. **DP:** remoção insignificante.
**BRA**		
**Bloqueadores dos Receptores da Angiotensina**	Não necessita ajuste da dose.	**HD:** remoção insignificante. **DP:** remoção insignificante.
**BCC**		
**Bloqueadores dos canais de cálcio**	Não necessita ajuste da dose.	**HD:** remoção insignificante. **DP:** remoção insignificante.
**Diuréticos**		
**Diuréticos de alça**	Não necessita ajuste da dose.	**HD:** remoção insignificante. **DP:** remoção insignificante.
**Alfa-bloqueadores**		
**Alfa-bloqueadores**	Não necessita ajuste da dose.	**HD** remoção insignificante. **DP:** remoção insignificante.
**Vasodilatadores**		
**Vasodilatadores diretos**	Não necessita ajuste da dose.	**HD:** remoção insignificante. **DP:** remoção insignificante.

Abreviaturas – ECA: enzima conversora da angiotensina; TFG: taxa de filtração glomerular; DRC: doença renal crônica; HD: hemodiálise; DP: diálise peritoneal; EA: efeitos adversos; BRA: bloqueadores dos receptores da angiotensina; BCC: bloqueadores dos canais de cálcio. Notas –

*Quadro adaptado da referência^261^. Informações para ajuste de dose e remoção na diálise baseadas nas recomendações do *UpToDate* 2021 para cada medicamento citado.

### Horário da Administração ou Suspensão de Doses dos Medicamentos Pré-Diálise

A hipotensão intradialítica é sempre um risco para os pacientes em HD, particularmente se houver GPID excessivo ou uso de medicamentos que bloqueiam os mecanismos de defesa no caso de hipovolemia transitória durante a HD (simpatolíticos, BB e IECA/BRA). Por isso, é tentador reduzir a dose ou não administrar algum(ns) medicamento(s) no dia da HD ou horas antes dela. Há um único estudo randomizado por *cluster*, em cinco centros que mantinham a medicação anti-hipertensiva (65 participantes) e cinco centros que suspendiam a medicação no dia da HD (n = 66)^98^. Nos dois grupos, a medicação utilizada em dose única diária foi mantida como dose noturna. O estudo mostrou que não houve menor risco de hipotensão intradialítica e nem maior frequência de atingir o peso seco estimado no grupo em que a medicação foi suspensa no dia da HD, mas houve maior risco de hipertensão (PAS > 160 mmHg) pré-diálise^98^.

Principais mensagens:

- Não há evidências de benefícios em retirar ou diminuir a dose da medicação anti-hipertensiva no dia da HD (Classe III/Nível B).

### Terapia Combinada

As classes de anti-hipertensivos que apresentam perfil mais adequado, levando-se em conta o efeito sobre a PA, a redução de complicações CV e segurança, seriam: os BB, seguidos pelos BCC e IECA/BRA e, por fim, os ARM. No entanto, essa não é a realidade da prática clínica. A maioria dos pacientes chega ao tratamento dialítico usando BB, IECA/BRA e BCC^260^. Dessa forma, a estratégia inicial mais razoável deveria ser controlar a hipervolemia, alcançando o PS estimado, evitando a associação de anti-hipertensivos e (re)introduzindo-os de acordo com as características de cada paciente, na ordem sugerida acima. Os demais anti-hipertensivos poderiam ser associados, se necessário, apenas após atingido o estado de euvolemia.

## Situações Especiais de Hipertensão em Diálise

### Hipertensão em Gestante em DP e HD

Estima-se que a prevalência de gestações em mulheres em diálise seja de 1% a 7%, sendo mais frequente em pacientes em HD do que em DP^262,263^. A hipertensão crônica no início da gravidez é um fator importante para complicações maternas, mas não está associada a complicações neonatais (1/5 = 20,0% *vs*. 2/9 = 22,2% sem hipertensão crônica)^262,263^. Recentemente, tem sido observado um aumento no número de gestações em mulheres com DRC, possivelmente devido a melhorias no manejo da HA e redução de complicações como polidrâmnios^264^.

As complicações maternas mais comuns em mulheres com DRC, dialítica ou não, durante a gravidez são as doenças hipertensivas. Identificar a diferença entre pré-eclâmpsia e descompensação da DRC pode ser um desafio. A HA nessas pacientes pode estar relacionada à sobrecarga hídrica e o seu manejo com UF pode levar à hipoperfusão de órgãos-alvo, incluindo a placenta^262^. Não há estudos clínicos randomizados que estabeleçam a PA ideal para gestantes com DRC em diálise^265^. Os estudos disponíveis costumam seguir as recomendações de manejo da HA das principais diretrizes obstétricas^263,264,265,266^. Além disso, é necessário um controle rigoroso da PA, com uma meta de pressão < 140/90 mmHg ou uma PAD < 85 mmHg, de acordo com o estudo CHIPS^267^.

A gestação em pacientes em diálise apresenta melhores desfechos quando há uma intensificação do tempo e da dose de diálise, com base nos níveis séricos de ureia (abaixo de 50–70 mg/dL)^265^. Uma revisão sistemática destacou haver maior risco de complicações na gravidez em mulheres com DRC, incluindo pré-eclâmpsia (razão de chances [OR] 10,4, IC 95% 6,3–17,1), parto prematuro (OR 5,7, IC 95% 3,3–10,0), restrição de crescimento intrauterino ou baixo peso ao nascer (OR 4,9, IC 95% 3,0–7,8), cesarianas (OR 2,7, IC 95% 2,0–3,5) e falhas na gravidez [incluindo natimorto e morte fetal e neonatal (OR 1,8, IC 95% 1,0–3,1), em comparação com mulheres saudáveis do grupo de controle^264^.

A taxa de nascidos vivos em diálise tem apresentado melhorias significativas ao longo das décadas, evoluindo de 25%, em 1960, para mais de 75% atualmente. No entanto, 53,4% dos bebês ainda nascem prematuros e 65% com baixo peso ao nascer, ou seja, menos de 2,5 quilos^268^. Uma revisão que analisou 10 artigos revelou que a maioria das mulheres grávidas em HD segue regimes terapêuticos que variam de 15 a 40 horas semanais (em esquemas diários ou de quatro vezes na semana), meta de ureia abaixo de 60 mg/dL e de creatinina de 6 mg/dL. A mesma revisão mostrou que a maioria das pacientes utiliza um fluxo de dialisato de 500 mL/min, enquanto o fluxo de sangue e o tipo de dialisador variaram consideravelmente^269^. Outra revisão sistemática identificou uma taxa geral de nascidos vivos de 82%, com uma relação positiva entre o número de horas em diálise e melhores resultados, incluindo risco reduzido de parto prematuro antes das 37 semanas de gestação e recém-nascidos pequenos para a idade gestacional abaixo do percentil dez^270^.

Principais mensagens:

- O manejo da HA nas gestantes em DP ou HD deve ser feita de acordo com as diretrizes estabelecidas para o controle da PA na população geral.
- O aumento do tempo e da dose de diálise está relacionado a melhores desfechos maternos e fetais (Classe I/Nível 2A).

### Hipertensão Resistente e Refratária em Pacientes em Diálise Peritoneal e Hemodiálise

Segundo os principais *Guidelines*, a hipertensão resistente (HAR) é caracterizada pela falta de controle da PA (em geral PA < 140/90 mmHg), por mais de 3 meses, mesmo com o uso de três anti-hipertensivos, preferencialmente um diurético tiazídico, um inibidor do SRAA e um bloqueador de canal de cálcio ou o uso de quatro medicamentos com controle pressórico^271^. Já a HARf ocorre quando a PA permanece fora do alvo terapêutico, por mais de 6 meses, mesmo com o uso de cinco ou mais fármacos, incluindo um antagonista do receptor mineralocorticoide (espironolactona) e um diurético tiazídico de longa ação (clortalidona)^272,273^.

Para o diagnóstico dessas condições, é importante avaliar a adesão ao tratamento e confirmar o descontrole da PA por meio da MAPA ou da MRPA, conforme protocolos discutidos no Capítulo 3.

Apesar do mecanismo fisiopatológico da HAR estar diretamente relacionado à sobrecarga de volume, que se agrava na presença de DRC 5D, na prática clínica observa-se que muitos pacientes, ao iniciar o tratamento dialítico, ainda apresentam valores elevados de PA, apesar de adequada UF. Estudos relatam prevalência de HAR variando de 18%^274^ a 24%^6^.

Para melhor controle da HAR e HARf é importante garantir: adequado controle do volume, atingindo o PS ideal, além de prescrição individualizada de sódio no líquido dialítico, duração adequada da diálise e restrição da ingestão de sal no período interdialítico^274^.

O tratamento farmacológico para pacientes em tratamento dialítico com HAR ou HARf tem algumas particularidades. O uso de diuréticos não é recomendado para pacientes sem FRR e o tratamento medicamentoso segue as recomendações já descritas no Capítulo 7. Um estudo de intervenção não randomizado chamou a atenção para a resposta pressórica ao sacubitril/valsartan nesses pacientes, embora com amostra reduzida e duração curta^275^.

O uso de espironolactona tem se mostrado pouco eficaz para controle pressórico, mas tem efeito positivo na redução da mortalidade total e da incidência de eventos CV^276^.

Em casos mais desafiadores de controle da PA em pacientes HAR ou HARf, esgotadas as opções de outras classes anti-hipertensivas, podem ser considerados os vasodilatadores diretos, como a hidralazina ou o minoxidil, como opções terapêuticas^277^. Além disso, o uso da denervação renal em HAR em diálise tem sido proposto, embora ainda haja debates em torno dessa abordagem. Estudos recentes, como ECR, mostraram resposta favorável em curto e longo prazos da PA em pacientes submetidos a essa intervenção^278,279^. O tratamento medicamentoso segue as recomendações estabelecidas no Capítulo 7.

Principais mensagens:

- Recomenda-se que, para diagnóstico de HAR verdadeira e HARf, é necessário checar a adesão ao tratamento e confirmar, preferencialmente pela MAPA ou pela MRPA (Classe I/Nível A).
- Denervação renal pode ser uma opção terapêutica (Classe 2B/Nível C).

### Hipertensão Sistólica (HAS) no Idoso em Diálise

A hipertensão arterial sistólica isolada (HAS) é bastante prevalente nos pacientes idosos devido à perda da elasticidade dos grandes vasos torácicos. Por outro lado, sabe-se que a HAS, com ou sem elevação dos níveis diastólicos, avaliada no período interdialítico através da MAPA ou MRPA, é diagnosticada em mais de 70% dos pacientes DRC 5D^280^. Assim, os idosos, ao iniciarem o tratamento dialítico, como todas as outras subpopulações, podem apresentar aceleração do dano vascular, característico dos pacientes em DRC 5^281^.

O paciente idoso em diálise pode apresentar HAS, mesmo após ter atingido seu PS. Nessas situações, é necessário considerar a possibilidade de que os aumentos dos níveis sistólicos sejam consequência da rigidez vascular central.^274^ A chamada pseudo-hipertensão deve ser incluída no diagnóstico diferencial desses pacientes.^4^ Não é incomum prescrever a retirada gradual de fluidos por meio da UF, na tentativa de normalizar a PA nessas condições. No entanto, é importante ter em mente que o idoso com pseudo-hipertensão pode desenvolver hipovolemia intravascular com graves consequências CV. Além disso, é importante considerar que os idosos hipertensos em diálise também podem apresentar hipertensão secundária (ver item 8.5) e outros fenótipos de HA, como HAB e HM (ver item 3.4.3), e que as investigações clínicas devem ser feitas de forma menos invasiva possível.

A falta de ECR sobre os efeitos prejudiciais da HAS em pacientes dialíticos faz com que todas as recomendações sejam baseadas em opiniões dos especialistas. A situação se torna mais complexa quando se trata de sugestões para idosos submetidos à terapia dialítica.

### Hipertensão Arterial em Hemodiálise Associada ao Hiperfluxo da Fístula Arteriovenosa (FAV)

Os efeitos hemodinâmicos da instalação de uma fístula arteriovenosa (FAV)^282^ têm sido estudados em pacientes que precisam de HD. Antes de realizar a FAV, é muito importante cumprir uma avaliação abrangente da função cardíaca, com ênfase no VD, complementada por informações sobre o VE. Em conjunto, uma avaliação cardiovascular detalhada, eletrocardiograma e ecocardiograma transtorácico permitem avaliar as funções e estruturas cardíacas antes da FAV. Além disso, é essencial realizar uma avaliação minuciosa para estabelecer o diâmetro ideal da anastomose arteriovenosa, garantindo que a consequente redução do fluxo consiga diminuir a PA, com benefícios superiores ao risco de sobrecarga de volume e aumento de pressão no VD.

Alterações hemodinâmicas significativas ocorrem devido à redistribuição de fluidos do sistema arterial de alta pressão e baixa capacitância para um sistema de baixa pressão e alta capacitância, resultando na redução da resistência periférica, aumento da pré-carga do VD e da pós-carga VE^283^. Estudos clínicos mostram que a PAS e a PAD diminuem em curto e longo prazo, após a criação de uma FAV, mas aumentam após a sua ligadura. ^284,285,286^. Scholz et al.^283^ em meta-análise, confirmaram esses achados.

É muito importante avaliar os efeitos hemodinâmicos e cardíacos da FAV e suas possíveis complicações, como insuficiência cardíaca direita causada pelo aumento do retorno venoso, sobrecarga VD e remodelação VE. Além disso, efeitos colaterais como estenose venosa, aumento da pressão pulmonar e disfunção cardíaca direita podem ocorrer devido ao aumento do fluxo venoso^284,285 286^. Essas alterações podem levar à necessidade de fechamento da FAV em pacientes sintomáticos^287^. É fundamental considerar os efeitos em longo prazo dessa carga de volume artificialmente aumentada no coração direito. Reddy et al.^288^ mostraram que a criação de uma FAV resultou em dilatação significativa do coração direito e deterioração da função cardíaca direita, e consequentemente em insuficiência cardíaca em mais de 40% dos pacientes. Por outro lado, a criação de uma FAV pode levar a uma redução modesta no tamanho e na massa do ventrículo esquerdo, além de retardar a progressão da DRC em alguns casos.^288^

### Hipertensão Secundária em Diálise

Pacientes em diálise que se mantêm dentro dos critérios consagrados de HAR/HARf (ver item 8.2) são candidatos a avaliação para possíveis causas subjacentes, além da própria condição de DRC, que por si só poderia justificar a dificuldade de manejo pressórico^5^. Raramente serão encontrados pacientes com causas secundárias endocrinológicas ou não, e particularmente devem ser lembrados o uso de medicamentos que podem resultar no descontrole da PA, tais como anti-inflamatórios não esteroidais, eritropoetina recombinante humana e corticoides^84^. Outra causa não endocrinológica muito comum entre renais crônicos, incluindo mais de 50% daqueles em HD e DP, é a AOS, que deve ser reconhecida e tratada^78,289,290^. Todas as possíveis etiologias devem ser pensadas e pesquisadas em pacientes selecionados clínica e laboratorialmente, após ter-se obtido o peso seco adequado.

## Complicações da HA nos Pacientes em Diálise

As complicações da HA nos pacientes em diálise são eminentemente CV e se encontram no espectro das lesões de órgãos-alvo da HA em pacientes com função renal normal (Quadro 9). No entanto, devido a múltiplos outros mecanismos de lesão CV na DRC, como inflamação sistêmica, disfunção endotelial, calcificação arterial, hiperparatireoidismo, anemia, sobrecarga salina e presença de FAV, é difícil demonstrar a relação independente entre a HA e as complicações CV na DRC dialítica. Há pouquíssimos estudos relatando melhora dessas complicações com o tratamento da HA, o que é fator limitante de conclusões.

**Quadro 9:** Complicações cardiovasculares da hipertensão arterial na doença renal crônica dialítica

Insuficiência cardíaca
Doença coronariana
Morte cardíaca súbita
Fibrilação atrial
Acidente vascular cerebral
Doença arterial obstrutiva periférica (incluindo território carotídeo)
Aneurismas da aorta (torácica, abdominal)
Oclusão da artéria central da retina ou da veia central da retina

A mais importante associação é a com desfechos CV clinicamente manifestos, como a IC, a DAC, morte súbita cardíaca e AVC. A relação entre a HA e eventos CV na DRC dialítica é complexa. Estudos indicam associação de forma U entre a PA obtida na unidade de diálise e eventos CV coronarianos e cerebrais^3^ e com medidas ambulatoriais de PA em DP^291^. Os eventos ocorrem com as taxas mais altas em pacientes com PAS pré-diálise abaixo de 110–120 mmHg. A incidência de eventos mais baixa se encontra em valores de PAS entre 140–160 mmHg, e ocorre um aumento discreto, mas significativo, acima desses níveis. Há duas possíveis explicações com evidências para esse paradoxo. Uma se refere ao método de aferição da PA, especialmente a medida fora da unidade de diálise, já que alguns estudos sugerem que o uso da MAPA em vez da PA peridialítica transforma a relação U numa relação linear mais semelhante ao que se observa em pacientes hipertensos sem doença renal^3,5^,^99,292,293^. Analogamente, na DP, a ausência de relação linear entre PA e desfechos CV passa a ser positiva quando baseada em valores de PAS obtidos por MAPA^294^. A outra explanação é ligada à coexistência de disfunção cardíaca em pacientes com PA mais baixa. Um estudo mostrou que, em pacientes avaliados por MAPA, uma análise que excluiu os pacientes com IC ou FA transformou uma relação em U em uma relação linear positiva entre PA e desfechos^295^.

A HVE é uma complicação comum da HA que com frequência antecede o aparecimento da IC e predispõe a arritmias causais da morte cardíaca súbita. Apesar da presença de vários outros fatores na gênese da HVE em diálise, a HA é um fator causal independente em vários estudos^296^. Um estudo recente mostrou que a HVE em pacientes em HD aumentou progressivamente com o aumento da PAS pré-diálise (1,9× entre 131–139 mmHg, 7,7× entre 140–149 mmHg, e 12,9× se > 150 mmHg)^297^. Infelizmente, não é claro que a redução da PA resulta em melhora da HVE em pacientes em diálise. No estudo BID (*BP in Dialysis*), não houve diferenças na massa do VE entre pacientes randomizados ao controle intensivo da PA (PAS 115–140 mmHg) *vs*. grupo controle (PAS 155–165 mmHg), após um ano de seguimento^178^. Por outro lado, restrição salina e diálise prolongada, duas intervenções que resultam em melhor controle pressórico, proporcionam regressão da HVE em pacientes em diálise^298^. Os BRA e IECA resultam em regressão da HVE em pacientes em HD e PD^299^, o que não é o caso com bloqueadores dos receptores da aldosterona^300^. Não está definido se esse efeito é mediado pela redução da PA ou por outros mecanismos.

A FA tem prevalência média de 11,6% e incidência anual média de 2,7% na DRC dialítica^301^. Enquanto a relação entre HA e FA é forte em pacientes sem DRC, essa associação em diálise não é consistente entre as publicações. Entre os maiores estudos avaliando o problema, houve aumento do risco de FA crônica de aproximadamente 22% em pacientes com HA num estudo do USRDS^302^, enquanto dois estudos de outros bancos de dados (*Medicare/Medicaid* e DOPPS)^303,304^ demonstraram diminuição de risco de FA na presença de HA, tanto de forma binária (diminuição de 21%)^304^ quanto linear (diminuição de 6% para cada 10 mmHg de aumento da PAS pré-HD)^303^. Não há estudos analisando o impacto do tratamento da HA sobre a incidência da FA em pacientes em diálise.

A DAOP é comum em pacientes com DRC. A relação entre HA e DAOP em pacientes em diálise é inconsistente. No estudo CRIC, a HA não foi um marcador de risco para DAOP^305^. Por outro lado, o estudo DOPPS mostrou uma relação significativa entre HA e prevalência de DAOP em pacientes em HD^306^. Não há evidências de que o tratamento da HA tenha qualquer impacto no aparecimento ou regressão da DAOP na DRC. Por extrapolação, faz sentido que pacientes com aneurismas da aorta tenham manuseio com controle de impulso (redução tanto da PA quanto da FC).

A oclusão de vasos retinianos (artéria central, veia central) é complicação associada a vários fatores de risco, incluindo a HA. A oclusão da artéria central da retina é 4,5 vezes mais frequente na DRC dialítica do que em pacientes com função renal normal. Da mesma forma, a oclusão da veia central da retina, entidade mais comum que a oclusão da artéria central, ocorre com frequência 2,6 vezes maior em pacientes em diálise^307^. Em estudos retrospectivos com análises multivariadas, a HA encontra-se independentemente associada a ambas as complicações^308,309^.

Principais mensagens:

- Recomenda-se, para diminuir o risco complicações cardiovasculares em pacientes dialíticos hipertensos, evitar a PAS pré-diálise < 120 ou > 160 mmHg (Classe IIA/ Nível B).

## Conclusões

O diagnóstico e o manejo da HA na DRC dialítica são mais complexos ainda do que para a população geral. Diferentes determinantes fisiopatológicos estão envolvidos na HA da DRC 5D, com a sobrecarga de sódio e volume desempenhando um papel preponderante. Tecnologias para essa análise precisam mostrar-se seguras e factíveis.

Há maior variabilidade no ritmo circadiano da PA e a MAPA de 44 horas parece conferir o melhor diagnóstico e informações prognósticas, mas a MRPA é melhor tolerada e pode ser um substituto útil onde os recursos são limitados. ECR devem ser conduzidos em longo prazo com essas ferramentas diagnósticas e de acompanhamento para determinar sua importância e significância em desfechos.

Hiper e hipotensão intradialítica e fora da diálise são maléficas e devem ser evitadas.

Intervenções não farmacológicas, como restrição dietética de sódio, prolongamento do tempo de diálise e alteração da prescrição de diálise para melhorar a difusão de sódio, podem auxiliar no manejo da HA e impactam sintomas, a qualidade de vida e complicações cardiovasculares.

Quando se alcança o peso seco, e isso é insuficiente para o controle da HA, a farmacoterapia anti-hipertensiva é recomendada. Embora meta-análises evidenciem benefício de sobrevida com a terapia, nenhum estudo mostrou cabalmente benefício de uma classe de anti-hipertensivos em relação a outra. São necessários grandes ECR comparativos *head to head* para elucidar o tratamento medicamentoso ideal da HA em diálise, uma lacuna a ser preenchida pelos pesquisadores do mundo todo.

Deve-se individualizar o tratamento e as metas pressóricas considerando as melhores práticas, custo/benefício, dialisância dos medicamentos, idade, fragilidade, vulnerabilidade, comorbidades, polifarmácia, além das prioridades e preferências do paciente.

## SIGLAS

AMPA: automedida da pressão arterial
AOS: apneia obstrutiva do sono
APR: atividade plasmática de renina
ARM: antagonistas dos receptores mineralocorticoides
AVC: acidente vascular cerebral
BB: betabloqueadores
BCC: bloqueadores dos canais lentos de cálcio
BNP: peptídeo natriurético tipo B
BRA: bloqueadores dos receptores da angiotensina II
CV: cardiovascular
DAC: doença arterial coronariana
DAOP: doença arterial obstrutiva periférica
DC: débito cardíaco
DCV: doença cardiovascular
DE: disfunção endotelial
DM: *diabetes mellitus*
DMAA: dimetil-arginina-assimétrica
DP: diálise peritoneal
DRC: doença renal crônica
DRC 5D: doença renal crônica com paciente em diálise
ECR: estudos controlados e randomizados
ET1: endotelina 1
EPO: eritropoetina recombinante humana
FA: fibrilação atrial
FRR: função residual renal
GPID: ganho de peso interdialítico
HA: hipertensão arterial
HAB: hipertensão do avental branco
HABNC: hipertensão do avental branco não controlada
HAD: hipertensão arterial no paciente em diálise
HAS: hipertensão arterial sistólica
HC: hipertensão controlada
HD: hemodiálise
HDF: hemodiafiltração *on-line* de alto volume convectivo
HID: hipertensão intradialítica
HM: hipertensão mascarada
HMNC: hipertensão mascarada não controlada
HN: hipertensão noturna
HS: hipertensão sustentada
HSNC: hipertensão sustentada não controlada
HVE: hipertrofia ventricular esquerda
IAM: infarto agudo do miocárdio
IC: insuficiência cardíaca
IECA: inibidores da enzima de conversão da angiotensina
IMC: índice de massa corporal
IT: inércia terapêutica
KDIGO: KIDNEY DISEASES IMPROVING GLOBAL OUTCOMES
MAPA: monitorização ambulatorial da pressão arterial
MRPA: monitorização residencial da pressão arterial
NT-proBNP: peptídeo natriurético N-terminal pro-tipo B
NV: normotensão verdadeira
ON: óxido nítrico
PA: pressão arterial
PAC: pressão arterial central
PAD: pressão arterial diastólica
PAS: pressão arterial sistólica
PP: pressão de pulso
PS: peso seco
RA: rigidez arterial
RVP: resistência vascular periférica
SBN: Sociedade Brasileira de Nefrologia
SNC: sistema nervoso central
SNS: sistema nervoso simpático
SRAA: sistema renina angiotensina aldosterona
TFG: taxa de filtração glomerular
TRS: terapia renal substitutiva
UF: ultrafiltração
VD: ventrículo direito
VE: ventrículo esquerdo
